# Global Antimicrobial Resistance Trends in Group B *Streptococcus* Isolates From Pregnant Women: Systematic Review and Meta‐Analysis

**DOI:** 10.1002/mbo3.70087

**Published:** 2025-10-30

**Authors:** Narjess Bostanghadiri, Negar Narimisa, Mobina Kouhzad, Delaram Nourollahi, Afagh Shafiezadeh, Tahereh Navidifar

**Affiliations:** ^1^ Research Center for Clinical Virology Tehran University of Medical Sciences Tehran Iran; ^2^ Department of Microbiology School of Medicine Iran University of Medical Sciences Tehran Iran; ^3^ Department of Genetics Faculty of Science Islamic Azad University North Tehran Branch Tehran Iran; ^4^ Department of Basic Sciences Shoushtar Faculty of Medical Sciences Shoushtar Iran

**Keywords:** antibiotic resistance, global prevalence, Group B *Streptococcus*, meta‐analysis, *Streptococcus agalactiae*, systematic review

## Abstract

Maternal colonization with Group B *Streptococcus* (GBS) is associated with a high risk of serious infections during pregnancy. Also, the emerging resistance of GBS isolates to commonly used antibiotics has become a serious concern. This study aimed to provide a systematic review and meta‐analysis of worldwide data on the prevalence of antimicrobial resistance of GBS in pregnant women to establish a better understanding of more effective antibiotic agents. A systematic literature search was performed in PubMed, Web of Science, and Scopus databases up to January 31, 2025. Statistical analysis was performed using the meta and metaphor packages in R, and sources of heterogeneity were evaluated using *I* ^2^. The potential for publication bias was explored using Egger's and Begg's tests. A total of 169 eligible studies were included. The highest rate of resistance was associated with tetracycline (77.8%), while the lowest rates were found for vancomycin (0.59%), penicillin (0.7%), ampicillin (1%), and cefazolin (1.34%). The global resistance rates to penicillin, erythromycin, vancomycin, and clindamycin exhibited a gradual increasing trend in the 2021–2024 periods compared with other periods. The results highlighted the low levels of resistance to ampicillin and penicillin for pregnant women without an allergy history and cefazolin and vancomycin for those with an allergy history. However, given the increasing resistance trends and the high prevalence of resistance to clindamycin and erythromycin, these antibiotics should be recommended after antibiotic susceptibility testing.

## Graphical abstract

Group B *Streptococcus* in pregnant women shows high resistance to tetracycline and increasing resistance to clindamycin and erythromycin, while penicillin, ampicillin, cefazolin, and vancomycin remain effective. Findings highlight the need for antibiotic susceptibility testing to guide prophylaxis and treatment.

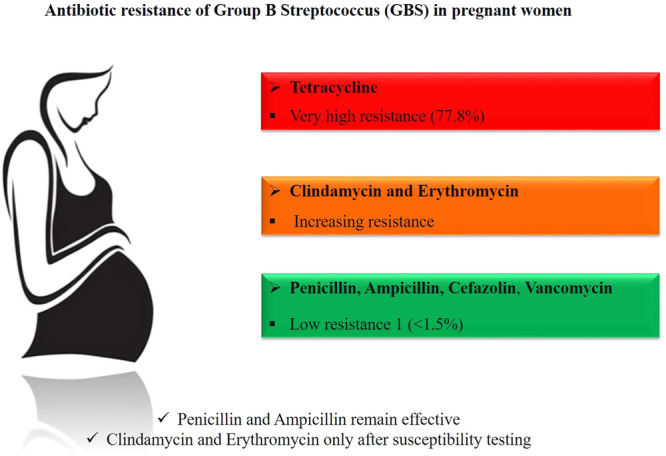

## 1 Introduction

The gastrointestinal tract serves as a reservoir for Group B *Streptococcus* (GBS), also known as *Streptococcus agalactiae*, and is likely the primary source of vaginal colonization. Maternal colonization plays a key role in the transmission of GBS, especially in early‐onset infections. The most frequent risk factors for vaginal or rectal GBS colonization during delivery are GBS bacteriuria during the ongoing pregnancy and a history of GBS colonization in a prior pregnancy (Patras and Nizet 2018). Transmission of GBS can occur either vertically via ascending intrauterine infection or during delivery via aspiration of contaminated amniotic or vaginal fluids by the newborn (Puopolo et al. 2019). Approximately 30%–70% of mothers colonized with GBS have delivered newborns with this organism, particularly when mothers have been colonized heavily with GBS; their offspring are at high risk for developing early‐onset infections (Shabayek and Spellerberg 2018).

The American College of Obstetricians and Gynecologists (ACOG) endorses routine GBS screening for all pregnant women between 36 and 37 weeks of gestation. Subsequently, intrapartum antibiotic prophylaxis (IAP) for women confirmed as GBS carriers (Puopolo et al. 2019; Verani et al. 2010). Notably, screening‐based IAP is more effective than risk‐based approaches. Still, this prevention strategy has several limitations, including its failure to prevent late‐onset GBS infections, stillbirths, and preterm births, as well as risks for disrupting the neonatal microbiome, the emergence of antimicrobial resistance, and implementation challenges in developing countries due to low‐resource settings (Jury et al. 2021; Garcia 2021). ACOG guidelines recommend penicillin as the antibiotic of choice for prophylaxis because of its narrow spectrum of activity, which reduces the emergence of antimicrobial resistance. For women with penicillin allergies, the selection of IAP requires a more accurate assessment of allergy status and, when possible, the results of clindamycin susceptibility testing. First‐generation cephalosporins are recommended as a first‐line alternative for women with a low‐risk penicillin allergy, due to both their maintained efficacy against GBS and minimal cross‐reactivity. Cefazolin is typically used as the standard cephalosporin for IAP (Snider et al. 2023; Puopolo et al. 2019). However, in women with high‐risk penicillin allergy and clindamycin‐resistant GBS strains, intravenous vancomycin is suggested as a choice for IPA. Nevertheless, clinical findings suggest exercising caution when using vancomycin due to safety concerns, including nephrotoxicity and the development of antibiotic resistance (Hayes et al. 2020). It is important to note that 40% of pregnant GBS‐positive women become culture‐negative at delivery, leading to unnecessary antibiotic exposure and the risk of antibiotic resistance (Morgan et al. 2025). Moreover, the emerging resistance to some antibiotics among colonizing GBS strains in pregnant women due to IAP has become a serious concern throughout Europe, North America, and Asia (Sabroske et al. 2023). It has highlighted an urgent global need for an antibiotic stewardship program. For example, Botelho et al. (2018) demonstrated in an 8‐year study in Brazil that GBS strains isolated from pregnant women had high rates of resistance to tetracycline. In contrast, resistance to chloramphenicol, clindamycin, erythromycin, and levofloxacin remained low (2%–14%). Zakerifar et al. (2023) in Iran demonstrated a high rate of resistance to tetracycline, ofloxacin, and erythromycin in GBS strains isolated from pregnant women. In contrast, resistance to chloramphenicol, vancomycin, and gentamicin was low (ranging from 7% to 13%). Due to the significant heterogeneity in GBS antimicrobial resistance profiles, influenced by geographic diversity and methodological variations in laboratory testing, a systematic meta‐analytic approach is crucial to clarify the available evidence and optimize global prophylaxis strategies. Hence, this study primarily aimed to conduct a systematic review and meta‐analysis of worldwide data on the prevalence of GBS antimicrobial resistance. Secondary objectives were to address trends over time, emerging resistance patterns, variation in resistance rates across regions, and to assess the possible effects of diagnostic methods and clinical guidelines on the reported data. By addressing these objectives, we aim to fill critical knowledge gaps and establish a better understanding of antimicrobial resistance in GBS.

## 2 Methods

### 2.1 Search Strategy and Study Selection

Studies focused on *S. agalactiae* antimicrobial resistance were identified through a systematic search of online databases, including MEDLINE (PubMed), Web of Science, and Scopus (January 2025). The following search syntax was used for a PubMed and other database search. A comprehensive search was conducted using the terms “*Streptococcus agalactiae*,” “*S. agalactiae*,” “antibiotic resistance,” “Pregnancy,” and all relevant keywords without any restrictions during database searching. The search syntax is described in Supporting Information File 1. We used Mesh Terms to determine synonyms. This review was performed and documented in compliance with the guidelines of the Preferred Reporting Items for Systematic Reviews and Meta‐Analyses (PRISMA) (Page et al. 2021). This adherence was underscored by our registration with the Prospero Registry (CRD420251059671), affirming our commitment to transparency and methodological integrity. The records found through database searching were merged, and the duplicates were removed using EndNote 20 (Thomson Reuters, New York, NY, USA). To prevent bias, two reviewers independently screened the records by title/abstract and full text to exclude the irrelevant articles. The third author investigated any disparities.

### 2.2 Data Extraction and Selection Criteria

All qualified studies were extracted and sorted into an Excel spreadsheet (Microsoft, Redmond, WA): first author's name, publication date, country, continent, sample collection date, sample size for studied antibiotics (the total number of *S. agalactiae* strains collected, and the number/fraction of resistant isolates to each antibiotic), antibiotic susceptibility test methodology (disk diffusion, dilution method, and automated system), and interpretative guidelines used (CLSI, EUCAST, Other) (Table S1). To mitigate the possibility of inaccuracies in the data extraction, three authors, M.K., D.N., and A.S.H., independently extracted the necessary data and reached an agreement on any discrepant findings.

Eligibility criteria for incorporating articles in the meta‐analysis were a report on the proportion of antibiotic resistance and a determined sample size. The following factors determined exclusion: (1) *S. agalactiae* was not detected, (2) *S. agalactiae* was isolated from animals or the environment, (3) *S. agalactiae* was taken from nonpregnant individuals, (4) *S. agalactiae* antibiotic resistance was not presented or only superficially reported as MIC50/90, (5) evaluation of the combined effects of antibiotics only, (6) when there was no transparent reporting of resistance rates, (7) data were from conference abstracts, editorials, prior meta‐analyses, systematic reviews, narrative reviews, and (8) failure to access full articles even after repeated attempts to establish contact with the corresponding author via electronic mail.

### 2.3 Quality Assessment

Two of the authors assessed the research quality using a modified version of the Joanna Briggs Institute (JBI) assessment tool, tailored specifically for cross‐sectional studies (Table S1). When disagreements arose, a third reviewer was responsible for resolving the issues.

### 2.4 Statistical Analysis

The data were analyzed using the meta and metaphor packages in R (version 4.4.3). We employed the inverse variance method to aggregate the effect sizes from the studies. The Freeman–Tukey double arcsine transformation method was applied to the proportional data. Using a random‐effects model, we calculated the pooled prevalence of GBS resistance to the antibiotics being studied, along with the corresponding 95% confidence intervals (CIs). To assess heterogeneity among the studies, we utilized the *I* ^2^ statistic. We also conducted Egger's and Begg's tests to evaluate publication bias, defining statistical significance at *p* < 0.05. Additionally, subgroup meta‐analyses were conducted based on various factors, including the period of bacterial isolation, continent, country, antimicrobial susceptibility method, and guidelines. Results were reported only for subgroups that demonstrated statistically significant differences.

## 3 Results

### 3.1 Search Results

**Figure 1:**
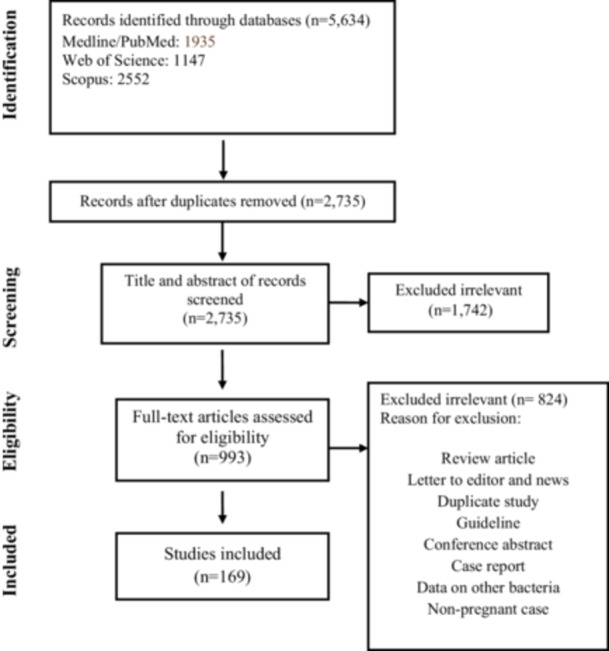
Flowchart of study selection for inclusion in the systematic review and meta‐analysis.

The process of article selection is characterized in Figure 1. A total of 5634 articles were identified through a search of the three aforementioned electronic databases. After removing duplicates, the titles and abstracts of 2735 articles were screened. Of these, 993 met the inclusion criteria and were retained for full‐text review. Of the 933 studies, 824 were excluded, and ultimately, a total of 169 studies were finally eligible and included in the present systematic review and meta‐analysis (Table S1 and Supporting Information File 2).

### 3.2 Characteristics of the Included Studies

Overall, the analysis included a total of 169 studies conducted up to January 31, 2025. These articles exhibited a broad geographical distribution, with research conducted across multiple regions worldwide. The interpretation of susceptibility testing was performed based on different guidelines and subsequently various breakpoints. Among them, the Clinical & Laboratory Standards Institute (CLSI) was the most widely employed. The quality assessment of the included studies was performed using the JBI critical appraisal checklist, and all 169 studies received high‐quality scores. Susceptibility testing to erythromycin, penicillin, clindamycin, and clindamycin was performed in most studies (*n* = 159, 83.68%; *n* = 146, 76.84%; *n* = 140, 73.84%, respectively) included in the meta‐analysis.

### 3.3 Meta‐Analysis Results

The resistance rate to different antimicrobial agents is presented in Table 1. Additionally, the results of subgroup analyses by continent, country, year of publication, method of susceptibility testing, and guidelines are presented in Supporting Information File 3 including Figures 1–8.

**Table 1:** Prevalence of antibiotic resistance in *Streptococcus agalactiae* isolates.

Antibiotic	No. of studies	Pooled prevalence	Lower CI	Upper CI	Events	Overall	*I* ^2^ (%)	*p* value
Penicillin	146	0.007	0.0016	0.0152	237	30,272	86.00	< 0.001
Ampicilin	100	0.01	0.0043	0.0389	281	19,689	92.90	< 0.001
Ceftriaxone	41	0.0222	0.0052	0.0468	106	6457	90.30	< 0.001
Cefotaxime	41	0.0458	0.0075	0.1065	177	7465	95.60	< 0.001
Cefazolin	15	0.0134	0	0.0517	40	1686	85.70	< 0.001
Cefepime	13	0.0254	0	0.1135	59	5179	95.80	< 0.001
Cefuroxime	8	0.0575	0	0.2278	43	3914	96.70	< 0.001
Cephalothin	4	0	0	0.0024	1	527	30.50	0.2291
Clindamycin	140	0.2	0.16	0.23	6298	27,423	97.20	< 0.001
Lincomycin	3	0.3124	0.0312	0.6993	92	458	97.90	< 0.001
Erythromucin	159	0.2151	0.1819	0.2502	7549	30,841	98.00	< 0.001
Azithromycin	13	0.3127	0.1898	0.4501	346	1159	94.60	< 0.001
Clarithromycin	4	0.3654	0.1043	0.6781	196	740	96.80	< 0.001
Vancomycin	106	0	0	0.0141	153	21,905	84.70	< 0.001

According to the results provided in Table S2, the highest resistance rate was showed for tetracycline (77.8% in 65 studies), Trimethoprim–Sulfamethoxazole (39. 31% in 19 studies), clarithromycin (36.54% in four studies), gentamycin (33.36% in 14 studies), lincomycin (31.24% in three studies), erythromycin (21.51% in 159 studies), and clindamycin (20% in 140 studies). The resistance rates to meropenem (in 12 studies), linezolid (in 29 studies), and cephalothin (in four studies) were not exhibited in any studies. Also, the low resistance rates were found for tigecycline (0.12% in four studies), rifampin (0.28% in four studies), ampicillin (0.7% in 146 studies), qn (0.98% in seven studies), penicillin (1% in 100 studies), cefazolin (1.34% in 15 studies), ceftriaxone (2.2% in 41 studies), cefotaxime (4.5% in 41 studies), and cefuroxime (5.7% in four studies).

### 3.4 Penicillins

A total of 146 articles documented the resistance of clinical GBS isolates from pregnant women to penicillin. The analysis indicated a resistance rate of 0.0% (95% CI, 0.0%–1.9%; *I* ^2^ = 86%; *p* < 0.001). However, Egger's test (*p* < 0.001) suggested the existence of publication bias across the studies, prompting a trim and fill analysis (Table 2).

**Table 2:** Publication bias of meta‐analysis of antibiotic resistance in *Streptococcus agalactiae* isolates.

Antibiotic	No. of studies	Pooled prevalence	Adjusted pooled prevalence	Studies added	*p* value of Egger's test	*p* value of Begg's test
Penicillin	146	0.007 (95%: 0.0016–0.0152)	0.0319 (95%: 0.0127–0.0512)	70	< 0.001	< 0.001
Ampicilin	100	0.01 (95%: 0.0043–0.389)	0.0357 (95%: 0.094–0.227)	48	< 0.001	< 0.001
Ceftriaxone	41	0.0222 (95%: 0.0052–0.0468)	0.0329 (95%: 0.0414–0.1073)	21	< 0.001	< 0.001
Cefotaxime	41	0.0458 (95%: 0.0075–0.1065)	0.0335 (95%: 0.0855–0.1527)	21	< 0.001	< 0.001
Cefazolin	15	0.0134 (95%: 0.0–0.0517)	0.0100 (95%: 0.0251–0.2252)	2	0.2105	0.0174
Cefepime	13	0.0254 (95%: 0.0–0.1135)	0.0297 (95%: 0.0145–0.2047)	7	0.0641	0.01
Cefuroxime	8	0.0575 (95%: 0.0–0.2278)	0.0575 (95%: 0.0–0.2278)	0	0.679	0.1087
Cephalothin	4	0 (95%: 0.0–0.0024)	0.03 (95%: 0.0–0.11)	2	0.0226	0.3333
Clindamycin	140	0.2 (95%: 0.16–0.23)	0.2 (95%: 0.16–0.23)	0	0.31	0.0002
Lincomycin	3	0.3124 (95%: 0.0311–0.6993)	0.3124 (95%: 0.0311–0.6993)	0	0.6615	0.1361
Erythromycin	159	0.2151 (95%: 0.1819–0.2502)	0.2151 (95%: 0.1819–0.2502)	0	0.8886	0.0458
Azithromycin	13	0.3127 (95%: 0.1898–0.4501)	0.3127 (95%: 0.1898–0.4501)	0	0.4118	0.7599
Clarithromycin	4	0.3654 (95%: 0.1043–0.6781)	0.3654 (95%: 0.1043–0.6781)	0	0.3943	0.333
Vancomycin	106	0.00 (95%: 0.00–0.0141)	0.028 (95%: 0.008–0.065)	53	< 0.001	< 0.001

Subgroup analysis revealed that Africa had the highest resistance rate among the continents, at 7% (*p* < 0.001). Within individual countries, Nigeria exhibited the highest resistance at 28%, followed by Ethiopia at 14% (*p* = 0.005). Additionally, the analysis of guidelines showed that CLSI, with a 1% resistance rate, demonstrated higher resistance compared with other guidelines (*p* < 0.001). The disk diffusion method also showed a higher resistance rate of 1% compared with other assay techniques (*p* < 0.001). Notably, we observed a 5% increase in penicillin resistance from the period before 2001 to 2021–2024 (*p* < 0.001). A total of 100 articles documented the resistance of clinical GBS isolates from pregnant women to penicillin. The analysis indicated a resistance rate of 1% (95% CI, 0.43%–3.89%; *I* ^2^ = 92.9%; *p* < 0.001). However, Egger's test (*p* < 0.001) suggested the existence of publication bias across the studies, prompting a trim and fill analysis (Table 2). Subgroup analysis revealed that Africa had the highest resistance rate among the continents, at 4% (*p* < 0.001). Within individual countries, Nigeria exhibited the highest resistance at 47%, followed by Iran at 20% (*p* < 0.001). The disk diffusion method also showed a higher resistance rate of 3% compared with other assay techniques (*p* < 0.001). Notably, we observed a 22% increase in Ampicillin resistance from the period before 2001 to 2021–2024 (*p* < 0.001).

### 3.5 Cephalosporins

A total of 41 articles documented the resistance of clinical GBS isolates from pregnant women to Ceftriaxone. The analysis indicated a resistance rate of 2.22% (95% CI, 0.52%–4.68%, *I* ^2^ = 90.30%, *p* < 0.001). However, Egger's test (*p* < 0.001) suggested the existence of publication bias across the studies, prompting a trim and fill analysis (Table 2). Subgroup analysis revealed that Africa had the highest resistance rate among the continents, at 6% (*p* = 0.0432). Within individual countries, Iran exhibited the highest resistance at 26%, followed by Ethiopia at 17% (*p* < 0.001). Additionally, the analysis of guidelines showed that CLSI, with a 3% resistance rate, demonstrated higher resistance compared with other guidelines (*p* = 0.0138). The disk diffusion method also showed a higher resistance rate of 4% compared with other assay techniques (*p* = 0.0019). A total of 41 articles documented the resistance of clinical GBS isolates from pregnant women to Cefotaxime. The analysis indicated a resistance rate of 4.58% (95% CI, 0.75%–10.65%; *I* ^2^ = 95.60%; *p* < 0.001). However, Egger's test (*p* < 0.001) suggested the existence of publication bias across the studies, prompting a trim and fill analysis (Table 2).

Subgroup analysis revealed that Africa had the highest resistance rate among the continents, at 11% (*p* = 0.014). Within individual countries, Iran exhibited the highest resistance at 80%, followed by Nigeria at 35% (*p* = 0.0124). The disk diffusion method also showed a higher resistance rate of 10% compared with other assay techniques (*p* = 0.007). Notably, we observed a 78% increase in Cefotaxime resistance from the period before 2001 to 2021–2024 (*p* < 0.001). A total of 15 articles documented the resistance of clinical GBS isolates from pregnant women to Cefazolin. The analysis indicated a resistance rate of 1.34% (95% CI, 0.0%–5.17%; *I* ^2^ = 85.7%; *p* < 0.001). Nonetheless, Egger's test (*p* = 0.21) indicated that there is no evidence of publication bias among the studies. A total of 13 articles documented the resistance of clinical GBS isolates from pregnant women to Cefepime. The analysis indicated a resistance rate of 2.54% (95% CI, 0.0%–5.17%; *I* ^2^ = 95.8%; *p* < 0.001). Nonetheless, Egger's test (*p* = 0.64) indicated that there is no evidence of publication bias among the studies.

Subgroup analysis revealed that Africa had the highest resistance rate among the continents, at 32% (*p* = 0.036). Within individual countries, Ethiopia exhibited the highest resistance at 59% (*p* < 0.001). The disk diffusion method also showed a higher resistance rate of 4% compared with other assay techniques (*p* < 0.001). A total of 8 articles documented the resistance of clinical GBS isolates from pregnant women to Cefuroxime. The analysis indicated a resistance rate of 5.75% (95% CI, 0.0%–22.78%; *I* ^2^ = 96.7%; *p* < 0.001). However, Egger's test (*p* = 0.679) suggested the existence of publication bias across the studies, prompting a trim and fill analysis (Table 2).

A total of four articles documented the resistance of clinical GBS isolates from pregnant women to Cephalothin. The analysis indicated a resistance rate of 0% (95% CI, 0.0%–0.24%; *I* ^2^ = 30.5%; *p* = 0.229). However, Egger's test (*p* = 0.022) suggested the existence of publication bias across the studies, prompting a trim and fill analysis (Table 2).

### 3.6 Lincosamides

A total of 140 articles documented the resistance of clinical *GBS* isolates from pregnant women to Clindamycin. The analysis indicated a resistance rate of 20% (95% CI, 16%–23%; *I* ^2^ = 97.20%; *p* < 0.001). However, Egger's test (*p* = 0.31) indicated that there is no evidence of publication bias among the studies.

Subgroup analysis revealed that Asia had the highest resistance rate among the continents, at 28% (*p* = 0.004). Within individual countries, South Africa exhibited the highest resistance at 53%, followed by China at 49% (*p* < 0.001). Notably, we observed a 25% increase in Clindamycin resistance from the period before 2001 to 2021–2024 (*p* < 0.001). A total of three articles documented the resistance of clinical GBS isolates from pregnant women to Lincomycin. The analysis indicated a resistance rate of 31.24 (95% CI, 3.12%–69.93%; *I* ^2^ = 97.90%; *p* < 0.001). However, Egger's test (*p* = 0.66) indicated that there is no evidence of publication bias among the studies.

### 3.7 Macrolides

A total of 159 articles documented the resistance of clinical GBS isolates from pregnant women to Erythromycin. The analysis indicated a resistance rate of 21.51% (95% CI, 18.19%–25.02%; *I* ^2^ = 98%; *p* < 0.001). However, Egger's test (*p* = 0.88) indicated that there is no evidence of publication bias among the studies.

Subgroup analysis revealed that Asia had the highest resistance rate among the continents, at 32% (*p* < 0.001). Within individual countries, China exhibited the highest resistance at 63%, followed by South Africa at 48% (*p* < 0.001). Notably, we observed a 22% increase in Erythromycin resistance from the period before 2001 to 2021–2024 (*p* < 0.001). A total of 13 articles documented the resistance of clinical GBS isolates from pregnant women to Azithromycin. The analysis indicated a resistance rate of 31.27% (95% CI, 18.98%–45.01%; *I* ^2^ = 96.6%; *p* < 0.001). However, Egger's test (*p* = 0.41) indicated that there is no evidence of publication bias among the studies. Subgroup analysis revealed that Asia had the highest resistance rate among the continents, at 52% (*p* = 0.0181).

### 3.8 Glycopeptide

A total of 106 articles documented the resistance of clinical GBS isolates from pregnant women to Vancomycin. The analysis indicated a resistance rate of 0.0% (95% CI, 0.00%–1.41%; *I* ^2^ = 84.70%; *p* < 0.001). However, Egger's test (*p* < 0.001) suggested the existence of publication bias across the studies, prompting a trim and fill analysis (Table 2). Subgroup analysis revealed that Africa had the highest resistance rate among the continents, at 2% (*p* < 0.001). Within individual countries, Nigeria exhibited the highest resistance at 24% (*p* = 0.0013). Notably, we observed a 14% increase in Vancomycin resistance from the period before 2001 to 2021–2024 (*p* = 0.0005).

## 4 Discussion

GBS is a significant pathogen of perinatal infectious diseases, causing 10% of preterm births. It can colonize the pregnant woman and subsequently cause invasive, early‐onset disease in neonates, leading to death (Wang et al. 2023).

The increasing rates of resistance to common antibiotics recommended for treating GBS infections have become a global concern. This can undermine effective prevention in pregnant women with a history of penicillin allergy, resulting in the increased risk for both mothers and their newborns. In addition, rising resistance to macrolides and lincosamides has limited drug options, particularly in women with penicillin allergy, which can further complicate the prevention of maternal and neonatal GBS infection. Resistant GBS strains also may increase the likelihood of early‐onset and late‐onset neonatal sepsis, especially if intrapartum prophylaxis fails, resulting in increased morbidity and mortality. Because of the rising resistance and limitations of antibiotic prophylaxis, the importance of local surveillance to inform prophylaxis strategies and maternal GBS vaccination is being recognized as two promising long‐term strategies (Capanna et al. 2013; Carvalho et al. 2025; Garland et al. 2011).

The increasing rates of resistance to common antibiotics recommended for treating GBS infections have become a global concern. This necessitates a comprehensive understanding of the prevalence of antibiotic resistance in this organism. In this meta‐analysis, we aim to provide an in‐depth evaluation of the global prevalence of antibiotic resistance in GBS isolated from pregnant women. According to the AOCG guideline, penicillin is regarded as an antibiotic with a narrow spectrum that exhibits its antimicrobial activity against Gram‐positive bacteria. Therefore, there is a low potential of inducing resistance in other vaginal microorganisms (Gynecologists ACoOa 2020). Penicillin and ampicillin are two of the most commonly used antibiotics for treating and preventing GBS infections. They are administered intravenously to pregnant women during labor (Nielsen et al. 2025). Data obtained from this meta‐analysis indicated a high sensitivity of GBS isolates to these two antibiotics worldwide (penicillin, 0.7% out of 46 studies, and ampicillin, 1% out of 100 studies). According to the subgroup analysis of continents, Asia and Europe had the highest number of isolates tested for susceptibility to penicillin and ampicillin; however, the resistance rates to these two antibiotics were very low, ranging from 0% to 2%. The present findings are consistent with those of Wang et al. (2023), who conducted a meta‐analysis in China between 2009 and 2019. They reported a very low prevalence of resistance to ampicillin (ranging from 0% to 2.7%) and penicillin (ranging from 0% to 1.1%) among pregnant women colonized with GBS isolates in seven different regions of China. In a similar meta‐analysis conducted in Iran, Khademi and Sahebkar (2020) reported a low prevalence of resistance to ampicillin (2.7%) and penicillin (4.2%) among pregnant women with GBS‐colonized infections. In addition, the global meta‐analysis conducted by Hsu et al. (2025) indicated a low prevalence of resistance to ampicillin (3.1%) and penicillin (1.7%) among patients with GBS infections.

Africa had the lowest number of GBS isolates tested for susceptibility to penicillin and ampicillin compared with other continents, but the pooled resistance rates were higher (7% for penicillin and 4% for ampicillin). Furthermore, Ethiopia had the highest percentage of penicillin‐resistant GBS isolates (14% out of 532 isolates), and Nigeria had the highest percentage of ampicillin‐resistant isolates (47% out of 57 isolates). In contrast to our study, a meta‐analysis conducted among African pregnant women between 1989 and 2019 by Gizachew et al. (2019) reported a relatively high overall prevalence of resistance to ampicillin and penicillin (a resistance rate of 33.5% for penicillin from nine studies and 26.78% for ampicillin from eight studies). This discrepancy between our findings and those analyzed by Gizachew et al. (2019). This can be explained by the differences in the periods encompassed by these two meta‐analyses. While Gizachew et al. (2019). Our meta‐analysis, which included studies conducted from 1989 to 2019, covered a broader and more recent period from 1990 to 2024. This higher range may have included more recent studies with enhanced diagnostic methods, better antibiotic stewardship, or shifts in bacterial resistance patterns, which could account for the lower pooled resistance rates in the present meta‐analysis rather than the Gizachew et al. (2019) meta‐analysis.

The prevalence of resistance to penicillin has increased slightly over time: 0% before 2015, 3% from 2016 to 2020, and 5% from 2021 to 2024 (*p* < 0.0001). In parallel with our findings, a Japanese study also confirmed an increase in penicillin resistance from 2.3% to 14.7% between 2005 and 2013 (Seki et al. 2015). Similar to penicillin, the ampicillin resistance rate is increasing over time, from a range of 0%–1% before 2015, to 6% between 2016 and 2020, and 22% during 2021–2024. The greater increase in ampicillin resistance as compared with penicillin may also be attributable to differences in usage patterns of these antibiotics, with ampicillin potentially being prescribed more extensively in recent years. Additionally, the limited number of isolates analyzed from 2021 to 2024 may have had a substantial influence on the increased ampicillin resistance during this period. Hence, further research is necessary to determine the extent of this influence.

Cephalosporin antibiotics are recommended as relatively safe agents for the prophylaxis and treatment of GBS infections in pregnant women with a low risk of penicillin allergy (ACOG 2020). In this meta‐analysis, the resistance rates to several cephalosporin antibiotics were analyzed, including cefazolin, ceftriaxone, ceforuxime, cephalothin, cefotaxime, and cefepime. Cefazolin is the most commonly used cephalosporin for the prophylaxis of GBS infections (ACOG 2020); however, the resistance has only been evaluated in 15 studies, with a rate of 1.3%. Notably, the rate of resistance has remained consistent across different periods (*p* > 0.05). Similarly, Huang et al. conducted a meta‐analysis of 16 studies and found a low prevalence of cefazolin‐resistant GBS isolates in pregnant women, ranging from 0% to 3.7%. In contrast, Khademi and Sahebkar (2020) have demonstrated a higher prevalence of cefazolin‐resistant GBS isolates in pregnant women (7.5% vs. 1.5%).

Among cephalosporins, more studies have evaluated resistance to ceftriaxone and cefotaxime than to other cephalosporins (41 studies each). However, these antibiotics are not commonly used to prevent GBS infections in pregnant women. Our findings indicated a low rate of GBS isolates resistant to ceftriaxone and cefotaxime (2.2% and 4.5%, respectively). Similar to ampicillin and penicillin, Africa had the highest prevalence of resistant isolates for ceftriaxone and cefotaxime (6% out of 753 isolates and 11% out of 635 isolates, respectively), particularly in Ethiopia, with a rate of 17% ceftriaxone‐resistant isolates and Nigeria with a rate of 35% cefotaxime–resistant GBS isolates in pregnant women. Similar to our finding, Huang et al. (2016) reported in a global meta‐analysis of 28 studies that GBS isolates are highly susceptible to cefotaxime, except in Egypt, where the rate was 7.4%. In comparison to other countries, Iran has a high prevalence of resistance to cefotaxime, estimated at 80%. However, this finding is based on a limited number of reports and cannot be generalized to the entire country. Therefore, further studies are needed to accurately assess the antibiotic resistance of GBS in Iran.

On the other hand, the meta‐analysis conducted by Huang et al. (2016) indicated significant heterogeneity in resistance rates to ceftriaxone. The resistance rates ranged from 0% in studies conducted in the United States and Turkey to 13% and 15.6% in Tanzania (Africa) and South Korea, respectively. In addition, two meta‐analyses conducted by Gizachew et al. (2019) and Wadilo et al. (2023) have demonstrated a higher prevalence of ceftriaxone‐resistant GBS isolates in Africa than the findings of the present meta‐analysis; furthermore, Gizachew et al. (2019) indicated a rate of 26%, based on 12 studies from 1989 to 2019, whereas Wadilo et al. (2023) indicated a rate of 16.33%, based on 12 studies from 2000 to 2022. This high prevalence can be attributed to several factors, including regional discrepancies in antibiotic use strategies, insufficient availability of antimicrobial stewardship protocols, and the overuse or misuse of broad‐spectrum cephalosporins on this continent.

Clindamycin is considered a significant second‐line antibiotic for treating GBS infections in patients with severe penicillin allergies. The prevalence of resistance was evaluated in the majority of studies included in this meta‐analysis (20% of 140 studies). Consistent with the findings of this study, Huang et al. (2016) reported a 27.1% prevalence of resistance to clindamycin in pregnant women.

Subgroup analysis by continent revealed that Asia had the highest rate of clindamycin‐resistant isolates (28%). In contrast, Europe had the most significant number of GBS isolates evaluated for resistance to this antibiotic (11,200 isolates), with a rate of 17%. A subsequent analysis of the countries subgroup has demonstrated the highest rates of clindamycin‐resistant isolates in South Africa (53%) and China (49%) among GBS isolates from pregnant women. Consistent with our data, Wang et al. (2023) conducted a meta‐analysis in China, indicating a high prevalence and heterogeneity across different regions, ranging from 33.6% to 74.3% (based on 60 studies).

In the present meta‐analysis, the pooled prevalence of resistance to clindamycin in Africa was 19% which is relatively similar to two meta‐analyses conducted on this continent by Gizachew et al. (2019) (19.6% based on 22 studies) and Wadilo et al. (2023) (24.1% based on 23 studies). Therefore, clindamycin appears to be a suitable and effective treatment option for pregnant women colonized or infected with GBS isolates. Additionally, this meta‐analysis shows that Italy has the highest number of tested GBS isolates for clindamycin susceptibility.

A gradual upward trend in clindamycin resistance was identified in the present analysis, with increasing resistance from 27% during the 2016–2020 period to 32% during the 2021–2024 period (*p* < 0.0001). Notably, the number of strains studied during the 2021–2024 period was lower than in the previous 5‐year periods, which may be attributed to the shorter 4‐year time frame. In addition, an increasing trend in resistance was observed, with an approximate more than twofold increase during the period of 2006–2010 compared with the period of 2011–2015 (14% vs. 25%, respectively). Consistent with our findings, clindamycin resistance in the USA increased from 37.0% in 2011 to 43.2% in 2016 (Francois Watkins et al. 2019). In addition, in a meta‐analysis in China, clindamycin resistance showed a decreasing trend from 66% in 2017 to 56.7% in 2020; however, a slight increase in resistance to this antibiotic was observed in 2021 (59.7%) (Wang et al. 2023). These findings suggest that there is an alarm in these countries due to the misuse or excessive use of this antibiotic to treat GBS infections. Therefore, the implementation of control measures is strongly advised. These measures include the routine use of the d‐test method for the simultaneous identification of antibiotic susceptibility to clindamycin and erythromycin, the establishment of national regular surveillance programs, and the use of alternative antibiotics, such as vancomycin, to treat GBS infections effectively.

Erythromycin is the second most crucial second‐line antibiotic for the prophylaxis of GBS infections in patients with severe penicillin allergies. The prevalence of resistance to erythromycin was evaluated in almost all the studies included in this meta‐analysis (21.5% out of 159 studies). Similar to the analysis of the continent subgroup for clindamycin, Asia had the highest rate of erythromycin‐resistant isolates (32%); however, Europe exhibited the highest number of GBS isolates tested for erythromycin susceptibility, with a resistance rate of 15%, similarly, as with the analysis of country subgroups for clindamycin, the highest rates of erythromycin‐resistant isolates were found in China (63% out of 2853) and South Africa (48% out of 331). In addition, Italy was again recognized as a significant country in the evaluation of antibiotic susceptibility of GBS isolates to erythromycin, accounting for approximately one‐sixth of all isolates analyzed. Of these, 21% exhibited resistance to the antibiotic. Consistent with our findings, two meta‐analyses on Iranian (Khademi and Sahebkar 2020) and African (Wadilo et al. 2023) pregnant women also found a relatively similar prevalence of erythromycin resistance, at 25.1% and 26%, respectively. However, another meta‐analysis study found a high prevalence of erythromycin‐resistant GBS isolates throughout China, ranging from 55.1% in Southeast China to 82.4% in Northeast China (Wang et al. 2023). Overall, erythromycin resistance is increasing worldwide. In addition, erythromycin does not effectively pass the placenta to achieve adequate concentrations in the amniotic fluid; hence, more interest has been attracted to alternative antibiotic agents (Heikkinen et al. 2000).

Consistent with the analysis of the subgroup of period for clindamycin, the present study identified a gradual increasing trend in erythromycin resistance. The resistance increased from 30% during the 2016–2020 period to 31% during the 2021–2024 period (*p* < 0.001). However, it should be noted that the number of strains studied during the 2021–2024 period was lower than in the previous 5‐year periods, which may have limited the ability to detect significant trends. Despite the gradual increase in observed resistance over time, a 2.5‐fold increase was found from 11% in 2006–2010 to 27% in 2011–2015. A meta‐analysis study conducted in China has revealed that the prevalence of erythromycin‐resistant GBS isolates remained relatively stable from 2017 to 2021, with a range of 72.7%–74.5%, except in 2016, when it was slightly lower at 69.2% (Wang et al. 2023). The observed increase in erythromycin resistance can be explained by several factors, including excessive use of macrolides, the development of resistance genes to macrolides, such as *erm* and *mef*, and the high prevalence of resistant clones (Domelier et al. 2008).

Vancomycin, as the only pharmacokinetically and microbiologically validated option, is administered to women at high risk for penicillin allergies and to those infected with clindamycin‐resistant GBS isolates. However, vancomycin has significant adverse effects, such as hypersensitivity reactions and nephrotoxicity. Therefore, it is usually recommended only for the treatment of serious Gram‐positive infections (ACOG 2020). This meta‐analysis evaluated the vancomycin susceptibility in 106 studies and found an extremely low resistance rate of 0.59%. The highest number of GBS isolates tested for susceptibility to vancomycin was found in Asia and Europe, with resistance rates of 0% and 1%, respectively. However, Africa exhibited the highest prevalence of resistance (2% out of 1518 isolates), particularly in Nigeria, where 24% of 71 isolates were vancomycin‐resistant. High susceptibility of GBS isolates to vancomycin has been reported in several meta‐analyses (Wang et al. 2023; Huang et al. 2016; Khademi and Sahebkar 2020; Wadilo et al. 2023). However, a meta‐analysis study on African pregnant women demonstrated a relatively high prevalence of resistance to this antibiotic, at 19% (Gizachew et al. 2019). The emergence of vancomycin‐resistant isolates may be attributed to the widespread use of this antibiotic empirically in the treatment of certain infectious diseases, as well as the easy availability and relatively inexpensive cost of this antibiotic in these regions (Gizachew et al. 2019). Hence, the prescription of vancomycin for GBS infections should be performed with caution, as inappropriate use of this drug can lead to the emergence of resistant organisms, which can lead to serious health concerns (ACOG 2020).

According to the analysis of the subgroup of the period, there was no resistance up to 2015. However, from 2016 to 2020, the resistance rate was 2%, and then it dropped to 1.4% from 2021 to 2024. A stable trend of vancomycin resistance has also been reported among Chinese pregnant women, based on evidence obtained by a meta‐analysis conducted from 2017 to 2021 (Wang et al. 2023).

Tetracycline showed the highest rate of resistance among antibiotics in GBS isolates (77.8% based on 65 studies). ACOG does not recommend this antibiotic for the treatment or prophylaxis of GBS in pregnant women. Consistent with our findings, other meta‐analyses have also confirmed the notable prevalence of vancomycin‐resistant GBS isolates among pregnant women (Huang et al. 2016; Wang et al. 2023; Gizachew et al. 2019; Khademi and Sahebkar 2020). The high rates of tetracycline resistance in human GBS isolates can be explained by two main factors, including genetic determinants (e.g., *tet(M), tet(O)*, and *tet(T)* gene), and the extensive use of tetracycline in previous that resulted in global dissemination of tetracycline‐resistant GBS clones that are well‐adapted to human hosts. In addition, the transfer of antibiotic resistance determinants between human‐ and bovine‐associated GBS strains may occur; however, more research is needed to provide a comprehensive understanding of this mechanism (Dogan et al. 2005).

In this meta‐analysis, the disk diffusion was the most commonly used method for antibiotic susceptibility testing, likely due to its simplicity and ease of implementation. However, MIC determination is crucial for selecting the most suitable antibiotic and for epidemiological reporting.

## 5 Conclusion

According to the results of this meta‐analysis, penicillin and ampicillin remain the most effective antibiotics for the prophylaxis and treatment of GBS infections, as resistance rates to these antibiotics are very low globally. In addition, due to the high susceptibility of most GBS isolates to cefazolin and vancomycin, these antibiotics are recommended for patients with mild or severe allergies to first‐line antibiotics. Clindamycin and erythromycin have been commonly prescribed in recent years for the prophylaxis of GBS; however, due to the increasing global resistance, especially in certain countries, such as China, the use of these two antibiotics should be performed after antimicrobial susceptibility testing of GBS isolates. In this meta‐analysis, Africa exhibited higher levels of antibiotic resistance across most antibiotics compared with other continents. Hence, regular monitoring and surveillance programs are essential to track the antimicrobial susceptibility patterns of GBS isolates in this region.

## Author Contributions

**Narjess Bostanghadiri:** conceptualization (equal), methodology (equal), data curation (equal), formal analysis (equal), writing – original draft (equal), writing – review and editing (equal). **Negar Narimisa:** formal analysis (lead), visualization (equal). **Mobina Kouhzad:** investigation (equal), data curation (equal). **Delaram Nourollahi:** investigation (equal), data curation (equal). **Afagh Shafiezadeh:** investigation (equal), data curation (equal). **Tahereh Navidifar:** conceptualization (equal), methodology (equal), data curation (lead), formal analysis (equal), writing – original draft (lead), writing – review and editing (lead), supervision (lead).

## Ethics Statement

The authors have nothing to report.

## Consent

The authors have nothing to report.

## Conflicts of Interest

The authors assert that the conducted investigation was free from any economic or monetary affiliations that could be perceived as a potential conflict of interest.

## Supporting information


**Supporting File 1:** Search Strategy and Syntax.

**Supporting File 2:** References of included studies.

**Supporting File 3:** Results of Subgroup Analyses.

**Supporting Table S1:** Study Characteristics and Extracted Variables.

**Supporting Table S2:** Detailed results of meta‐analysis and subgroup analyzing.

## Data Availability

The data that support the findings of this study are available in the Supporting Information of this article.
