# Supplementary material for: Global Antimicrobial Resistance Trends in Group B Streptococcus Isolates From Pregnant Women: Systematic Review and Meta‐Analysis
Source: Microbiologyopen. 2025 Oct 30;14(6):e70087. doi: 10.1002/mbo3.70087 (PMC12575007; doi:10.1002/mbo3.70087)
Supplement: Supplementary file 1 — Supporting File 1: Search Strategy and Syntax. [file MBO3-14-e70087-s001.docx]

**(“Streptococcus agalactiae” OR S. agalactiae OR “Streptococcus Group B” OR GBS) AND ("sensitivity tests, microbial" OR "antimicrobial susceptibility breakpoint determination" OR "test, microbial sensitivity" OR "tests, microbial sensitivity" OR "drug sensitivity assay, microbial" OR "microbial sensitivity test" OR "sensitivity test, microbial" OR "breakpoint determination, antimicrobial susceptibility" OR "antibacterial susceptibility breakpoint determination" OR "minimum inhibitory concentration" OR "concentration, minimum inhibitory" OR "concentrations, minimum inhibitory" OR "breakpoint determination, antibacterial susceptibility" OR "inhibitory concentration, minimum" OR "inhibitory concentrations, minimum" OR "minimum inhibitory concentrations" OR "antibiogram*" OR "bacterial sensitivity tests" OR "tests, bacterial sensitivity" OR "sensitivity test, bacterial" OR "sensitivity tests, bacterial" OR "test, bacterial sensitivity" OR "bacterial sensitivity test" OR "antibiotic resistance" OR "drug resistance, microbial" OR "antimicrobial resistance" OR resistant* OR susceptible*) AND (Pregnancy OR Pregnant)**

**Jaunaury 31, 2025**

**Pubmed 1935**

**Scopus 2557**

**WOS 1147**

**After 3549**
