# Supplementary material for: Global Antimicrobial Resistance Trends in Group B Streptococcus Isolates From Pregnant Women: Systematic Review and Meta‐Analysis
Source: Microbiologyopen. 2025 Oct 30;14(6):e70087. doi: 10.1002/mbo3.70087 (PMC12575007; doi:10.1002/mbo3.70087)
Supplement: Supplementary file 2 — Supporting File 2: References of included studies. [file MBO3-14-e70087-s002.docx]

(Mohamadi, et al. 2017, Ojo, et al. 2019, Numanović, et al. 2015, Hadavand, et al. 2015, Yılmaz Karadağ, et al. 2013, Beauruelle, et al. 2018, Otaguiri, et al. 2018, Ngonzi, et al. 2018, Nkembe, et al. 2018, Kamilia, et al. 2021, Mosca, et al. 2006, Abdallah, et al. 2021, Abdullah, et al. 2023, Abotorabi, et al. 2023, Ábrók, et al. 2019, Joachim, et al. 2009, Akpaka, et al. 2022, Alemseged, et al. 2015, Alfouzan, et al. 2021, Ali, et al. 2020, AL-Subol, et al. 2021, AlZuheiri, et al. 2021, Domelier, et al. 2008, Muller, et al. 2008, Hannoun, et al. 2009, Arisoy, et al. 2003, Assefa, et al. 2018, González Pedraza Avilés, et al. 2002, de Azavedo, et al. 2001, Dhanoa, et al. 2010, Lee, et al. 2010, Rojo-Bezares, et al. 2016, Back, et al. 2012, Bae, et al. 2022, Barcaite, et al. 2012, Barros, et al. 2016, Beauruelle, et al. 2018, Beigverdi, et al. 2014, Belard, et al. 2015, Berkowitz, et al. 1990, Bhola, et al. 2020, Bland, et al. 2001, Bob-Manuel, et al. 2021, Panda, et al. 2009, Capanna, et al. 2013, Chen, et al. 2024, Cheng, et al. 2020, Choi, et al. 2012, Church, et al. 2012, Choi, et al. 2021, Creti, et al. 2017, Curry, et al. 2018, Marchaim, et al. 2006, Savoia, et al. 2008, Vinnemeier, et al. 2015, Castellano-Filho, et al. 2010, Dilrukshi, et al. 2023, Egbule, et al. 2024, Ernest, et al. 2015, Edwards, et al. 2002, Fahim, et al. 2022, Fantahun, et al. 2020, Feuerschuette, et al. 2022, Florindo, et al. 2014, Fröhlicher, et al. 2014, Fu, et al. 2004, Ge, et al. 2021, Genovese, et al. 2020, Gharabeigi, et al. 2023, Girma, et al. 2020, Gizachew, et al. 2019, Goudarzi, et al. 2015, Grimwood, et al. 2002, Guo, et al. 2019, Haimbodi, et al. 2021, Husen, et al. 2023, Barbaros, et al. 2005, Iweriebor, et al. 2023, Dilrukshi, et al. 2021, Africa and Kaambo 2018, Jamrozy, et al. 2023, Jannati, et al. 2012, Ji, et al. 2017, Jiao, et al. 2022, Jones, et al. 2022, Simoes, et al. 2007, Marimón, et al. 2005, Hayes, et al. 2017, Kamińska, et al. 2024, Kawaguchiya, et al. 2022, Khan, et al. 2015, Kimura, et al. 2013, Kunze, et al. 2015, Decoster, et al. 2005, Lee, et al. 2023, Motlova, et al. 2004, Leykun, et al. 2021, Li, et al. 2023, Liakopoulos, et al. 2014, Liu, et al. 2022, Lusta, et al. 2023, Madrid, et al. 2018, Kawaguchiya, et al. 2022, Chohan, et al. 2006, Strus, et al. 2009, Manning, et al. 2003, Makled, et al. 2020, Matalka, et al. 2021, Matani, et al. 2016, MELO, et al. 2016, Mengist, et al. 2016, Mengist, et al. 2017, Mohamed, et al. 2020, Mohammed, et al. 2012, Morales, et al. 1999, Al-Sweih, et al. 2005, Orrett 2003, Oviedo, et al. 2013, Moroi, et al. 2019, Mukesi, et al. 2019, Botelho, et al. 2018, Nasri, et al. 2013, Ngom, et al. 2023, Njoku, et al. 2017, Petca, et al. 2024, Qadi, et al. 2021, Rostami, et al. 2021, Burcham, et al. 2019, Quiroga, et al. 2008, Shabayek, et al. 2009, Sadaka, et al. 2018, Santana, et al. 2020, El Shahaway, et al. 2019, Sherman, et al. 2012, Shore, et al. 2012, Shrestha, et al. 2020, Tor-Udom, et al. 2006, Spaetgens, et al. 2002, Stylianopoulos, et al. 2002, Garland, et al. 2011, Slotved, et al. 2021, Soares, et al. 2013, Souza, et al. 2013, Ki, et al. 2012, El-Kersh, et al. 2012, Elsaid Tash and Ahmed 2019, Teatero, et al. 2017, Tesfaye, et al. 2022, Le TRAN, et al. 2021, Tsai, et al. 2019, Tsolia, et al. 2003, Lavergne, et al. 2006, Wang, et al. 2015, Warrier, et al. 2022, Brzychczy-Włoch, et al. 2013, Woldu, et al. 2014, Bolukaoto, et al. 2015, Yan, et al. 2016, Yeung, et al. 2014, Seo, et al. 2010, Zhang, et al. 2021, Zhou, et al. 2023, Joao, et al. 2011, Carvalho, et al. 2024, Capanna, et al. 2013, Eskandarian, et al. 2015, Meehan, et al. 2014, Paccione, et al. 2013, Lee, et al. 2015)

**References**

Abdallah, Noha G, Faten M Ali, Lamiaa A Adel*, et al.* "Prevalence, Resistance Profile and Virulence Genes of Streptococcus Agalactiae Colonizing near-Term Pregnant Women Attending Ain Shams University Hospital." **15**, no. 3 (2021)

Abdullah, Omnia Mohamed, Gehan Saddik Elhadidy, Rasha Elsayed Khamiss*, et al.* "Identification of Inducible Clindamycin Resistance Gene in Streptococcus Agalactiae Isolates Colonizing Pregnant Women in Suez Canal University Hospitals." **4**, no. 4 (2023): 1246-54

Abotorabi, Shokoh, Zohreh Rasooli, Hamideh Pakniat*, et al.* "Virulence Determinants, Serotypes, and Antimicrobial Resistance of Rectovaginal Isolates of Streptococcus Agalactiae." **18**, no. 1 (2023): 82

Ábrók, Marianna, Petra Tigyi, Markus Kostrzewa*, et al.* "Evaluation of the Results of Group B Streptococcus Screening by Maldi-Tof Ms among Pregnant Women in a Hungarian Hospital." **9**, no. 1 (2019): 1

Africa, Charlene WJ, and Eveline %J Frontiers in public health Kaambo. "Group B Streptococcus Serotypes in Pregnant Women from the Western Cape Region of South Africa." **6** (2018): 356

Akpaka, Patrick Eberechi, Khamiya Henry, Reinand Thompson*, et al.* "Colonization of Streptococcus Agalactiae among Pregnant Patients in Trinidad and Tobago." **3** (2022): 96-100

AL-Subol, Ibrahim Hasan, Maha Abdul-Aziz, Abdullah A Almikhlafy*, et al.* "An Initial Survey on the Prevalence of Group B Streptococcus (Gbs) among Yemeni Pregnant Women." (2021)

Al-Sweih, N, M Jamal, M Kurdia*, et al.* "Antibiotic Susceptibility Profile of Group B Streptococcus (Streptococcus Agalactiae) at the Maternity Hospital, Kuwait." **14**, no. 4 (2005): 260-63

Alemseged, Gebreselassie, Selam Niguse, Haftamu Hailekiros*, et al.* "Isolation and Anti-Microbial Susceptibility Pattern of Group B Streptococcus among Pregnant Women Attending Antenatal Clinics in Ayder Referral Hospital and Mekelle Health Center, Mekelle, Northern Ethiopia." **8** (2015): 1-8

Alfouzan, Wadha, Nahed Gaddar, Rita Dhar*, et al.* "A Study of Group B Streptococcus in Pregnant Women in Lebanon: Prevalence, Risk Factors, Vaginal Flora and Antimicrobial Susceptibility." **4**, no. 7 (2021)

Ali, Musa Mohammed, Yimtubezinash Woldeamanuel, Daniel Asrat*, et al.* "Features of Streptococcus Agalactiae Strains Recovered from Pregnant Women and Newborns Attending Different Hospitals in Ethiopia." **20** (2020): 1-9

AlZuheiri, Shatha Taher Salman, Rajani Dube, Godfred Menezes*, et al.* "Clinical Profile and Outcome of Group B Streptococcal Colonization in Mothers and Neonates in Ras Al Khaimah, United Arab Emirates: A Prospective Observational Study." **9**, no. 3 (2021): 235-40

Arisoy, AS, B Altinişik, Ö Tünger*, et al.* "Maternal Carriage and Antimicrobial Resistance Profile of Group B Streptococcus." **31** (2003): 244-46

Assefa, S., K. Desta, and T. Lema. "Group B Streptococci Vaginal Colonization and Drug Susceptibility Pattern among Pregnant Women Attending in Selected Public Antenatal Care Centers in Addis Ababa, Ethiopia." *BMC Pregnancy Childbirth* **18**, no. 1 (May 4 2018): 135.<https://doi.org/10.1186/s12884-018-1791-4>

Back, E. E., E. J. O'Grady, and J. D. Back. "High Rates of Perinatal Group B Streptococcus Clindamycin and Erythromycin Resistance in an Upstate New York Hospital." *Antimicrob Agents Chemother* **56**, no. 2 (Feb 2012): 739-42.<https://doi.org/10.1128/aac.05794-11>

Bae, H. G., J. Hong, Y. J. Kim*, et al.* "A Retrospective National Study on Colonization Rate and Antimicrobial Susceptibility of Streptococcus Agalactiae in Pregnant Korean Women, 2018-2020." *Yonsei Med J* **63**, no. 8 (Aug 2022): 717-23.<https://doi.org/10.3349/ymj.2022.63.8.717>

Barbaros, Ilikkan, Carikci Murat, Vural Mehmet*, et al.* "The Colonization Incidence of Group B Streptococcus in Pregnant Women and Their Newborns in Istanbul." **47**, no. 1 (2005): 64-66

Barcaite, E., A. Bartusevicius, R. Tameliene*, et al.* "Group B Streptococcus and Escherichia Coli Colonization in Pregnant Women and Neonates in Lithuania." *Int J Gynaecol Obstet* **117**, no. 1 (Apr 2012): 69-73.<https://doi.org/10.1016/j.ijgo.2011.11.016>

Barros, R. R., A. F. de Souza, and F. B. Luiz. "Polyclonal Spread of Streptococcus Agalactiae Resistant to Clindamycin among Pregnant Women in Brazil." *J Antimicrob Chemother* **71**, no. 7 (Jul 2016): 2054-6.<https://doi.org/10.1093/jac/dkw085>

Beauruelle, C., A. Pastuszka, L. Mereghetti*, et al.* "Group B Streptococcus Vaginal Carriage in Pregnant Women as Deciphered by Clustered Regularly Interspaced Short Palindromic Repeat Analysis." *J Clin Microbiol* **56**, no. 6 (Jun 2018).<https://doi.org/10.1128/jcm.01949-17>

Beauruelle, Clemence, Adeline Pastuszka, Laurent Mereghetti*, et al.* "Group B Streptococcus Vaginal Carriage in Pregnant Women as Deciphered by Clustered Regularly Interspaced Short Palindromic Repeat Analysis." **56**, no. 6 (2018): 10.1128/jcm. 01949-17

Beigverdi, R., F. Jabalameli, A. Mirsalehian*, et al.* "Virulence Factors, Antimicrobial Susceptibility and Molecular Characterization of Streptococcus Agalactiae Isolated from Pregnant Women." *Acta Microbiol Immunol Hung* **61**, no. 4 (Dec 2014): 425-34.<https://doi.org/10.1556/AMicr.61.2014.4.4>

Belard, S., N. Toepfner, M. Capan-Melser*, et al.* "Streptococcus Agalactiae Serotype Distribution and Antimicrobial Susceptibility in Pregnant Women in Gabon, Central Africa." *Sci Rep* **5** (Nov 25 2015): 17281.<https://doi.org/10.1038/srep17281>

Berkowitz, K., J. A. Regan, and E. Greenberg. "Antibiotic Resistance Patterns of Group B Streptococci in Pregnant Women." *J Clin Microbiol* **28**, no. 1 (Jan 1990): 5-7.<https://doi.org/10.1128/jcm.28.1.5-7.1990>

Bhola, P., N. R. Mvelase, Y. Balakrishna*, et al.* "Antimicrobial Susceptibility Patterns of Uropathogens Isolated from Pregnant Women in Kwazulu-Natal Province: 2011 - 2016." *S Afr Med J* **110**, no. 9 (Aug 31 2020): 872-76.<https://doi.org/10.7196/SAMJ.2020.v110i9.14468>

Bland, M. L., S. T. Vermillion, D. E. Soper*, et al.* "Antibiotic Resistance Patterns of Group B Streptococci in Late Third-Trimester Rectovaginal Cultures." *Am J Obstet Gynecol* **184**, no. 6 (May 2001): 1125-6.<https://doi.org/10.1067/mob.2001.115478>

Bob-Manuel, M., L. McGee, J. A. Igunma*, et al.* "Whole Genome Sequence Based Capsular Typing and Antimicrobial Resistance Prediction of Group B Streptococcal Isolates from Colonized Pregnant Women in Nigeria." *BMC Genomics* **22**, no. 1 (Aug 23 2021): 627.<https://doi.org/10.1186/s12864-021-07929-z>

Bolukaoto, John Y, Charles M Monyama, Martina O Chukwu*, et al.* "Antibiotic Resistance of Streptococcus Agalactiae Isolated from Pregnant Women in Garankuwa, South Africa." **8** (2015): 1-7

Botelho, Ana Caroline N, Juliana G Oliveira, Andreia P Damasco*, et al.* "Streptococcus Agalactiae Carriage among Pregnant Women Living in Rio De Janeiro, Brazil, over a Period of Eight Years." **13**, no. 5 (2018): e0196925

Brzychczy-Włoch, Monika, Dorota Ochońska, and Małgorzata Bulanda. "Carriage of Group B Streptococci in Pregnant Women from the Region of Krakow and Their Antibiotic Resistance in the Years 2008-2012." (2013)

Burcham, Lindsey R, Brady L Spencer, Lauryn R Keeler*, et al.* "Determinants of Group B Streptococcal Virulence Potential Amongst Vaginal Clinical Isolates from Pregnant Women." **14**, no. 12 (2019): e0226699

Capanna, F., S. P. Emonet, A. Cherkaoui*, et al.* "Antibiotic Resistance Patterns among Group B Streptococcus Isolates: Implications for Antibiotic Prophylaxis for Early-Onset Neonatal Sepsis." *Swiss Med Wkly* **143** (2013): w13778.<https://doi.org/10.4414/smw.2013.13778>

Capanna, Federica, Stephane Paul Emonet, Abdessalam Cherkaoui*, et al.* "Antibiotic Resistance Patterns among Group B Streptococcus Isolates: Implications for Antibiotic Prophylaxis for Early-Onset Neonatal Sepsis." **143**, no. 1314 (2013): w13778-w78

Carvalho, Anjo Gabriel, Renata Santos Rodrigues, Mariana Delfino Rodrigues*, et al.* "Group B Streptococcus Colonization Prevalence and Susceptibility Profile in Pregnant Women in the Brazilian Amazon." **24** (2024): e20230063

Castellano-Filho, D. S., V. L. da Silva, T. C. Nascimento*, et al.* "Detection of Group B Streptococcus in Brazilian Pregnant Women and Antimicrobial Susceptibility Patterns." *Braz J Microbiol* **41**, no. 4 (Oct 2010): 1047-55.<https://doi.org/10.1590/s1517-838220100004000024>

Chen, Y., L. Liu, J. Liu*, et al.* "Serotype Distribution, Antimicrobial Resistance, and Molecular Characterization of Group B Streptococcus Isolates from Chinese Pregnant Woman." *J Matern Fetal Neonatal Med* **37**, no. 1 (Dec 2024): 2295805.<https://doi.org/10.1080/14767058.2023.2295805>

Cheng, Z., P. Qu, P. Ke*, et al.* "Antibiotic Resistance and Molecular Epidemiological Characteristics of Streptococcus Agalactiae Isolated from Pregnant Women in Guangzhou, South China." *Can J Infect Dis Med Microbiol* **2020** (2020): 1368942.<https://doi.org/10.1155/2020/1368942>

Chohan, Lubna, Lisa M Hollier, Karen Bishop*, et al.* "Patterns of Antibiotic Resistance among Group B Streptococcus Isolates: 2001–2004." **2006**, no. 1 (2006): 057492

Choi, S. J., J. Kang, and Y. Uh. "Recent Epidemiological Changes in Group B Streptococcus among Pregnant Korean Women." *Ann Lab Med* **41**, no. 4 (Jul 1 2021): 380-85.<https://doi.org/10.3343/alm.2021.41.4.380>

Choi, S. J., S. D. Park, I. H. Jang*, et al.* "The Prevalence of Vaginal Microorganisms in Pregnant Women with Preterm Labor and Preterm Birth." *Ann Lab Med* **32**, no. 3 (May 2012): 194-200.<https://doi.org/10.3343/alm.2012.32.3.194>

Church, D., J. Carson, and D. Gregson. "Point Prevalence Study of Antibiotic Susceptibility of Genital Group B Streptococcus Isolated from near-Term Pregnant Women in Calgary, Alberta." *Can J Infect Dis Med Microbiol* **23**, no. 3 (Fall 2012): 121-4.<https://doi.org/10.1155/2012/876103>

Creti, R., M. Imperi, A. Berardi*, et al.* "Neonatal Group B Streptococcus Infections: Prevention Strategies, Clinical and Microbiologic Characteristics in 7 Years of Surveillance." *Pediatr Infect Dis J* **36**, no. 3 (Mar 2017): 256-62.<https://doi.org/10.1097/inf.0000000000001414>

Curry, A., G. Bookless, K. Donaldson*, et al.* "Evaluation of Hibergene Loop-Mediated Isothermal Amplification Assay for Detection of Group B Streptococcus in Recto-Vaginal Swabs: A Prospective Diagnostic Accuracy Study." *Clin Microbiol Infect* **24**, no. 10 (Oct 2018): 1066-69.<https://doi.org/10.1016/j.cmi.2018.01.008>

de Azavedo, J. C., M. McGavin, C. Duncan*, et al.* "Prevalence and Mechanisms of Macrolide Resistance in Invasive and Noninvasive Group B Streptococcus Isolates from Ontario, Canada." *Antimicrob Agents Chemother* **45**, no. 12 (Dec 2001): 3504-8.<https://doi.org/10.1128/aac.45.12.3504-3508.2001>

Decoster, L., J. Frans, H. Blanckaert*, et al.* "Antimicrobial Susceptibility of Group B Streptococci Collected in Two Belgian Hospitals." *Acta Clin Belg* **60**, no. 4 (Sep-Oct 2005): 180-4.<https://doi.org/10.1179/acb.2005.032>

Dhanoa, A., R. Karunakaran, and S. D. Puthucheary. "Serotype Distribution and Antibiotic Susceptibility of Group B Streptococci in Pregnant Women." *Epidemiol Infect* **138**, no. 7 (Jul 2010): 979-81.<https://doi.org/10.1017/s0950268809991105>

Dilrukshi, G. N., J. Kottahachchi, Dmbt Dissanayake*, et al.* "Group B Streptococcus Colonisation and Their Antimicrobial Susceptibility among Pregnant Women Attending Antenatal Clinics in Tertiary Care Hospitals in the Western Province of Sri Lanka." *J Obstet Gynaecol* **41**, no. 1 (Jan 2021): 1-6.<https://doi.org/10.1080/01443615.2020.1716313>

Dilrukshi, N., J. Kottahachchi, T. Dissanayake*, et al.* "Antibiotic Sensitivity of Group B Streptococcus from Pregnant Mothers and Its Association with Resistance Genes." *Med Princ Pract* **32**, no. 2 (2023): 126-32.<https://doi.org/10.1159/000530525>

Domelier, Anne-Sophie, Nathalie van der Mee-Marquet, Laurence Arnault*, et al.* "Molecular Characterization of Erythromycin-Resistant Streptococcus Agalactiae Strains." **62**, no. 6 (2008): 1227-33

Edwards, Rodney K, Penny Clark, Patrick %J Obstetrics Duff*, et al.* "Intrapartum Antibiotic Prophylaxis 2: Positive Predictive Value of Antenatal Group B Streptococci Cultures and Antibiotic Susceptibility of Clinical Isolates." **100**, no. 3 (2002): 540-44

Egbule, O. S., O. F. Enwa, P. K. Omenogor*, et al.* "Multidrug Resistant Group B Streptococcus Isolates from Pregnant Women in Delta State, Nigeria." *Pak J Biol Sci* **27**, no. 9 (Aug 2024): 447-54.<https://doi.org/10.3923/pjbs.2024.447.454>

El-Kersh, TA, Salwa M Neyazi, Yazeed A Al-sheikh*, et al.* "Phenotypic Traits and Comparative Detection Methods of Vaginal Carriage of Group B Streptococci and Its Associated Micro-Biota in Term Pregnant Saudi Women." **6** (2012): 403-13

El Shahaway, Alia A, Hanaa M El Maghraby, Heba A Mohammed*, et al.* "Diagnostic Performance of Direct Latex Agglutination, Post-Enrichment Latex Agglutination and Culture Methods in Screening of Group B Streptococci in Late Pregnancy: A Comparative Study." (2019): 2583-88

Elsaid Tash, Rehab M, and Mohamed I %J Egyptian Journal of Medical Microbiology Ahmed. "Carriage of Streptococcus Agalactiae among Pregnant Women in an Egyptian University Hospital, Serotypes Distribution and Antibiotics Susceptibility." **28**, no. 3 (2019): 79-84

Ernest, Alex I, Edgar Ndaboine, Anthony Massinde*, et al.* "Maternal Vaginorectal Colonization by Group B Streptococcus and Listeria Monocytogenes and Its Risk Factors among Pregnant Women Attending Tertiary Hospital in Mwanza, Tanzania." **17**, no. 2 (2015)

Eskandarian, N, Z Ismail, V Neela*, et al.* "Antimicrobial Susceptibility Profiles, Serotype Distribution and Virulence Determinants among Invasive, Non-Invasive and Colonizing Streptococcus Agalactiae (Group B Streptococcus) from Malaysian Patients." **34** (2015): 579-84

Fahim, Noha Alaa Eldin, Mona Byoumee Ragay, Noha Nagi Salah El-Deen*, et al.* "Diagnostic Performance of Two Chromogenic Media for Streptococcus Agalactiae Screening in Pregnant Women." **3**, no. 4 (2022): 947-55

Fantahun, Yitbarek, Shemse Sebre, Aminu Seman*, et al.* "Magnitude of Maternal Vaginal Colonization of Group B Streptococcus and Neonatal Transmission in Pregnant Women during Labor and Delivery at Tikur Anbessa Specialized Hospital, Addis Ababa, Ethiopia." **58** (2020)

Feuerschuette, Otto Henrique May, Eduardo Venâncio Alves, Mara Cristina Scheffer*, et al.* "Genetic Diversity and Antimicrobial Resistance of Invasive, Noninvasive and Colonizing Group B Streptococcus Isolates in Southern Brazil." **4**, no. 6 (2022): 000370

Florindo, Carlos, Vera Damiao, Jorge Lima*, et al.* "Accuracy of Prenatal Culture in Predicting Intrapartum Group B Streptococcus Colonization Status." **27**, no. 6 (2014): 640-42

Fröhlicher, Simone, Gabriela Reichen-Fahrni, Martin Müller*, et al.* "Serotype Distribution and Antimicrobial Susceptibility of Group B Streptococci in Pregnant Women: Results from a Swiss Tertiary Centre." **144** (2014): w13935

Fu, Jung-Chung, Duei-Pong Lin, Wei-Jen Huang*, et al.* "Antibiotic Susceptibility Pattern of Anovaginal Isolates of Streptococcus Agalactiae from Pregnant Women in Their Late Third Trimester." **20**, no. 7 (2004): 330-33

Garland, Suzanne M, Erin Cottrill, Lisa Markowski*, et al.* "Antimicrobial Resistance in Group B Streptococcus: The Australian Experience." **60**, no. 2 (2011): 230-35

Ge, Yanmei, Fei Pan, Rui Bai*, et al.* "Prevalence of Group B Streptococcus Colonization in Pregnant Women in Jiangsu, East China." **21**, no. 1 (2021): 492

Genovese, Carlo, Floriana D’Angeli, Valentina Di Salvatore*, et al.* "Streptococcus Agalactiae in Pregnant Women: Serotype and Antimicrobial Susceptibility Patterns over Five Years in Eastern Sicily (Italy)." **39**, no. 12 (2020): 2387-96

Gharabeigi, Natalie, Akram Sadat Tabatabaee Bafroee, and Kumarss %J Iranian Journal of Medical Sciences Amini. "Molecular Serotyping and Antibiotic Resistance Profile of Group B Streptococcus Strains Isolated from Iranian Pregnant Women with Urinary Tract Infection." **48**, no. 6 (2023): 542

Girma, Woubishet, Nadia Yimer, Tesfaye Kassa*, et al.* "Group B Streptococcus Recto-Vaginal Colonization in near-Term Pregnant Women, Southwest Ethiopia." **30**, no. 5 (2020)

Gizachew, Mucheye, Moges Tiruneh, Feleke Moges*, et al.* "Streptococcus Agalactiae from Ethiopian Pregnant Women; Prevalence, Associated Factors and Antimicrobial Resistance: Alarming for Prophylaxis." **18** (2019): 1-9

González Pedraza Avilés, A., M. C. Ortiz Zaragoza, and R. Mota Vázquez. "Serotypes and Antimicrobial Susceptibility of Group B Streptococcus from Pregnant Women in Mexico." *Rev Latinoam Microbiol* **44**, no. 3-4 (Jul-Dec 2002): 133-6

Goudarzi, Gholamreza, Masoumeh Ghafarzadeh, Pegah Shakib*, et al.* "Culture and Real-Time Pcr Based Maternal Screening and Antibiotic Susceptibility for Group B Streptococcus: An Iranian Experience." **7**, no. 6 (2015): 233

Grimwood, Keith, Peter R Stone, Isobelle A Gosling*, et al.* "Late Antenatal Carriage of Group B Streptococcus by New Zealand Women." **42**, no. 2 (2002): 182-86

Guo, Huiwu, Maozhang Fu, Qing Peng*, et al.* "Antimicrobial Resistance and Molecular Characterization of Streptococcus Agalactiae from Pregnant Women in Southern China." **13**, no. 09 (2019): 802-09

Hadavand, Shahrzad, Fatemeh Ghafoorimehr, Leila Rajabi*, et al.* "Frequency of Group B Streptococcal Colonization in Pregnant Women Aged 35-37 Weeks in Clinical Centers of Shahed University, Tehran, Iran." **10**, no. 2 (2015): 120

Haimbodi, Erastus Lafimana, Munyaradzi Mukesi, and Sylvester Rodgers %J BMC microbiology Moyo. "Prevalence and Molecular Characterization of Group B Streptococcus in Pregnant Women from Hospitals in Ohangwena and Oshikoto Regions of Namibia." **21** (2021): 1-9

Hannoun, Antoine, Marwa Shehab, Marie-Therese Khairallah*, et al.* "Correlation between Group B Streptococcal Genotypes, Their Antimicrobial Resistance Profiles, and Virulence Genes among Pregnant Women in Lebanon." **2009**, no. 1 (2009): 796512

Hayes, K., L. Cotter, L. Barry*, et al.* "Emergence of the L Phenotype in Group B Streptococci in the South of Ireland." *Epidemiol Infect* **145**, no. 16 (Dec 2017): 3535-42.<https://doi.org/10.1017/s0950268817002461>

Husen, Oliyad, Moorthy Kannaiyan Abbai, Alqeer Aliyo*, et al.* "Prevalence, Antimicrobial Susceptibility Pattern and Associated Factors of Group B Streptococcus among Pregnant Women Attending Antenatal Care at Bule Hora University Teaching Hospital, Southern Ethiopia." (2023): 4421-33

Iweriebor, Benson C, Kayode O Afolabi, Pola Z Stofile*, et al.* "Group B Streptococcus Agalactiae Resistant to Recommended Intrapartum Antibiotic Prophylaxis Isolated from the Rectovaginal Area of 35-37 Weeks Pregnant Women." **19**, no. 5 (2023)

Jamrozy, Dorota, Guduru Gopal Rao, Theresa Feltwell*, et al.* "Population Genetics of Group B Streptococcus from Maternal Carriage in an Ethnically Diverse Community in London." **14** (2023): 1185753

Jannati, E, M Roshani, MOHSEN Arzanlou*, et al.* "Capsular Serotype and Antibiotic Resistance of Group B Streptococci Isolated from Pregnant Women in Ardabil, Iran." **4**, no. 3 (2012): 130

Ji, Wenjing, Lihua Zhang, Zhusheng Guo*, et al.* "Colonization Prevalence and Antibiotic Susceptibility of Group B Streptococcus in Pregnant Women over a 6-Year Period in Dongguan, China." **12**, no. 8 (2017): e0183083

Jiao, Jin, Weiwei Wu, Feng Shen*, et al.* "Clinical Profile and Risk Factors of Group B Streptococcal Colonization in Mothers from the Eastern District of China." **2022**, no. 1 (2022): 5236430

Joachim, Agricola, Mecky I Matee, Furaha A Massawe*, et al.* "Maternal and Neonatal Colonisation of Group B Streptococcus at Muhimbili National Hospital in Dar Es Salaam, Tanzania: Prevalence, Risk Factors and Antimicrobial Resistance." **9** (2009): 1-7

Joao, Esau C, Maria Isabel Gouvêa, Jacqueline A Menezes*, et al.* "Group B Streptococcus in a Cohort of Hiv-Infected Pregnant Women: Prevalence of Colonization, Identification and Antimicrobial Susceptibility Profile." **43**, no. 9 (2011): 742-46

Jones, Sandra, Peter Newton, Matthew Payne*, et al.* "Epidemiology, Antimicrobial Resistance, and Virulence Determinants of Group B Streptococcus in an Australian Setting." **13** (2022): 839079

Kamilia, Belhadi, Chayeb Farouk Zohir, and Djaara %J Age Hayat. "The First Prenatal Group B Strep (Gbs) Screening in Late Pregnancy in Algerian Population (Northeast Algeria)." **1**, no. 27 (2021): P2

Kamińska, D., M. Ratajczak, D. M. Nowak-Malczewska*, et al.* "Macrolide and Lincosamide Resistance of Streptococcus Agalactiae in Pregnant Women in Poland." *Sci Rep* **14**, no. 1 (Feb 16 2024): 3877.<https://doi.org/10.1038/s41598-024-54521-y>

Kawaguchiya, M., N. Urushibara, M. S. Aung*, et al.* "Molecular Characterization and Antimicrobial Resistance of Streptococcus Agalactiae Isolated from Pregnant Women in Japan, 2017-2021." *IJID Reg* **4** (Sep 2022): 143-45.<https://doi.org/10.1016/j.ijregi.2022.07.002>

Kawaguchiya, Mitsuyo, Noriko Urushibara, Meiji Soe Aung*, et al.* "Molecular Characterization and Antimicrobial Resistance of Streptococcus Agalactiae Isolated from Pregnant Women in Japan, 2017–2021." **4** (2022): 143-45

Khan, M. A., A. Faiz, and A. M. Ashshi. "Maternal Colonization of Group B Streptococcus: Prevalence, Associated Factors and Antimicrobial Resistance." *Ann Saudi Med* **35**, no. 6 (Nov-Dec 2015): 423-7.<https://doi.org/10.5144/0256-4947.2015.423>

Ki, M, U Srinivasan, KY Oh*, et al.* "Emerging Fluoroquinolone Resistance in Streptococcus Agalactiae in South Korea." **31** (2012): 3199-205

Kimura, K., K. Matsubara, G. Yamamoto*, et al.* "Active Screening of Group B Streptococci with Reduced Penicillin Susceptibility and Altered Serotype Distribution Isolated from Pregnant Women in Kobe, Japan." *Jpn J Infect Dis* **66**, no. 2 (2013): 158-60.<https://doi.org/10.7883/yoken.66.158>

Kunze, M., K. Zumstein, F. Markfeld-Erol*, et al.* "Comparison of Pre- and Intrapartum Screening of Group B Streptococci and Adherence to Screening Guidelines: A Cohort Study." *Eur J Pediatr* **174**, no. 6 (Jun 2015): 827-35.<https://doi.org/10.1007/s00431-015-2548-y>

Lavergne, V, M Laverdière, A Duchesne*, et al.* "Prenatal Culture-Based Screening of Streptococcus Agalactiae Colonisation: Resistance against Erythromycin and Clindamycin." **25** (2006): 532-34

Le TRAN, Thi Nhu, Thi Diem Kieu PHAM, LE Thi Gai*, et al.* "Antibiotic Resistance of Group B Streptococcus in Pregnant Women at 35-37 Weeks of Gestation in Southern Vietnam." (2021)

Lee, B. K., Y. R. Song, M. Y. Kim*, et al.* "Epidemiology of Group B Streptococcus in Korean Pregnant Women." *Epidemiol Infect* **138**, no. 2 (Feb 2010): 292-8.<https://doi.org/10.1017/s0950268809990859>

Lee, Wen-Tsung, Mei-Chin %J Journal of Microbiology Lai, Immunology, and Infection. "High Prevalence of Streptococcus Agalactiae from Vaginas of Women in Taiwan and Its Mechanisms of Macrolide and Quinolone Resistance." **48**, no. 5 (2015): 510-16

Lee, Y., H. G. Bae, D. Won*, et al.* "Comparative Analysis of the Molecular Characteristics of Group B Streptococcus Isolates Collected from Pregnant Korean Women Using Whole-Genome Sequencing." *Ann Lab Med* **43**, no. 2 (Mar 1 2023): 180-86.<https://doi.org/10.3343/alm.2023.43.2.180>

Leykun, Yasabe, Chalachew Genet, Wondemagegn %J Infection Mulu*, et al.* "Group B Streptococci Vaginal-Recto Colonization, Vertical Transmission to Newborns, Antimicrobial Susceptibility Profile and Associated Factors in Selected Health Facilities of Bahir Dar City: A Cross-Sectional Study." (2021): 5457-72

Li, Xiaoou, Wei Gao, Zhonglan Jia*, et al.* "Characterization of Group B Streptococcus Recovered from Pregnant Women and Newborns Attending in a Hospital in Beijing, China." (2023): 2549-59

Liakopoulos, Apostolos, Angeliki Mavroidi, Sofia Vourli*, et al.* "Molecular Characterization of Streptococcus Agalactiae from Vaginal Colonization and Neonatal Infections: A 4-Year Multicenter Study in Greece." **78**, no. 4 (2014): 487-90

Liu, Ping, Qiaoli Feng, Yiheng Liang*, et al.* "Maternal Group B Streptococcal Rectovaginal Colonization after Intrapartum Antibiotic Prophylaxis." **9**, no. 12 (2022): 1848

Lusta, M, O Voronkova, O Finkova*, et al.* "Microbiological Monitoring of Antibiotic Resistance of Strains of Streptococcus Agalactiae among Pregnant Women." **14**, no. 2 (2023): 208-12

Madrid, Lola, Sonia Amós Maculuve, Alba Vilajeliu*, et al.* "Maternal Carriage of Group B Streptococcus and Escherichia Coli in a District Hospital in Mozambique." **37**, no. 11 (2018): 1145-53

Makled, Amal F, Ahmed B Mahmoud, Said A Saleh*, et al.* "Rate of Carriage of Streptococcus Agalactiae among Pregnant Women and Role of Some Virulence Genes." **29**, no. 3 (2020): 87-96

Manning, Shannon D, Betsy Foxman, Carl L Pierson*, et al.* "Correlates of Antibiotic-Resistant Group B Streptococcus Isolated from Pregnant Women." **101**, no. 1 (2003): 74-79

Marchaim, D., S. Efrati, R. Melamed*, et al.* "Clonal Variability of Group B Streptococcus among Different Groups of Carriers in Southern Israel." *Eur J Clin Microbiol Infect Dis* **25**, no. 7 (Jul 2006): 443-8.<https://doi.org/10.1007/s10096-006-0163-6>

Marimón, José María, Adoración Valiente, María Ercibengoa*, et al.* "Erythromycin Resistance and Genetic Elements Carrying Macrolide Efflux Genes in Streptococcus Agalactiae." **49**, no. 12 (2005): 5069-74

Matalka, Ala’a, Naser Al-Husban, Oqba Alkuran*, et al.* "Spectrum of Uropathogens and Their Susceptibility to Antimicrobials in Pregnant Women: A Retrospective Analysis of 5-Year Hospital Data." **49**, no. 5 (2021): 03000605211006540

Matani, Chiara, Michele Trezzi, Alice Matteini*, et al.* "Streptococcus Agalactiae: Prevalence of Antimicrobial Resistance in Vaginal and Rectal Swabs in Italian Pregnant Women." **24**, no. 3 (2016): 217-21

Meehan, Mary, Robert Cunney, Mary %J European journal of clinical microbiology Cafferkey*, et al.* "Molecular Epidemiology of Group B Streptococci in Ireland Reveals a Diverse Population with Evidence of Capsular Switching." **33** (2014): 1155-62

MELO, Simone Cristina Castanho Sabaini de, Nathally Claudiane de Souza SANTOS, Marcia de OLIVEIRA*, et al.* "Antimicrobial Susceptibility of Streptococcus Agalactiae Isolated from Pregnant Women." **58** (2016): 83

Mengist, Abeba, Hemalatha Kannan, and Alemseged %J BMC research notes Abdissa. "Prevalence and Antimicrobial Susceptibility Pattern of Anorectal and Vaginal Group B Streptococci Isolates among Pregnant Women in Jimma, Ethiopia." **9** (2016): 1-5

Mengist, Hylemariam Mihiretie, Olifan Zewdie, Adugna Belew*, et al.* "Prevalence and Drug Susceptibility Pattern of Group B Streptococci (Gbs) among Pregnant Women Attending Antenatal Care (Anc) in Nekemte Referral Hospital (Nrh), Nekemte, Ethiopia." **10** (2017): 1-6

Mohamadi, Jasem, Iraj Pakzad, Zahra Khodai*, et al.* "The Prevalence of Vaginal Carrier of Group B Streptococcal (Gbs) among Pregnant Women Attending Maternity at Mustafa-Khomeini Hospital, Ilam." (2017)

Mohamed, Amr Mohamed, Mubashir Ahmad Khan, Aftab Faiz*, et al.* "Group B Streptococcus Colonization, Antibiotic Susceptibility, and Serotype Distribution among Saudi Pregnant Women." **52**, no. 1 (2020): 70

Mohammed, Musa, Daniel Asrat, Yimtubezinash Woldeamanuel*, et al.* "Prevalence of Group B Streptococcus Colonization among Pregnant Women Attending Antenatal Clinic of Hawassa Health Center, Hawassa, Ethiopia." **26**, no. 1 (2012): 36-42

Morales, Walter J, Sonja S Dickey, Patricia Bornick*, et al.* "Change in Antibiotic Resistance of Group B Streptococcus: Impact on Intrapartum Management." **181**, no. 2 (1999): 310-14

Moroi, Hiroaki, Kouji Kimura, Tomomi Kotani*, et al.* "Isolation of Group B Streptococcus with Reduced Β-Lactam Susceptibility from Pregnant Women." **8**, no. 1 (2019): 2-7

Mosca, Adriana, F Russo, and Giuseppe %J Journal of Antimicrobial Chemotherapy Miragliotta. "In Vitro Antimicrobial Activity of Benzalkonium Chloride against Clinical Isolates of Streptococcus Agalactiae." **57**, no. 3 (2006): 566-68

Motlova, J, L Strakova, P Urbaskova*, et al.* "Vaginal & Rectal Carriage of Streptococcus Agalactiae in the Czech Republic: Incidence, Serotypes Distribution & Susceptibility to Antibiotics." **119** (2004): 84-87

Mukesi, Munyaradzi, Benson C Iweriebor, Larry C Obi*, et al.* "The Activity of Commercial Antimicrobials, and Essential Oils and Ethanolic Extracts of Olea Europaea on Streptococcus Agalactiae Isolated from Pregnant Women." **19** (2019): 1-9

Muller, Anouk E, Arijaan W Valkenburg-van den Berg, Deborah Kreft*, et al.* "Low Rate of Carriage of Macrolide-Resistant Group B Streptococci in Pregnant Women in the Netherlands." **137**, no. 1 (2008): 17-20

Nasri, Khadijeh, Ali Chehrei, and Mahdokht Sadat %J Iranian journal of reproductive medicine Manavi. "Evaluation of Vaginal Group B Streptococcal Culture Results after Digital Vaginal Examination and Its Pattern of Antibiotic Resistance in Pregnant Women." **11**, no. 12 (2013): 999

Ngom, Ndeye Safietou, Omar Gassama, Assane Dieng*, et al.* "Vaginal Carriage of Group B Streptococcus (Gbs) in Pregnant Women, Antibiotic Sensitivity and Associated Risk Factors in Dakar, Senegal." **16** (2023): 11786361231174419

Ngonzi, Joseph, Lisa M Bebell, Joel Bazira*, et al.* "Risk Factors for Vaginal Colonization and Relationship between Bacterial Vaginal Colonization and in‐Hospital Outcomes in Women with Obstructed Labor in a Ugandan Regional Referral Hospital." **2018**, no. 1 (2018): 6579139

Njoku, Charles, Cajethan Emechebe, and Anthony %J Int J Women’s Health Reprod Sci Agbakwuru. "Prevalence and Determinants of Anogenital Colonization by Group B Streptococcus Infection among Hiv Positive and Negative Women in Calabar, Nigeria." **6**, no. 1 (2017): 11-7

Nkembe, Nkembe Marius, Hortense Gonsu Kamga, Williams Abange Baiye*, et al.* "Streptococcus Agalactiae Prevalence and Antimicrobial Susceptibility Pattern in Vaginal and Anorectal Swabs of Pregnant Women at a Tertiary Hospital in Cameroon." **11** (2018): 1-6

Numanović, Fatima, Jasmina Smajlović, Merima Gegić*, et al.* "Presence and Resistance of Streptococcus Agalactiae in Vaginal Specimens of Pregnant and Adult Non-Pregnant Women and Association with Other Aerobic Bacteria." **14**, no. 1 (2015): 98-105

Ojo, Oluwole Olutola, DO Awonuga, Iyabode Olabisi Florence Dedeke*, et al.* "Prevalence of Group B Streptococcus Colonisation and Antimicrobial Susceptibility Pattern among Pregnant Women Attending a Tertiary Health Facility in Ogun State, Southwest Nigeria." **9**, no. 3 (2019): 8-14

Orrett, Fitzroy A %J Pediatrics international. "Colonization with Group B Streptococci in Pregnancy and Outcome of Infected Neonates in Trinidad." **45**, no. 3 (2003): 319-23

Otaguiri, Eliane Saori, Ana Elisa Belotto Morguette, Alexandre Tadachi Morey*, et al.* "Development of a Melting-Curve Based Multiplex Real-Time Pcr Assay for Simultaneous Detection of Streptococcus Agalactiae and Genes Encoding Resistance to Macrolides and Lincosamides." **18** (2018): 1-11

Oviedo, P, E Pegels, M Laczeski*, et al.* "Phenotypic and Genotypic Characterization of Streptococcus Agalactiae in Pregnant Women: First Study in a Province of Argentina." **44** (2013): 253-58

Paccione, Kimberly A, Harold C %J Infectious diseases in obstetrics Wiesenfeld, and gynecology. "Guideline Adherence for Intrapartum Group B Streptococci Prophylaxis in Penicillin‐Allergic Patients." **2013**, no. 1 (2013): 917304

Panda, B., I. Iruretagoyena, R. Stiller*, et al.* "Antibiotic Resistance and Penicillin Tolerance in Ano-Vaginal Group B Streptococci." *J Matern Fetal Neonatal Med* **22**, no. 2 (Feb 2009): 111-4.<https://doi.org/10.1080/14767050802488212>

Petca, Aida, Florica Șandru, Silvius Negoiță*, et al.* "Antimicrobial Resistance Profile of Group B Streptococci Colonization in a Sample Population of Pregnant Women from Romania." **12**, no. 2 (2024): 414

Qadi, Mohammad, Adham AbuTaha, Ro’ya Al-Shehab*, et al.* "Prevalence and Risk Factors of Group B Streptococcus Colonization in Pregnant Women: A Pilot Study in Palestine." **2021**, no. 1 (2021): 8686550

Quiroga, M, E Pegels, P Oviedo*, et al.* "Antibiotic Susceptibility Patterns and Prevalence of Group B Streptococcus Isolated from Pregnant Women in Misiones, Argentina." **39** (2008): 245-50

Rojo-Bezares, B., J. M. Azcona-Gutiérrez, C. Martin*, et al.* "Streptococcus Agalactiae from Pregnant Women: Antibiotic and Heavy-Metal Resistance Mechanisms and Molecular Typing." *Epidemiol Infect* **144**, no. 15 (Nov 2016): 3205-14.<https://doi.org/10.1017/s0950268816001692>

Rostami, Soodabeh, Leila Moeineddini, Fereshteh Ghandehari*, et al.* "Macrolide-Resistance, Capsular Genotyping and Associated Factors of Group B Streptococci Colonized Pregnant Women in Isfahan, Iran." **13**, no. 2 (2021): 183

Sadaka, Salama Mohamed, Hala Abdelsalam Aly, Marwa Ahmed Meheissen*, et al.* "Group B Streptococcal Carriage, Antimicrobial Susceptibility, and Virulence Related Genes among Pregnant Women in Alexandria, Egypt." **54**, no. 1 (2018): 69-76

Santana, Fabrícia Almeida Fernandes, Tais Viana Ledo de Oliveira, Marcelo Barreto de Souza Filho*, et al.* "Streptococcus Agalactiae: Identification Methods, Antimicrobial Susceptibility, and Resistance Genes in Pregnant Women." **8**, no. 18 (2020): 3988

Savoia, D., C. Gottimer, C. Crocilla*, et al.* "Streptococcus Agalactiae in Pregnant Women: Phenotypic and Genotypic Characters." *J Infect* **56**, no. 2 (Feb 2008): 120-5.<https://doi.org/10.1016/j.jinf.2007.11.007>

Seo, Yong Soo, Usha Srinivasan, Kwan-Young Oh*, et al.* "Changing Molecular Epidemiology of Group B Streptococcus in Korea." **25**, no. 6 (2010): 817-23

Shabayek, Sarah Ahmed Abd El-kawy, Salah Mohamed Abdalla, Abouzeid MH %J Journal of infection Abouzeid*, et al.* "Vaginal Carriage and Antibiotic Susceptibility Profile of Group B Streptococcus during Late Pregnancy in Ismailia, Egypt." **2**, no. 2 (2009): 86-90

Sherman, Kevin, Sue Whitehead, Edith Blondel-Hill*, et al.* "Penicillin Susceptibility and Macrolide‐Lincosamide‐Streptogramin B Resistance in Group B Streptococcus Isolates from a Canadian Hospital." **23**, no. 4 (2012): 196-98

Shore, Eliane M, Mark H %J Journal of Obstetrics Yudin, and Gynaecology Canada. "Choice of Antibiotic for Group B Streptococcus in Women in Labour Based on Antibiotic Sensitivity Testing." **34**, no. 3 (2012): 230-35

Shrestha, Kusum, Anil Kumar Sah, Neetu Singh*, et al.* "Molecular Characterization of Streptococcus Agalactiae Isolates from Pregnant Women in Kathmandu City." **2020**, no. 1 (2020): 4046703

Simoes, Jose Antonio, Valeria Moraes Neder Alves, Sergio Eduardo Longo Fracalanzza*, et al.* "Phenotypical Characteristics of Group B Streptococcus in Parturients." **11** (2007): 261-66

Slotved, Hans-Christian, Jens Kjølseth Møller, Mohammad Rohi Khalil*, et al.* "The Serotype Distribution of Streptococcus Agalactiae (Gbs) Carriage Isolates among Pregnant Women Having Risk Factors for Early-Onset Gbs Disease: A Comparative Study with Gbs Causing Invasive Infections during the Same Period in Denmark." **21** (2021): 1-8

Soares, Georgia Cristina Tavolaro, Daniela Sales Alviano, Gabriela da Silva Santos*, et al.* "Prevalence of Group B Streptococcus Serotypes Iii and V in Pregnant Women of Rio De Janeiro, Brazil." **44** (2013): 869-72

Souza, Viviane C, Fabíola CO Kegele, Selma R Souza*, et al.* "Antimicrobial Susceptibility and Genetic Diversity of Streptococcus Agalactiae Recovered from Newborns and Pregnant Women in Brazil." **45**, no. 10 (2013): 780-85

Spaetgens, Renee, Kim DeBella, Doreen Ma*, et al.* "Perinatal Antibiotic Usage and Changes in Colonization and Resistance Rates of Group B Streptococcus and Other Pathogens." **100**, no. 3 (2002): 525-33

Strus, Magdalena, Dorota Pawlik, Monika Brzychczy-Włoch*, et al.* "Group B Streptococcus Colonization of Pregnant Women and Their Children Observed on Obstetric and Neonatal Wards of the University Hospital in Krakow, Poland." **58**, no. 2 (2009): 228-33

Stylianopoulos, Anastasia, Nigel Kelly, Suzanne %J Australian Garland*, et al.* "Is Penicillin and/or Erythromycin Resistance Present in Clinical Isolates of Group B Streptococcus in Our Community?". **42**, no. 5 (2002): 543-44

Teatero, Sarah, Patricia Ferrieri, Irene Martin*, et al.* "Serotype Distribution, Population Structure, and Antimicrobial Resistance of Group B Streptococcus Strains Recovered from Colonized Pregnant Women." **55**, no. 2 (2017): 412-22

Tesfaye, Addisu, Addisu Melese, and Awoke %J International Journal of Microbiology Derbie. "Antimicrobial Resistance Profile and Associated Factors of Group B Streptococci Colonization among Pregnant Women Attending Antenatal Clinics in Jigjiga, Southeast Ethiopia." **2022**, no. 1 (2022): 9910842

Tor-Udom, Siripen, Pharuhat Tor-Udom, and Wanwarang %J JOURNAL-MEDICAL ASSOCIATION OF THAILAND Hiriote. "The Prevalence of Streptococcus Agalactiae (Group B) Colonization in Pregnant Women at Thammasat Hospital." **89**, no. 4 (2006): 411

Tsai, Ming-Horng, Jen-Fu Hsu, Mei-Yin Lai*, et al.* "Molecular Characteristics and Antimicrobial Resistance of Group B Streptococcus Strains Causing Invasive Disease in Neonates and Adults." **10** (2019): 264

Tsolia, M, M Psoma, S Gavrili*, et al.* "Group B Streptococcus Colonization of Greek Pregnant Women and Neonates: Prevalence, Risk Factors and Serotypes." **9**, no. 8 (2003): 832-38

Vinnemeier, C. D., P. Brust, E. Owusu-Dabo*, et al.* "Group B Streptococci Serotype Distribution in Pregnant Women in Ghana: Assessment of Potential Coverage through Future Vaccines." *Trop Med Int Health* **20**, no. 11 (Nov 2015): 1516-24.<https://doi.org/10.1111/tmi.12589>

Wang, Ping, Jing-jing Tong, Xiu-hua Ma*, et al.* "Serotypes, Antibiotic Susceptibilities, and Multi-Locus Sequence Type Profiles of Streptococcus Agalactiae Isolates Circulating in Beijing, China." **10**, no. 3 (2015): e0120035

Warrier, Lakshmi M, Sapna Joy, and Rani Ameena %J Indian Journal of Pediatrics Bashir. "Group B Streptococcal Colonization among Pregnant Women and Neonates in a Tertiary Care Hospital in South India." **89**, no. 12 (2022): 1187-94

Woldu, Zufan Lakew, Tatek Gebreegziabher Teklehaimanot, Sisay Teklu Waji*, et al.* "The Prevalence of Group B Streptococus Recto-Vaginal Colonization and Antimicrobial Susceptibility Pattern in Pregnant Mothers at Two Hospitals of Addis Ababa, Ethiopia." **11** (2014): 1-4

Yan, Yuzhong, Hua Hu, Tingyan Lu*, et al.* "Investigation of Serotype Distribution and Resistance Genes Profile in Group B Streptococcus Isolated from Pregnant Women: A Chinese Multicenter Cohort Study." **124**, no. 9 (2016): 794-99

Yeung, Sik Wing, Daljit Singh Sahota, Tak Yeung %J Taiwanese Journal of Obstetrics Leung*, et al.* "Comparison of the Effect of Penicillins Versus Erythromycin in Preventing Neonatal Group B Streptococcus Infection in Active Carriers Following Preterm Prelabor Rupture of Membranes." **53**, no. 2 (2014): 210-14

Yılmaz Karadağ, Fatma, Kenan Hızel, and Orhan Gelişen. "Colonization of Group B Streptococci in Pregnant Women at Delivery." (2013)

Zhang, Hong, Wen-Li Liu, Jia-Lin Liu*, et al.* "Identification and Characteristics of Antimicrobial-Resistant Group B Streptococcus Isolated from Maternal Birth Canals at a University-Affiliated Hospital, China." **48**, no. 2 (2021): 253-56

Zhou, Jie, Li Zhang, Yang Zhang*, et al.* "Analysis of Molecular Characteristics of Camp-Negative Streptococcus Agalactiae Strains." **14** (2023): 1189093
