# Supplementary material for: Global Antimicrobial Resistance Trends in Group B Streptococcus Isolates From Pregnant Women: Systematic Review and Meta‐Analysis
Source: Microbiologyopen. 2025 Oct 30;14(6):e70087. doi: 10.1002/mbo3.70087 (PMC12575007; doi:10.1002/mbo3.70087)
Supplement: Supplementary file 3 — Supporting File 3: Results of Subgroup Analyses. [file MBO3-14-e70087-s005.docx]

**Figure 1: The forest plots for Penicillin resistant *S. agalactiae* isolates**

**Figure 2: The forest plots for Ampicillin resistant *S. agalactiae* isolates**

**Figure 3: The forest plots for Ceftriaxone resistant *S. agalactiae* isolates**

**Figure 4: The forest plots for Cefotaxime resistant *S. agalactiae* isolates**

**Figure 5: The forest plots for Cefepime resistant *S. agalactiae* isolates**

**Figure 6: The forest plots for Clindamycin resistant *S. agalactiae* isolates**

**Figure 7: The forest plots for Erythromycin resistant *S. agalactiae* isolates**

**Figure 8: The forest plots for Vancomycin resistant *S. agalactiae* isolates**

**
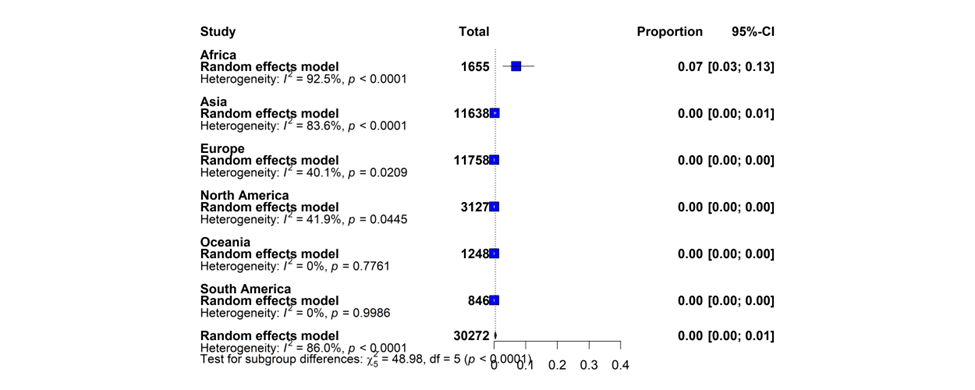
**

Figure 1a. Sub group analysis for continents of Penicillin resistant *S. agalactiae* isolates.


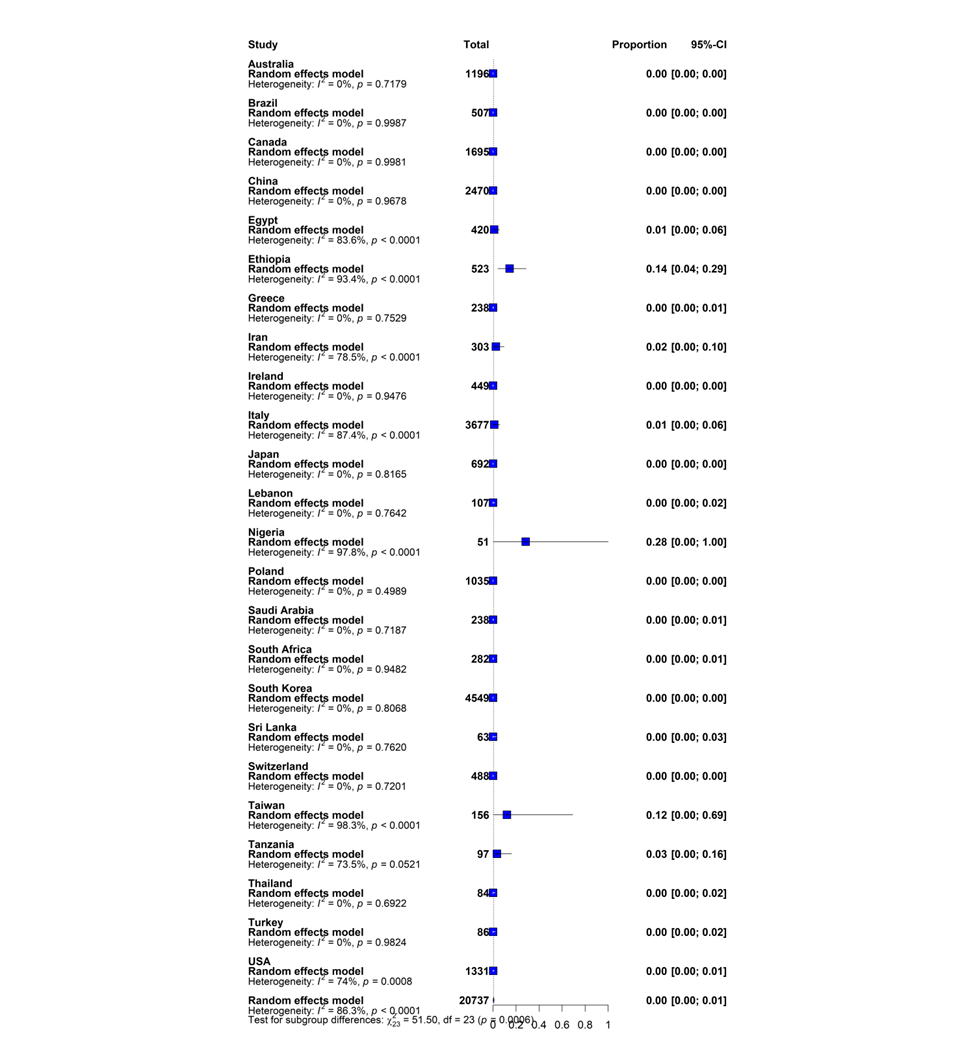
Figure 1b. Sub group analysis for country of Penicillin resistant S. agalactiae isolates.


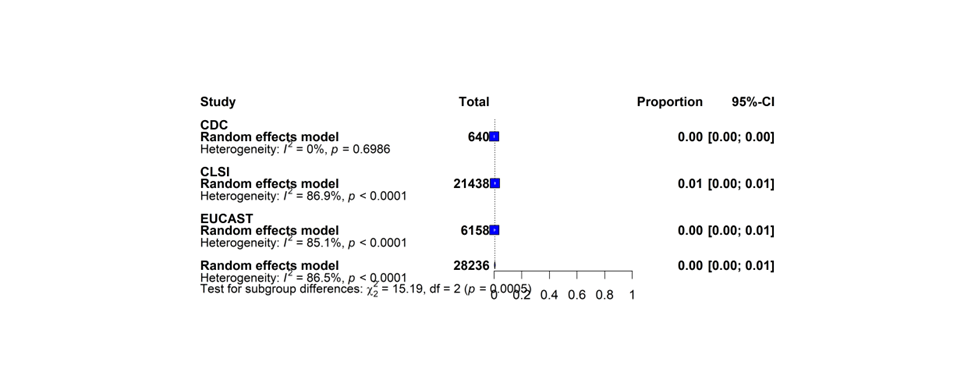
Figure 1c. Sub group analysis for guideline of Penicillin resistant S. agalactiae isolates.


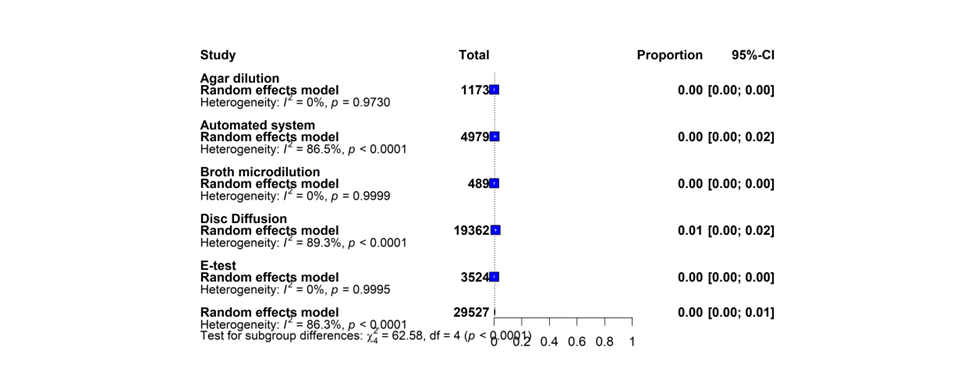


Figure 1d. Sub group analysis for AST method of Penicillin resistant S. agalactiae isolates.


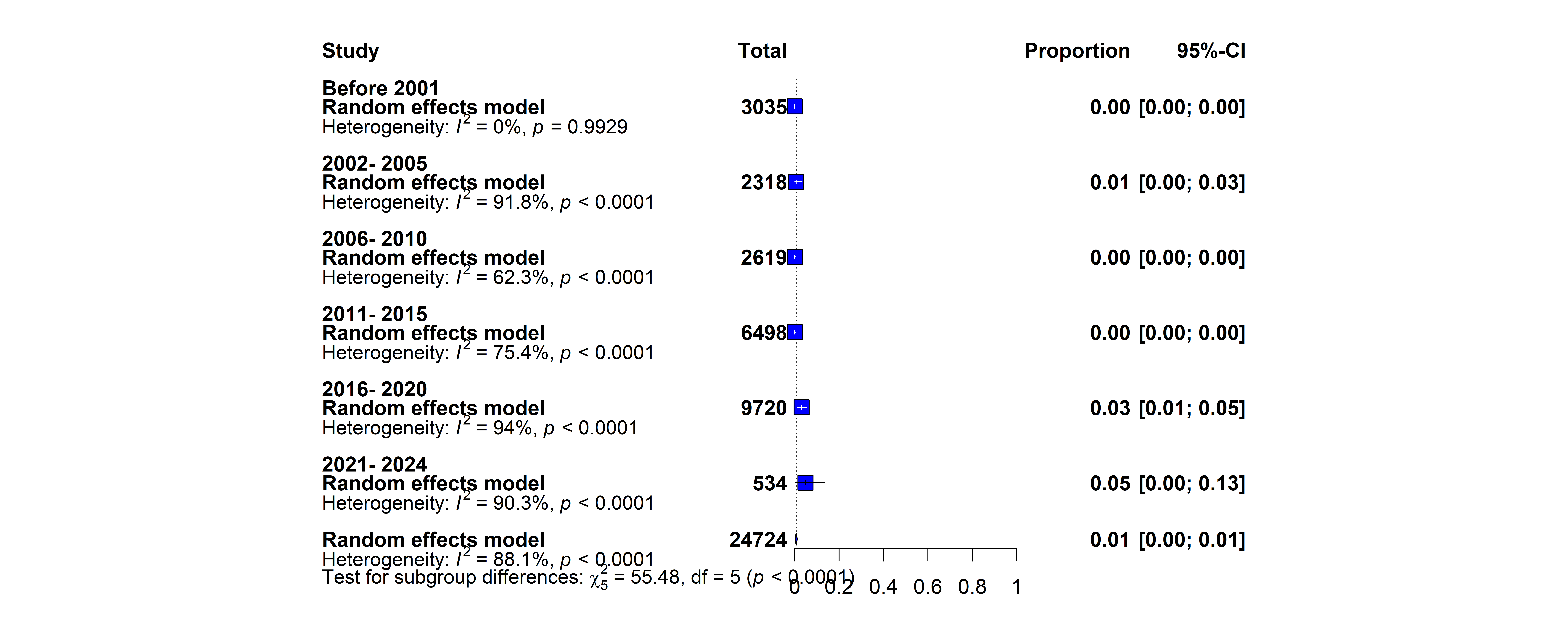


Figure 1e. Sub group analysis for period time time of isolation of Penicillin resistant S. agalactiae isolates.

**
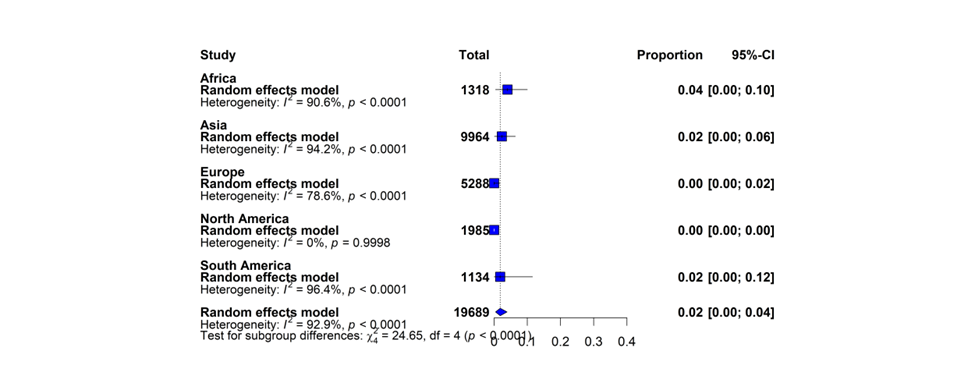
**

Figure 2a. Sub group analysis for continents of Ampicillin resistant S. agalactiae isolates.



 Figure 2b. Sub group analysis for country of Ampicillin resistant S. agalactiae isolates.

Figure 2c. Sub group analysis for AST method of Ampicillin resistant S. agalactiae isolates.
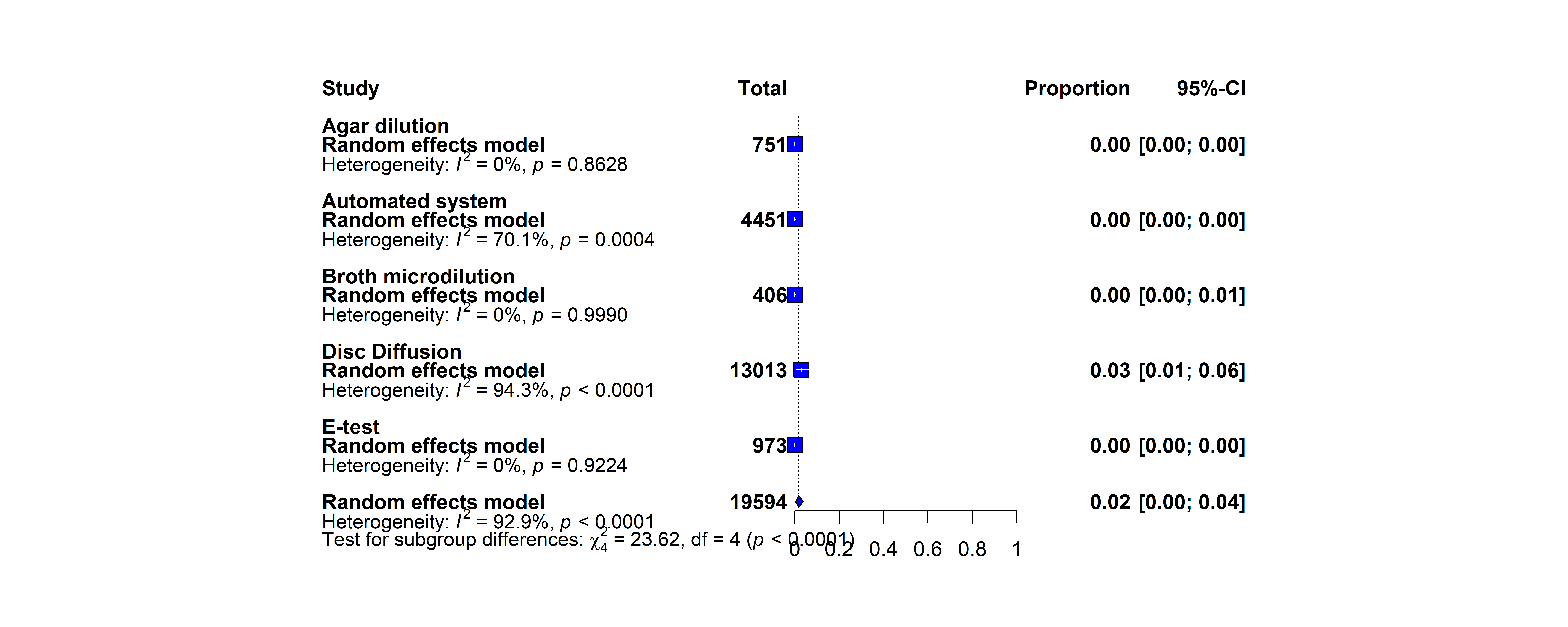


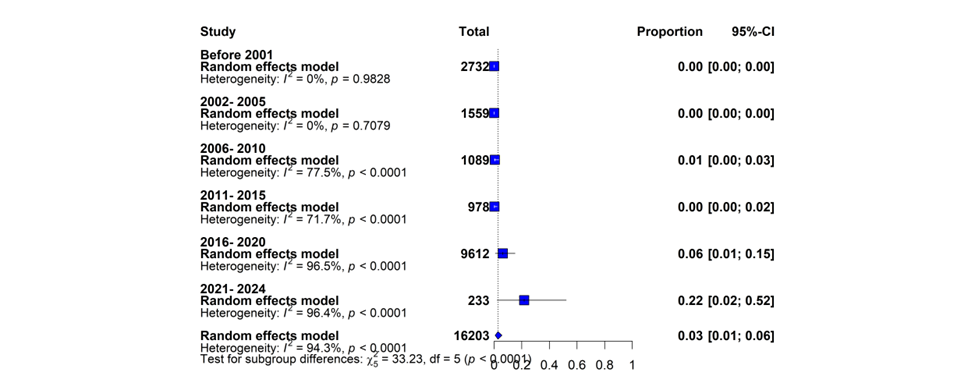


Figure 2c. Sub group analysis for period time of isolation of Ampicillin resistant S. agalactiae isolates.

Figure 3a. Sub group analysis for continents of Ceftriaxone resistant S. agalactiae isolates.
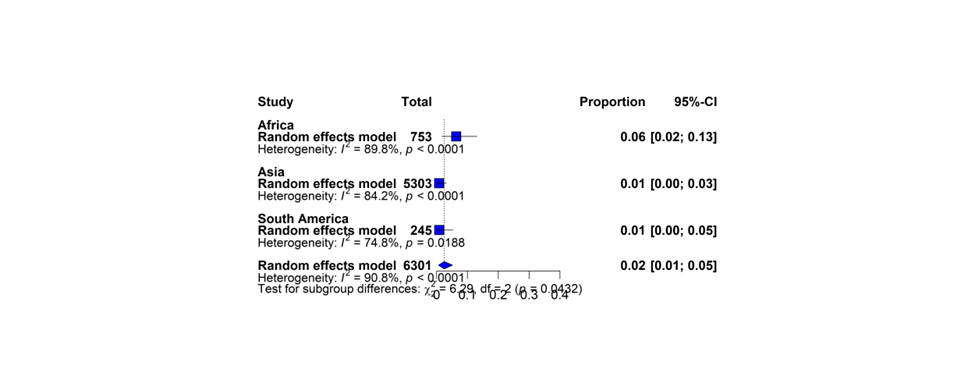


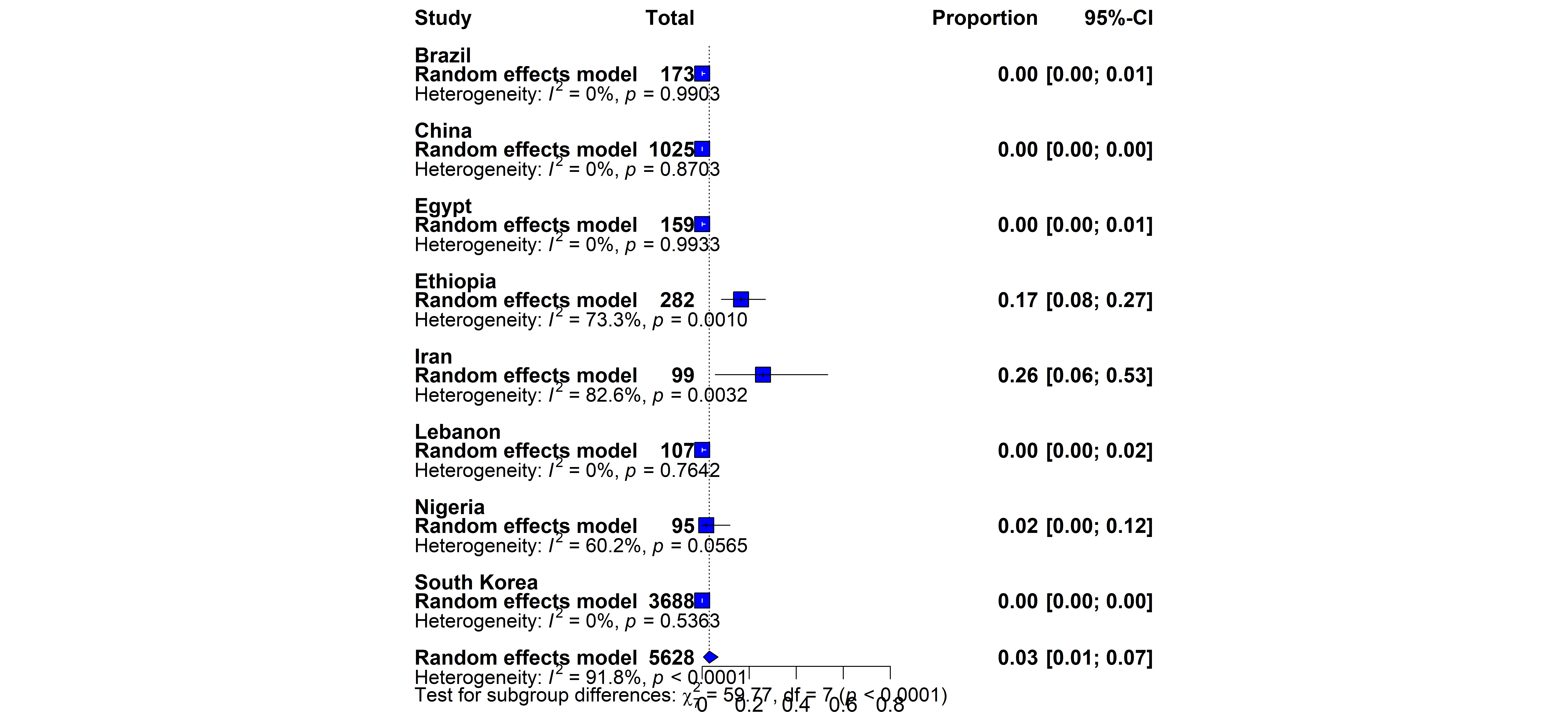


Figure 3b. Sub group analysis for country of Ceftriaxone resistant S. agalactiae isolates.


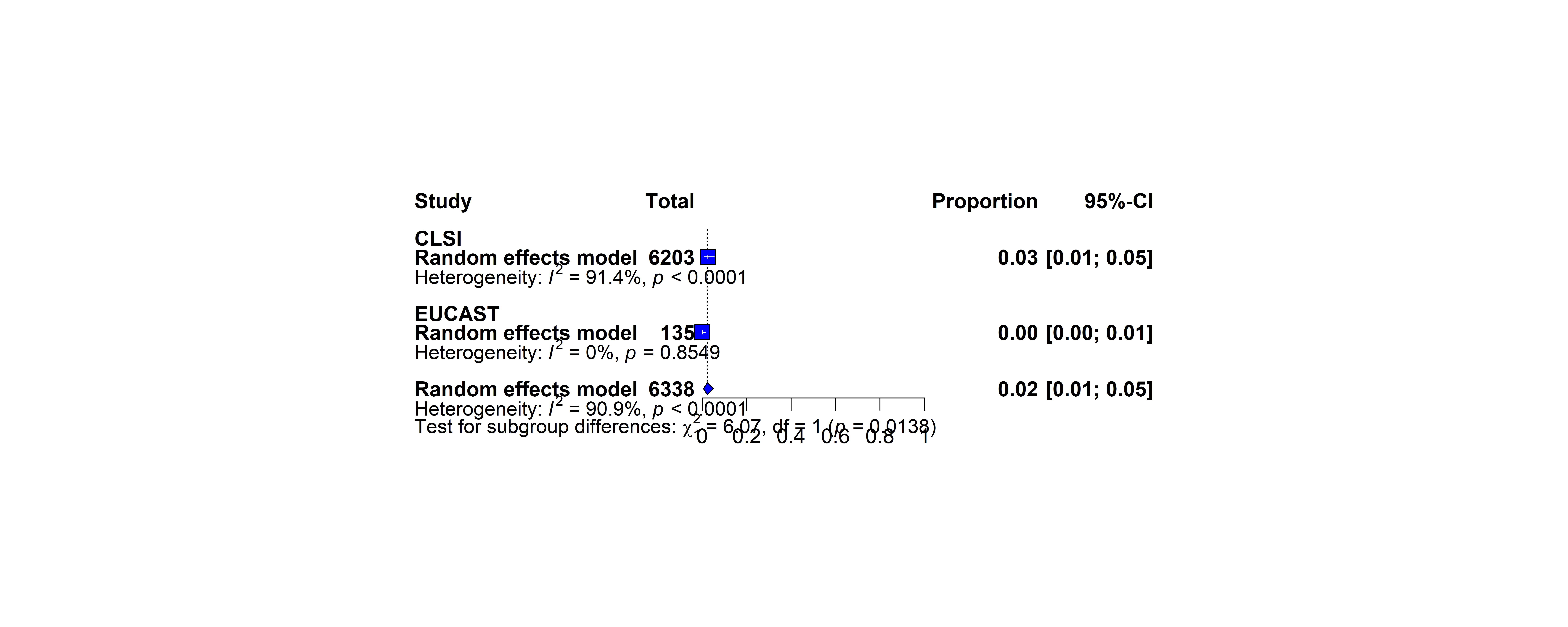
 Figure 3c. Sub group analysis for guideline of Ceftriaxone resistant S. agalactiae isolates.


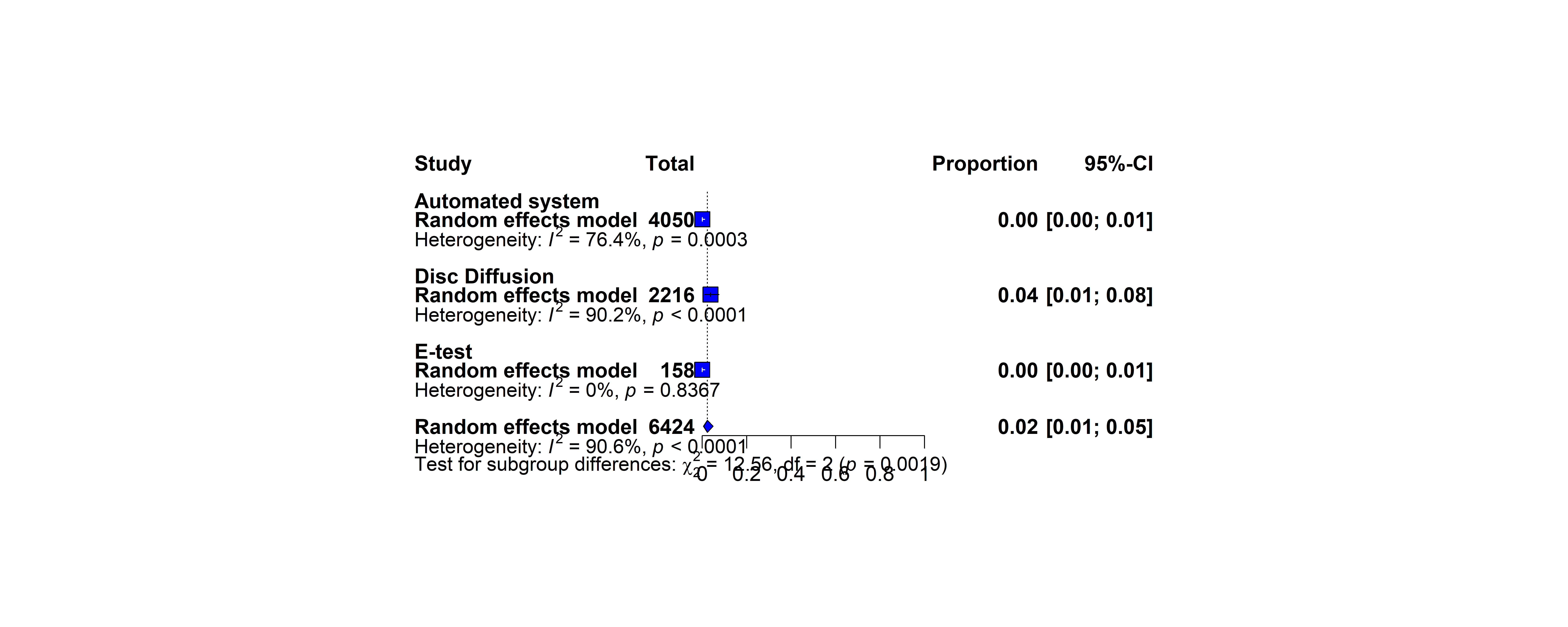


Figure 3d. Sub group analysis for AST method of Ceftriaxone resistant S. agalactiae isolates.

Figure 4a. Sub group analysis for continents of Cefotaxime resistant S. agalactiae isolates.
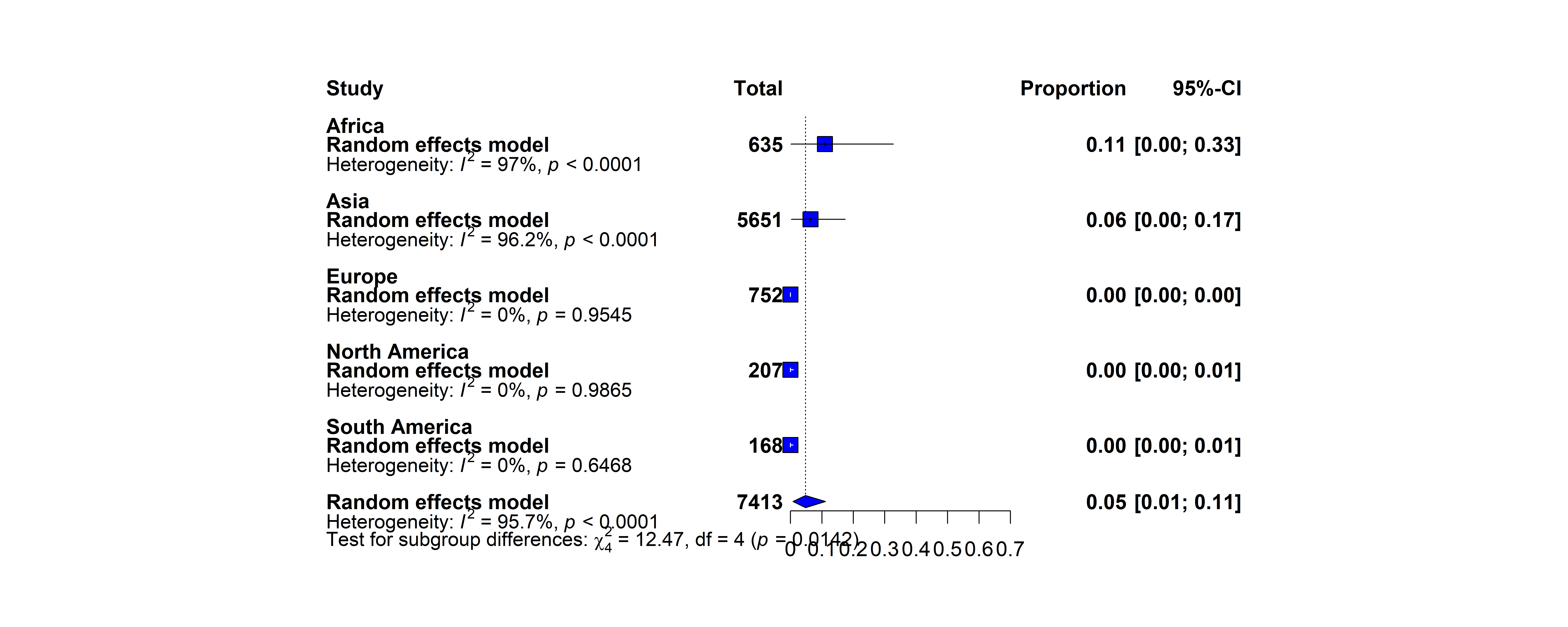





Figure 4b. Sub group analysis for country of Cefotaxime resistant S. agalactiae isolates.

Figure 4c. Sub group analysis for AST method of Cefotaxime resistant S. agalactiae isolates.
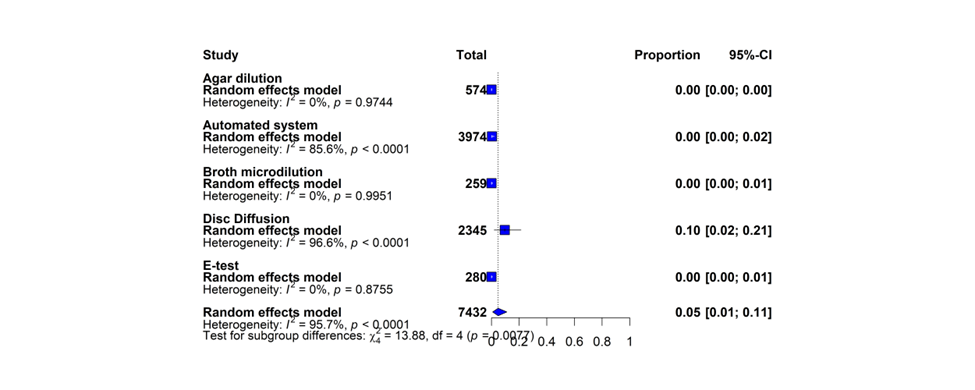


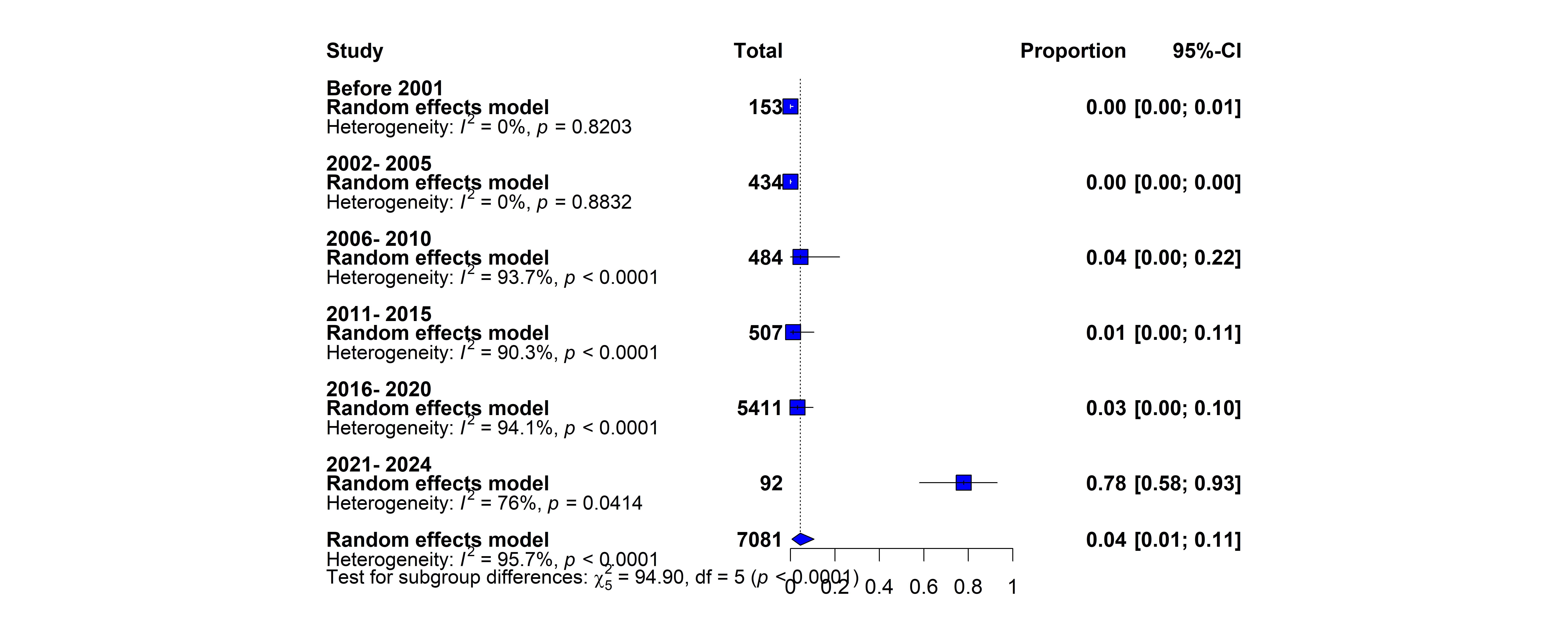


Figure 4d. Sub group analysis for period time of isolation of Cefotaxime resistant S. agalactiae isolates.


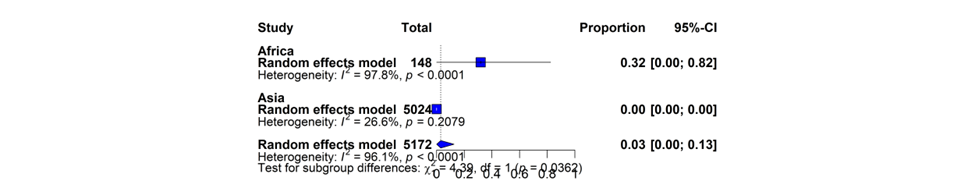


Figure 5a. Sub group analysis for continents of Cefepime resistant S. agalactiae isolates.





Figure 5b. Sub group analysis for country of Cefepime resistant S. agalactiae isolates.


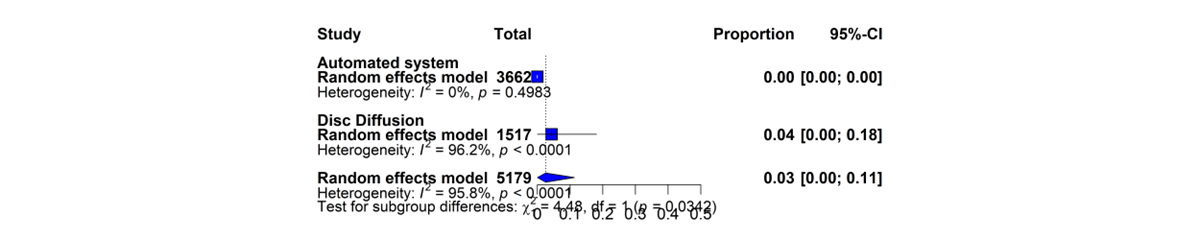


Figure 5c. Sub group analysis for AST method of Cefepime resistant S. agalactiae isolates.

Figure 6a. Sub group analysis for continents of Clindamycin resistant S. agalactiae isolates.
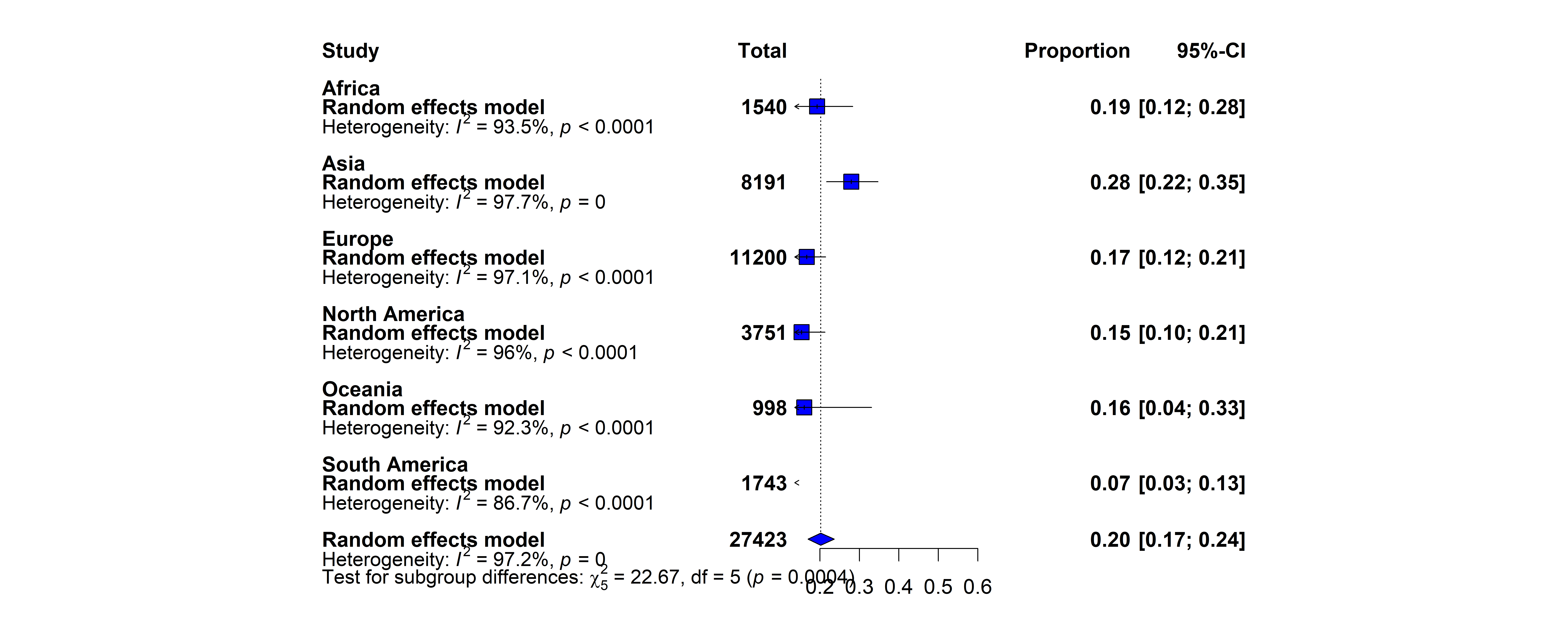





Figure 6b. Sub group analysis for countries of Clindamycin resistant S. agalactiae isolates.


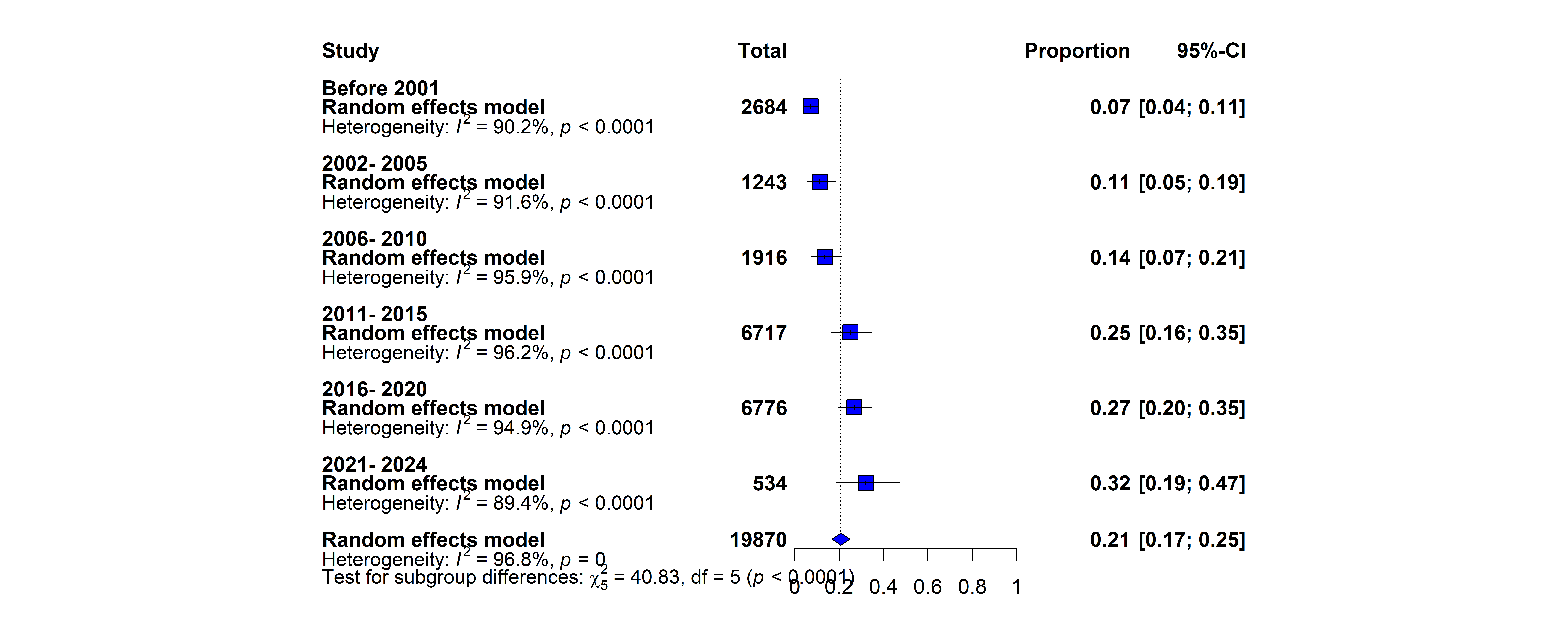


Figure 6c. Sub group analysis for period time of isolation of Clindamycin resistant S. agalactiae isolates.


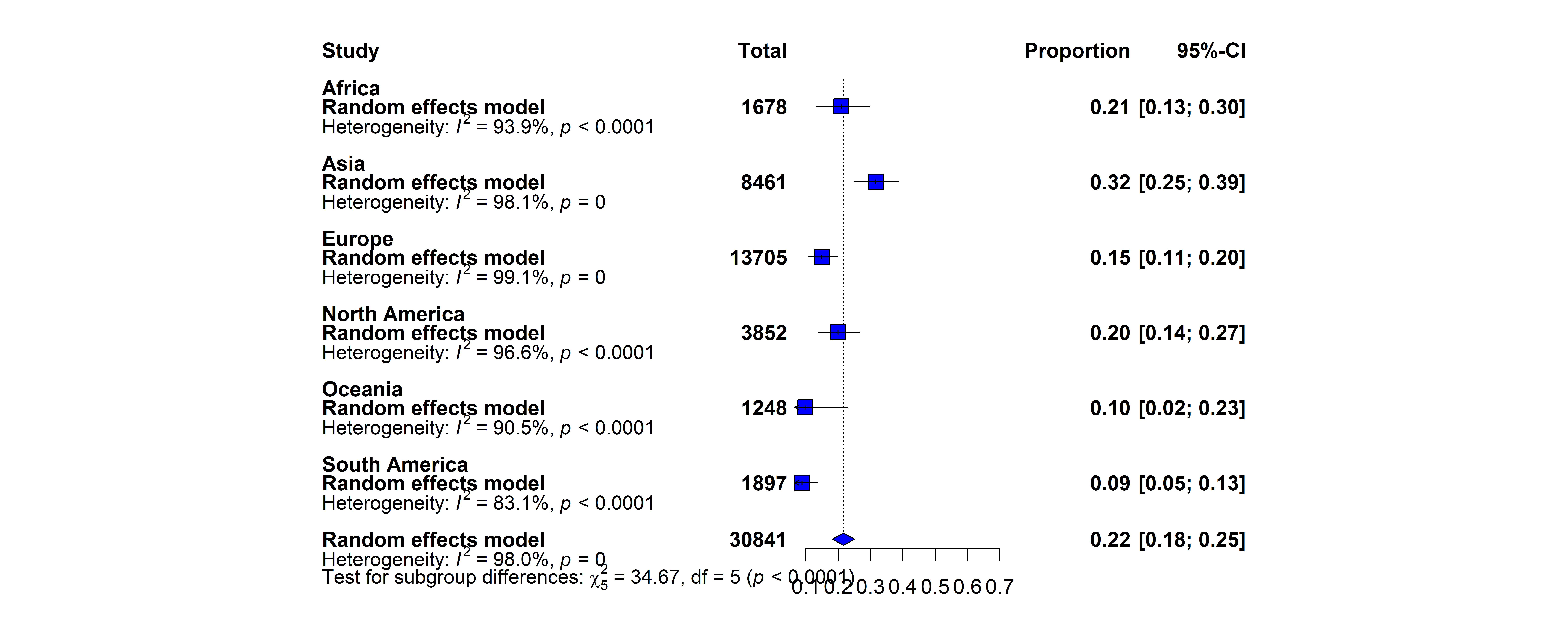


Figure 7a. Sub group analysis for continents of Erythromycin resistant S. agalactiae isolates.





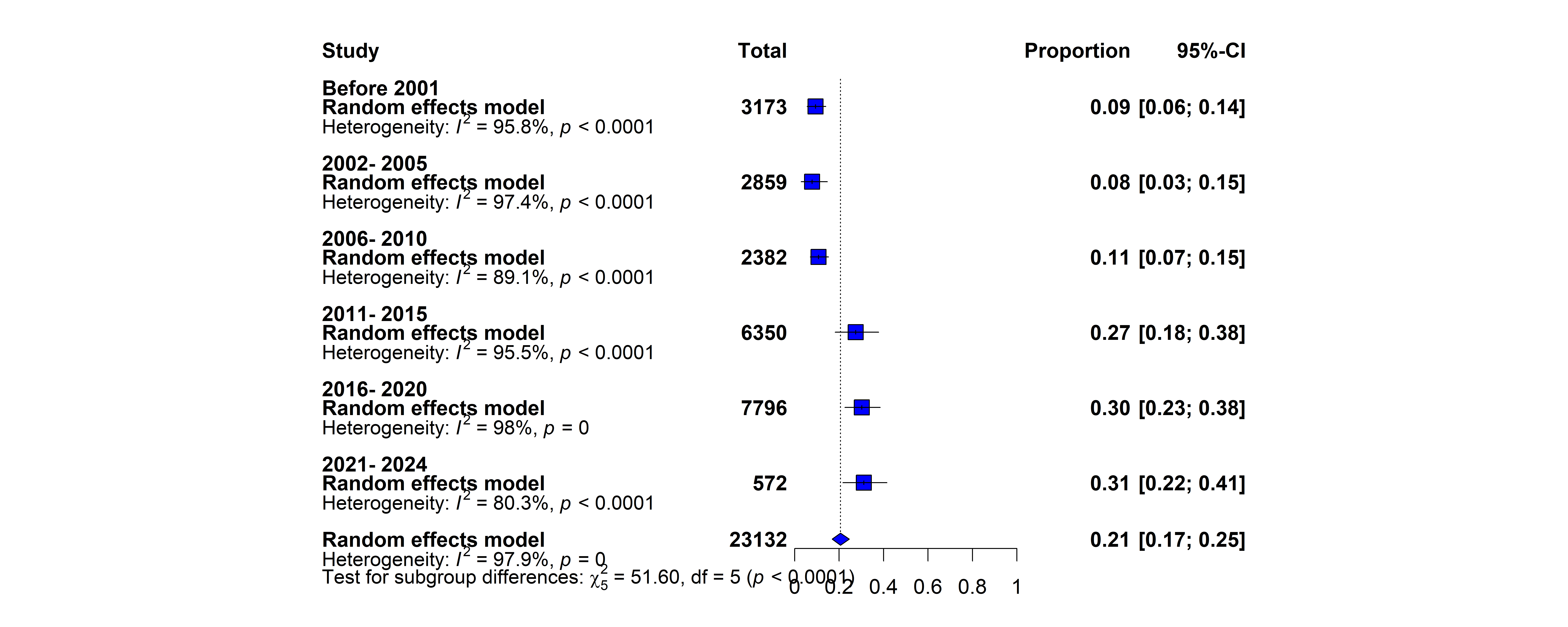
Figure 7b. Sub group analysis for countries of Erythromycin resistant S. agalactiae isolates.

Figure 7c. Sub group analysis for period time of isolation of Erythromycin resistant S. agalactiae isolates.

Figure 8a. Sub group analysis for continents of Vancomycin resistant S. agalactiae isolates.
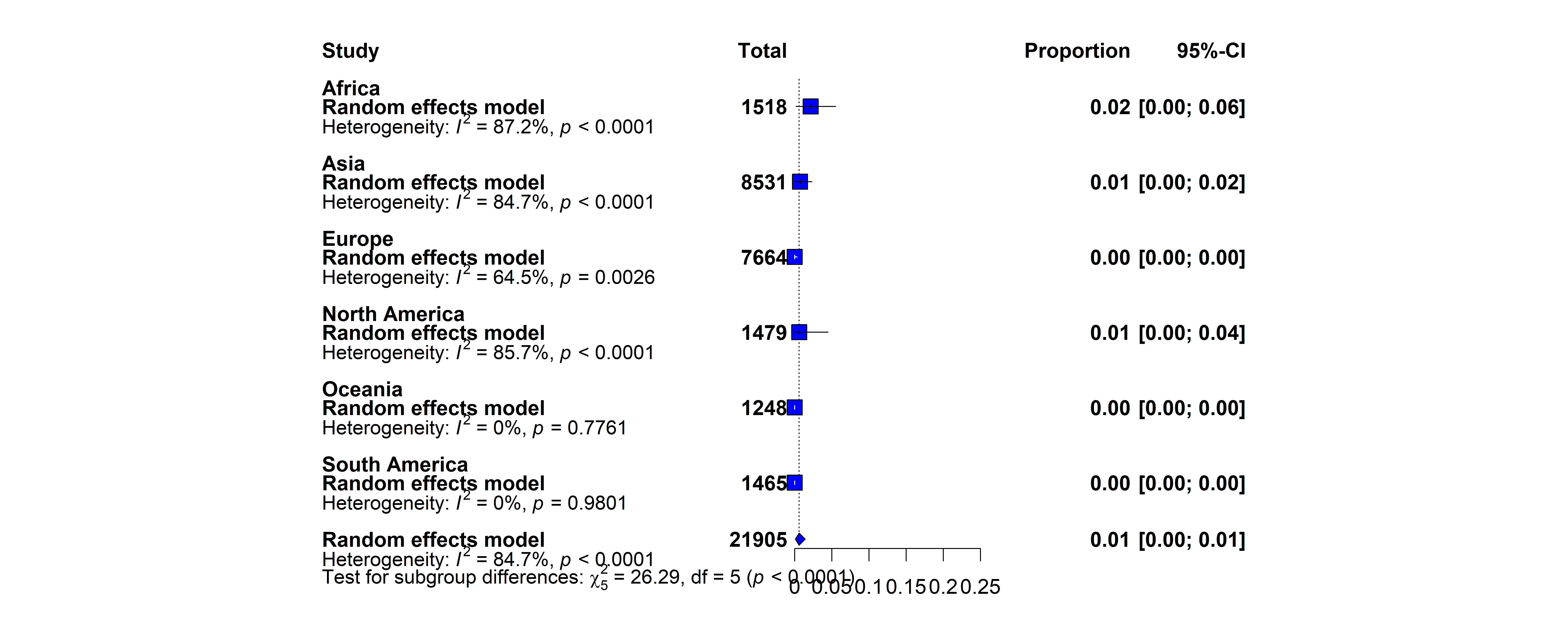





Figure 8b. Sub group analysis for countries of Vancomycin resistant S. agalactiae isolates.


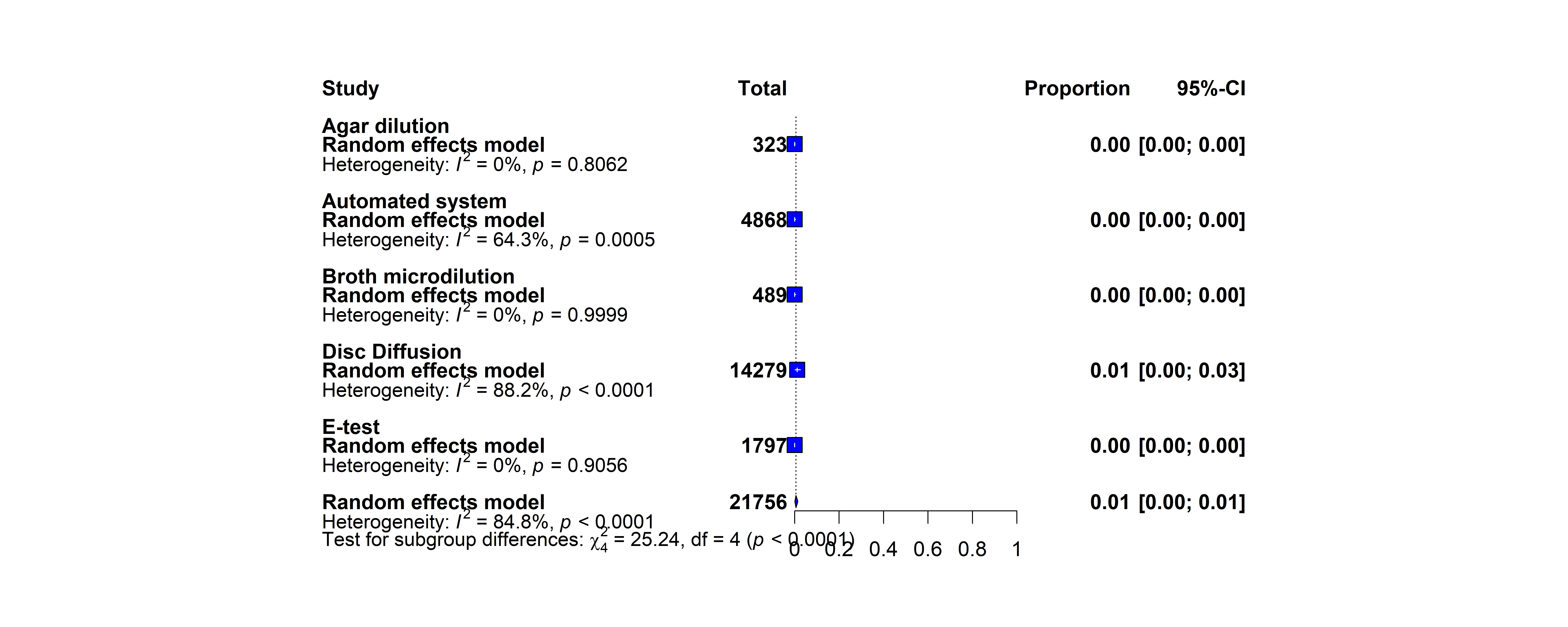


Figure 8c. Sub group analysis for AST method of Vancomycin resistant S. agalactiae isolates.


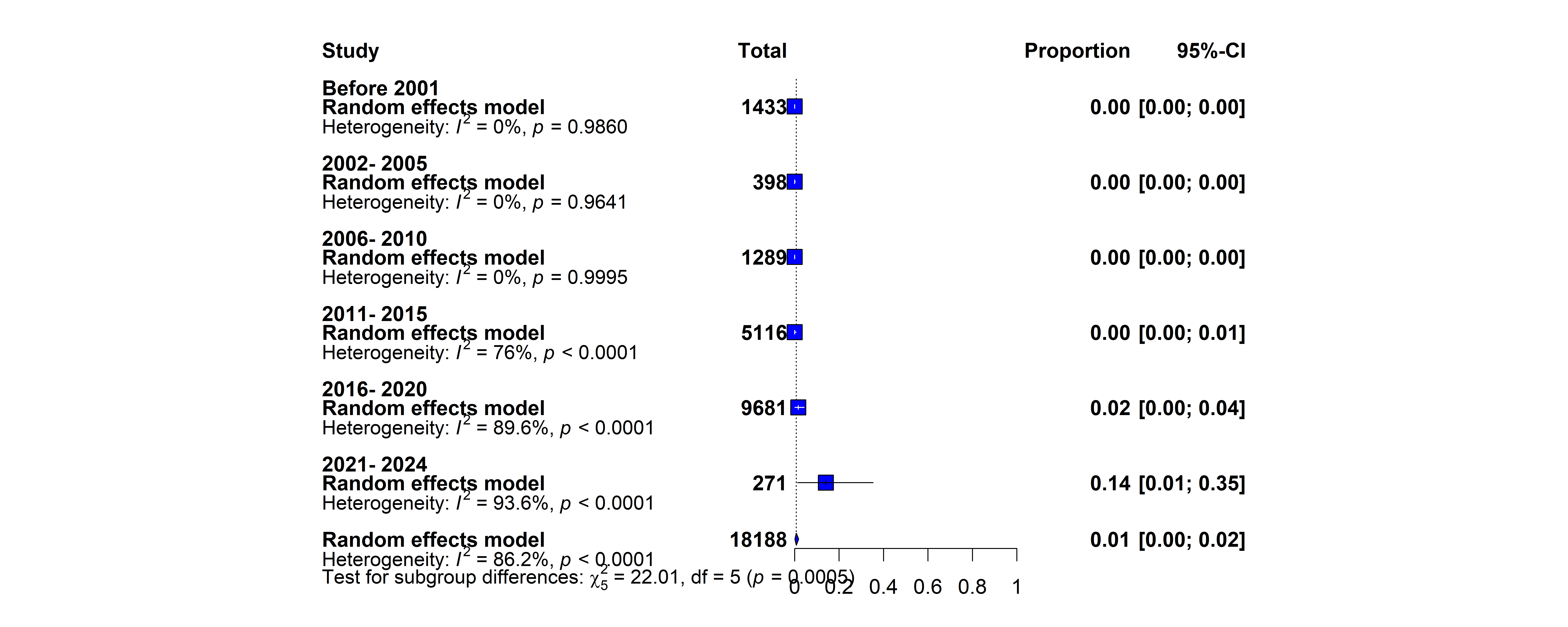


Figure 8d. Sub group analysis for period time of isolation of Vancomycin resistant S. agalactiae isolates.
