# Supplementary material for: Global Antimicrobial Resistance Trends in Group B Streptococcus Isolates From Pregnant Women: Systematic Review and Meta‐Analysis
Source: Microbiologyopen. 2025 Oct 30;14(6):e70087. doi: 10.1002/mbo3.70087 (PMC12575007; doi:10.1002/mbo3.70087)
Supplement: Supplementary file 5 — Supporting Table S2: Detailed results of meta‐analysis and subgroup analyzing. [file MBO3-14-e70087-s003.docx]

Supplementary Table S2: Detailed results of meta-analysis and subgroup analyzing

| Category | Subgroup | K_n_N | Proportion | 95% CI | I² (%) |
| --- | --- | --- | --- | --- | --- |
| Clindamycin |  |  |  |  |  |
| Overall | ND | 140 (6298, 27423) | 0.2020 | [0.1697; 0.2362] | 97.2% |
| Country | Iran | 7 (266, 65) | 0.2739 | [0.0312; 0.6145] | 95.4% |
|  | Bosnia and Herzegovina | 1 (7,2) | 0.2857 | [0.0098; 0.6822] | -- |
|  | Turkey | 3 (66, 10) | 0.1444 | [0.0556; 0.2589] | 0.7% |
|  | France | 1 (2,10) | 0.2000 | [0.0054; 0.5132] | -- |
|  | Brazil | 10 (71, 1294) | 0.0938 | [0.0350; 0.1732] | 87.0% |
|  | Algeria | 1 (8, 15) | 0.5333 | [0.2757; 0.7828] | -- |
|  | China | 13 (1223, 2964) | 0.4768 | [0.3758; 0.5788] | 97.9% |
|  | Ohangwena | 1 (0, 18) | 0.0000 | [0.0000; 0.0934] | -- |
|  | Sri Lanka | 2 (11, 63) | 0.1445 | [0.0231; 0.3290] | 59.7% |
|  | Thailand | 2 (10, 84) | 0.1739 | [0.0000; 0.6653] | 93.7% |
|  | Ethiopia | 11(94, 497) | 0.1812 | [0.1165; 0.2556] | 72.7% |
|  | Yemen | 1 (2, 23) | 0.0870 | [0.0017; 0.2446] | -- |
|  | Egypt | 8 (101, 350) | 0.2782 | [0.1395; 0.4412] | 91.0% |
|  | Nepal | 1 (10, 24) | 0.4167 | [0.2251; 0.6212] | -- |
|  | Palestine | 1 (6, 24) | 0.2500 | [0.0938; 0.4453] | -- |
|  | South Korea | 4 (265, 649) | 0.2796 | [0.1171; 0.4780] | 95.4% |
|  | Tanzania | 2 (19, 97) | 0.1933 | [0.1186; 0.2799] | 0.0% |
|  | Lebanon | 2 (13, 107) | 0.1199 | [0.0628; 0.1907] | 0.0% |
|  | Nigeria | 1 (8, 33) | 0.2424 | [0.1091; 0.4051] | -- |
|  | Vietnam | 1 (21, 33) | 0.6364 | [0.4637; 0.7935] | -- |
|  | USA | 9 (409, 2164) | 0.1407 | [0.0725; 0.2262] | 97.2% |
|  | India | 1 (8, 40) | 0.2000 | [0.0884; 0.3400] | -- |
|  | Senegal | 1 (11, 43) | 0.2558 | [0.1350; 0.3981] | -- |
|  | South Africa | 3 (107, 331) | 0.5252 | [0.0136; 0.9970] | 98.9% |
|  | Australia | 2 (76, 946) | 0.1681 | [0.0040; 0.4766] | 95.5% |
|  | Saudi Arabia | 3 (21, 288) | 0.0779 | [0.0290; 0.1453] | 63.3% |
|  | Italy | 5 (1193, 4003) | 0.2529 | [0.1618; 0.3562] | 92.3% |
|  | New Zealand | 1 (8, 52) | 0.1538 | [0.0667; 0.2663] | -- |
|  | Lisbon | 1 (1, 54) | 0.0185 | [0.0000; 0.0778] | -- |
|  | Taiwan | 2 (62, 156) | 0.3656 | [0.1610; 0.5986] | 87.7% |
|  | Africa | 1 (1, 57) | 0.0175 | [0.0000; 0.0738] | -- |
|  | Malaysia | 1 (11, 60) | 0.1833 | [0.0943; 0.2924] | -- |
|  | Poland | 3 (279, 1415) | 0.1953 | [0.1748; 0.2166] | 54.9% |
|  | Greece | 2 (40, 238) | 0.1300 | [0.0207; 0.3064] | 89.2% |
|  | Mozambique | 1 (20, 68) | 0.2941 | [0.1911; 0.4086] | -- |
|  | Trinidad | 1 (7, 72) | 0.0972 | [0.0378; 0.1780] | -- |
|  | Japan | 3 (90, 692 | 0.1424 | [0.0340; 0.3052] | 91.8% |
|  | Ireland | 2 (50, 278) | 0.1793 | [0.1359; 0.2271] | 0.0% |
|  | Argentina | 2 (4, 377) | 0.0105 | [0.0000; 0.0330] | 39.9% |
|  | Denmark | 1 (25, 96) | 0.2604 | [0.1770; 0.3533] | -- |
|  | Ghana | 1 (3, 97) | 0.0309 | [0.0039; 0.0769] | -- |
|  | Canada | 7 (284, 1587) | 0.1702 | [0.0956; 0.2605] | 93.6% |
|  | Gabon | 1 (0, 180) | 0.0000 | [0.0000; 0.0157] | -- |
|  | Switzerland | 2 (65, 488) | 0.1684 | [0.0235; 0.4032] | 96.3% |
|  | Ukraine | 1 (41, 149) | 0.2752 | [0.2062; 0.3499] | -- |
|  | Romania | 1 (36, 152) | 0.2368 | [0.1724; 0.3079] | -- |
|  | Emirates | 1 (91, 158) | 0.5759 | [0.4979; 0.6522] | -- |
|  | Czech Republic | 1 (5, 172) | 0.0291 | [0.0082; 0.0605] | -- |
|  | Malasiya | 1 (5, 200) | 0.0250 | [0.0071; 0.0521] | -- |
|  | Belgium | 1 (29, 262) | 0.1107 | [0.0753; 0.1518] | -- |
|  | Netherland | 1 (6, 365) | 0.0164 | [0.0055; 0.0325] | -- |
|  | Spain | 1 (52, 375) | 0.1387 | [0.1054; 0.1756] | -- |
|  | UK | 1 (68, 535) | 0.1271 | [0.1001; 0.1567] | -- |
|  | Kuwait | 1 (20, 1166) | 0.0172 | [0.0104; 0.0255] | -- |
|  | Hungary | 1 (1239, 3554) | 0.3461 | [0.3305; 0.3618] | -- |
| Continent | Asia | 56 (2209, 8191) | 0.2796 | [0.2165; 0.3470] | 97.7% |
|  | Europe | 25 (2933, 11200) | 0.1666 | [0.1234; 0.2144] | 97.1% |
|  | South America | 13 (82, 1743) | 0.0745 | [0.0302; 0.1335] | 86.7% |
|  | Africa | 27 (297, 1540) | 0.1929 | [0.1159; 0.2830] | 93.5% |
|  | North America | 16 (693, 3751) | 0.1534 | [0.1020; 0.2128] | 96.0% |
|  | Oceania | 3 (84, 998) | 0.1601 | [0.0419; 0.3308] | 92.3% |
| Time | 2011-2015 | 26 (1917, 6717) | 0.2511 | [0.1641; 0.3487] | 96.2% |
|  | 2016-2020 | 36 (1934, 6776) | 0.2683 | [0.1951; 0.3481] | 94.9% |
|  | 2006-2010 | 16 (402, 1916) | 0.1356 | [0.0718; 0.2143] | 95.9% |
|  | 2002-2005 | 9 (115, 1243) | 0.1122 | [0.0542; 0.1861] | 91.6% |
|  | 2021-2024 | 7 (164, 534) | 0.3207 | [0.1870; 0.4708] | 89.4% |
|  | Before 2001 | 10 (144, 2684) | 0.0721 | [0.0420; 0.1090] | 90.2% |
| Amoxicillin |  |  | 0.0391 | [0.0000; 0.3471] | 85.7% |
| Overall | ND | 4 (4, 383) |  |  |  |
| Country | Iran | 7 (73, 256) | 0.1954 | [0.0186; 0.4624] | 96.3% |
|  | Nigeria | 3 (20, 57) | 0.4711 | [0.0000; 1.0000] | 96.7% |
|  | Bosnia and Herzegovina | 1 (0, 7) | 0.0000 | [0.0000; 0.2319] | -- |
|  | Turkey | 2 (0, 42) | 0.0000 | [0.0000; 0.0357] | 0.0% |
|  | France | 1 (0, 10) | 0.0000 | [0.0000; 0.1652] | -- |
|  | Cameroon | 1 (0, 14) | 0.0000 | [0.0000; 0.1193] | -- |
|  | China | 6 (3, 1538) | 0.0000 | [0.0000; 0.0006] | 0.0% |
|  | Ohangwena | 1 (0, 18) | 0.0000 | [0.0000; 0.0934] | -- |
|  | Sri Lanka | 2 (0, 63) | 0.0000 | [0.0000; 0.0271] | 0.0% |
|  | Ethiopia | 11 (59, 440) | 0.0780 | [0.0191; 0.1643] | 87.2% |
|  | Brazil | 6 (0, 912) | 0.0000 | [0.0000; 0.0000] | 0.0% |
|  | Yemen | 1 (0, 23) | 0.0000 | [0.0000; 0.0735] | -- |
|  | Egypt | 8 (8, 391) | 0.0047 | [0.0000; 0.0308] | 66.8% |
|  | Palestine | 1 (22, 24) | 0.9167 | [0.7650; 0.9984] | -- |
|  | Tanzania | 2 (0, 97) | 0.0000 | [0.0000; 0.0177] | 0.0% |
|  | Lebanon | 1 (0, 31) | 0.0000 | [0.0000; 0.0547] | -- |
|  | Vietnam | 1 (29, 33) | 0.8788 | [0.7416; 0.9721] | -- |
|  | India | 1 (0, 40) | 0.0000 | [0.0000; 0.0425] | -- |
|  | Saudi Arabia | 3 (0, 288) | 0.0000 | [0.0000; 0.0055] | 0.0% |
|  | Malaysia | 1 (0, 60) | 0.0000 | [0.0000; 0.0285] | -- |
|  | Italy | 2 (13, 3556) | 0.0366 | [0.0000; 0.2548] | 96.4% |
|  | Thailand | 1 (0, 65) | 0.0000 | [0.0000; 0.0263] | -- |
|  | Trinidad | 2 (47, 138) | 0.2260 | [0.0000; 0.9624] | 99.1% |
|  | Mozambique | 1 (0, 68) | 0.0000 | [0.0000; 0.0251] | -- |
|  | South Korea | 6 (0, 4286) | 0.0000 | [0.0000; 0.0000] | 0.0% |
|  | Japan | 3 (0, 692) | 0.0000 | [0.0000; 0.0016] | 0.0% |
|  | Argentina | 1 (0, 84) | 0.0000 | [0.0000; 0.0204] | -- |
|  | Denmark | 1 (0, 96) | 0.0000 | [0.0000; 0.0178] | -- |
|  | Ghana | 1 (0, 97) | 0.0000 | [0.0000; 0.0176] | -- |
|  | Taiwan | 1 (0, 100) | 0.0000 | [0.0000; 0.0171] | -- |
|  | USA | 7 (0, 1446) | 0.0000 | [0.0000; 0.0009] | 0.0% |
|  | Mexico | 1 (0, 101) | 0.0000 | [0.0000; 0.0170] | -- |
|  | Canada | 3 (0, 438) | 0.0000 | [0.0000; 0.0042] | 0.0% |
|  | South Africa | 2 (0, 282) | 0.0000 | [0.0000; 0.0068] | 0.0% |
|  | Emirates | 1 (7, 158) | 0.0443 | [0.0168; 0.0829] | -- |
|  | Ireland | 1 (0, 171) | 0.0000 | [0.0000; 0.0100] | -- |
|  | Czech Republic | 1 (0, 172) | 0.0000 | [0.0000; 0.0100] | -- |
|  | Emirates | 1 (7, 158) | 0.0443 | [0.0168; 0.0829] | -- |
| Time | 2011-2015 | 26 (1917, 6717) | 0.2511 | [0.1641; 0.3487] | 96.2% |
|  | 2016-2020 | 36 (1934, 6776) | 0.2683 | [0.1951; 0.3481] | 94.9% |
|  | 2006-2010 | 15 (402, 1916) | 0.1356 | [0.0718; 0.2143] | 95.9% |
|  | 2002-2005 | 9 (115, 1243) | 0.1122 | [0.0542; 0.1861] | 91.6% |
|  | 2021-2024 | 7 (164, 534) | 0.3207 | [0.1870; 0.4708] | 89.4% |
|  | Before 2001 | 10 (144, 2684) | 0.0721 | [0.0420; 0.1090] | 90.2% |
| Continent | Asia | 54 (2209, 8191) | 0.2796 | [0.2165; 0.3470] | 97.7% |
|  | Europe | 25 (2933, 11200) | 0.1666 | [0.1234; 0.2144] | 97.1% |
|  | South America | 13 (82, 1743) | 0.0745 | [0.0302; 0.1335] | 86.7% |
|  | Africa | 27 (297, 1540) | 0.1929 | [0.1159; 0.2830] | 93.5% |
|  | North America | 16 (693, 3751) | 0.1534 | [0.1020; 0.2128] | 96.0% |
|  | Oceania | 3 (84, 998) | 0.1601 | [0.0419; 0.3308] | 92.3% |
| Ampicillin |  |  |  |  |  |
| Overall | ND | 100(281, 19689) | 0.0184 | [0.0043; 0.0389] | 92.9% |
| Country | Iran | 7 (73, 256) | 0.1954 | [0.0186; 0.4624] | 96.3% |
|  | Nigeria | 3 (20, 57) | 0.4711 | [0.0000; 1.0000] | 96.7% |
|  | Bosnia and Herzegovina | 1 (0, 7) | 0.0000 | [0.0000; 0.2319] | -- |
|  | Turkey | 2 (0, 42) | 0.0000 | [0.0000; 0.0357] | 0.0% |
|  | France | 1 (0, 10) | 0.0000 | [0.0000; 0.1652] | -- |
|  | Cameroon | 1 (0, 14) | 0.0000 | [0.0000; 0.1193] | -- |
|  | China | 6 (3, 1538) | 0.0000 | [0.0000; 0.0006] | 0.0% |
|  | Ohangwena | 1 (0, 18) | 0.0000 | [0.0000; 0.0934] | -- |
|  | Sri Lanka | 2 (0, 63) | 0.0000 | [0.0000; 0.0271] | 0.0% |
|  | Ethiopia | 11 (59, 440) | 0.0780 | [0.0191; 0.1643] | 87.2% |
|  | Brazil | 6 (0, 912) | 0.0000 | [0.0000; 0.0000] | 0.0% |
|  | Yemen | 1 (0, 23) | 0.0000 | [0.0000; 0.0735] | -- |
|  | Egypt | 7 (8, 391) | 0.0047 | [0.0000; 0.0308] | 66.8% |
|  | Palestine | 1 (22, 24) | 0.9167 | [0.7650; 0.9984] | -- |
|  | Tanzania | 2 (0, 97) | 0.0000 | [0.0000; 0.0177] | 0.0% |
|  | Lebanon | 1 (0, 31) | 0.0000 | [0.0000; 0.0547] | -- |
|  | Vietnam | 1 (29, 33) | 0.8788 | [0.7416; 0.9721] | -- |
|  | India | 1 (0, 40) | 0.0000 | [0.0000; 0.0425] | -- |
|  | Saudi Arabia | 3 (0, 288) | 0.0000 | [0.0000; 0.0055] | -- |
|  | Malaysia | 1 (0, 60) | 0.0000 | [0.0000; 0.0285] | 0.0% |
|  | Italy | 2 (13, 3556) | 0.0366 | [0.0000; 0.2548] | -- |
|  | Thailand | 1 (0, 65) | 0.0000 | [0.0000; 0.0263] | 96.4% |
|  | Trinidad | 2 (47, 138) | 0.2260 | [0.0000; 0.9624] | -- |
|  | Mozambique | 1 (0, 68) | 0.0000 | [0.0000; 0.0251] | 99.1% |
|  | South Korea | 6 (0, 4286) | 0.0000 | [0.0000; 0.0000] | -- |
|  | Japan | 3 (0, 692) | 0.0000 | [0.0000; 0.0016] | 0.0% |
|  | Argentina | 1 (0, 84) | 0.0000 | [0.0000; 0.0204] | 0.0% |
|  | Denmark | 1 (0, 96) | 0.0000 | [0.0000; 0.0178] | -- |
|  | Ghana | 1 (0, 97) | 0.0000 | [0.0000; 0.0176] | -- |
|  | Taiwan | 1 (0, 100) | 0.0000 | [0.0000; 0.0171] | -- |
|  | USA | 7 (0, 1446) | 0.0000 | [0.0000; 0.0009] | -- |
|  | Mexico | 1 (0, 101) | 0.0000 | [0.0000; 0.0170] | 0.0% |
|  | Canada | 3 (0, 438) | 0.0000 | [0.0000; 0.0042] | -- |
|  | South Africa | 2 (0, 282) | 0.0000 | [0.0000; 0.0068] | 0.0% |
|  | Emirates | 1 (7, 158) | 0.0443 | [0.0168; 0.0829] | 0.0% |
|  | Ireland | 1 (0, 171) | 0.0000 | [0.0000; 0.0100] | -- |
|  | Czech Republic | 1 (0, 172) | 0.0000 | [0.0000; 0.0100] | -- |
|  | Poland | 1 (0, 953) | 0.0000 | [0.0000; 0.0018] | -- |
|  | Kuwait | 1 (0, 1166) | 0.0000 | [0.0000; 0.0015] | -- |
|  | Spain | 1 (0, 1276) | 0.0000 | [0.0000; 0.0013] | -- |
| Continent | Asia | 44 (142, 9964) | 0.0233 | [0.0009; 0.0637] | 94.2% |
|  | Africa | 25 (79, 1318) | 0.0402 | [0.0040; 0.0999] | 90.6% |
|  | Europe | 8 (13, 5288) | 0.0000 | [0.0000; 0.0176] | 78.6% |
|  | South America | 9 (47, 1134) | 0.0183 | [0.0000; 0.1155] | 96.4% |
|  | North America | 11 (0, 1985) | 0.0000 | [0.0000; 0.0007] | 0.0% |
| Time | 2011-2015 | 15 (16, 978) | 0.0023 | [0.0000; 0.0214] | 71.7% |
|  | 2016-2020 | 31 (179, 9612) | 0.0632 | [0.0085; 0.1512] | 96.5% |
|  | 2006-2010 | 11 (12, 1089) | 0.0051 | [0.0000; 0.0333] | 77.5% |
|  | 2021-2024 | 5 (67, 233) | 0.2171 | [0.0215; 0.5218] | 96.4% |
|  | Before 2001 | 10 (0, 2732) | 0.0000 | [0.0000; 0.0000] | 0.0% |
|  | 2002-2005 | 4 (0, 1559) | 0.0000 | [0.0000; 0.0000] | 0.0% |
|  | 2011-2015 | 15 (16, 978) | 0.0023 | [0.0000; 0.0214] | 71.7% |
| Penicillin |  |  |  |  |  |
| Overall | ND | 146 (237, 30272) | 0.0072 | [0.0016; 0.0152] | 86.0% |
| Country | Brazil | 10 (1, 507) | 0.0000 | [0.0000;0.0067] | 0.0% |
|  | China | 12 (1, 2470) | 0.0000 | [0.0000;0.0188] | 0.0% |
|  | Ethiopia | 13 (1, 523) | 0.1443 | [0.0281;0.3182] | 93.4% |
|  | Africa | 2 (1, 57) | 0.0351 | [0.0006;0.1029] | 0.0% |
|  | Algeria | 1 (15,15) | 0.3333 | [0.1130;0.5949] | – |
|  | Argentina | 1 (84,84) | 0.0000 | [0.0000;0.0204] | – |
|  | Australia | 3 (1196,1196) | 0.0000 | [0.0000;0.0005] | 0.0% |
|  | Belgium | 1 (262,262) | 0.0000 | [0.0000;0.0066] | – |
|  | Bosnia & Herzegovina | 1 (7,7) | 0.0000 | [0.0000;0.2319] | – |
|  | Cameroon | 1 (14,14) | 0.0000 | [0.0000;0.1193] | – |
|  | Canada | 6 (1695,1695) | 0.0000 | [0.0000;0.0000] | 0.0% |
|  | Czech Rep. | 1 (172,172) | 0.0000 | [0.0000;0.0100] | – |
|  | Egypt | 8 (420,420) | 0.0100 | [0.0000;0.0811] | 83.6% |
|  | Emirates | 1 (158,158) | 0.0949 | [0.0536;0.1461] | – |
|  | France | 1 (10,10) | 0.0000 | [0.0000;0.1652] | – |
|  | Gabon | 1 (109,109) | 0.0000 | [0.0000;0.0157] | – |
|  | Germany | 1 (140,140) | 0.0000 | [0.0000;0.0122] | – |
|  | Ghana | 1 (97,97) | 0.0000 | [0.0000;0.0176] | – |
|  | Greece | 2 (238,238) | 0.0000 | [0.0000;0.0253] | 0.0% |
|  | Hungary | 1 (3554,3554) | 0.0000 | [0.0000;0.0005] | – |
|  | India | 1 (40,40) | 0.0000 | [0.0000;0.0425] | – |
|  | Iran | 8 (303,303) | 0.0246 | [0.0000;0.1069] | 78.5% |
|  | Ireland | 3 (449,449) | 0.0000 | [0.0000;0.0011] | 0.0% |
|  | Italy | 4 (3677,3677) | 0.0106 | [0.0000;0.1244] | 87.4% |
|  | Japan | 3 (692,692) | 0.0000 | [0.0000;0.0016] | 0.0% |
|  | Kuwait | 1 (1166,1166) | 0.0000 | [0.0000;0.0015] | – |
|  | Lebanon | 2 (107,107) | 0.0000 | [0.0000;0.0516] | 0.0% |
|  | Lisbon | 1 (54,54) | 0.0000 | [0.0000;0.0316] | – |
|  | Lithuania | 1 (148,148) | 0.0000 | [0.0000;0.0116] | – |
|  | Malaysia | 1 (60,60) | 0.0000 | [0.0000;0.0285] | – |
|  | Mexico | 1 (101,101) | 0.0000 | [0.0000;0.0170] | – |
|  | Mozambique | 1 (68,68) | 0.0000 | [0.0000;0.0251] | – |
|  | Nepal | 1 (24,24) | 0.5000 | [0.2993;0.7007] | – |
|  | Netherland | 1 (365,365) | 0.0000 | [0.0000;0.0047] | – |
|  | New Zealand | 1 (52,52) | 0.0000 | [0.0000;0.0328] | – |
|  | Nigeria | 2 (51,51) | 0.2839 | [0.0000;1.0000] | 97.8% |
|  | Ohangwena | 1 (18,18) | 0.0000 | [0.0000;0.0934] | – |
|  | Poland | 2 (1035,1035) | 0.0000 | [0.0000;0.0201] | 0.0% |
|  | Romania | 1 (152,152) | 0.0000 | [0.0000;0.0113] | – |
|  | Saudi Arabia | 2 (238,238) | 0.0000 | [0.0000;0.0315] | 0.0% |
|  | Senegal | 1 (43,43) | 0.2791 | [0.1537;0.4238] | – |
|  | South Africa | 2 (282,282) | 0.0000 | [0.0000;0.0016] | 0.0% |
|  | South Korea | 8 (4549,4549) | 0.0000 | [0.0000;0.0000] | 0.0% |
|  | Spain | 1 (1276,1276) | 0.0000 | [0.0000;0.0013] | – |
|  | Sri Lanka | 2 (63,63) | 0.0000 | [0.0000;0.0882] | 0.0% |
|  | Switzerland | 2 (488,488) | 0.0000 | [0.0000;0.0153] | 0.0% |
|  | Taiwan | 2 (156,156) | 0.1210 | [0.0000;1.0000] | 98.3% |
|  | Tanzania | 2 (97,97) | 0.0342 | [0.0000;1.0000] | 73.5% |
|  | Thailand | 2 (84,84) | 0.0000 | [0.0000;0.1026] | 0.0% |
|  | Turkey | 4 (86,86) | 0.0000 | [0.0000;0.0023] | 0.0% |
|  | Uganda | 1 (14,14) | 0.3571 | [0.1229;0.6292] | – |
|  | UK | 1 (535,535) | 0.0000 | [0.0000;0.0032] | – |
|  | Ukraine | 1 (149,149) | 0.0000 | [0.0000;0.0115] | – |
|  | USA | 7 (1331,1331) | 0.0013 | [0.0000;0.0260] | 74.0% |
|  | Yemen | 1 (23,23) | 0.0000 | [0.0000;0.0735] | – |
| Continent | Asia | 57 (80, 11638) | 0.0032 | [0.0000; 0.0130] | 83.6% |
|  | Europe | 25 (13, 11758) | 0.0000 | [0.0000; 0.0000] | 40.1% |
|  | South America | 11 (0, 846) | 0.0000 | [0.0000; 0.0002] | 0.0% |
|  | Africa | 31 (138, 1655) | 0.0707 | [0.0229; 0.1371] | 92.5% |
|  | North America | 14 (6, 3127) | 0.0000 | [0.0000; 0.0004] | 41.9% |
|  | Oceania | 4 (0, 1248) | 0.0000 | [0.0000; 0.0000] | 0.0% |
| Time | 2011-2015 | 27 (24, 6498) | 0.0008 | [0.0000; 0.0102] | 75.4% |
|  | 2016-2020 | 35 (131, 9720) | 0.0369 | [0.0050; 0.0880] | 94.0% |
|  | 2006-2010 | 21 (14, 2619) | 0.0004 | [0.0000; 0.0057] | 62.3% |
|  | 2002-2005 | 9 (22, 2318) | 0.0078 | [0.0000; 0.0570] | 91.8% |
|  | 2021-2024 | 7 (26, 534) | 0.0490 | [0.0028; 0.1330] | 90.3% |
|  | Before 2001 | 12 (0, 3035) | 0.0000 | [0.0000; 0.0000] | 0.0% |
| Erythromycin |  |  |  |  |  |
| Overall | ND | 159 (7549, 30841) | 0.2151 | [0.1819; 0.2502] | 98.0% |
| Country | Iran | 7 (62, 296) | 0.2526 | [0.0310; 0.5688] | 95.9% |
|  | Nigeria | 4 (34, 95) | 0.3431 | [0.2258; 0.4696] | 17.4% |
|  | Bosnia and Herzegovina | 1 (1, 7) | 0.1429 | [0.0000; 0.5169] | -- |
|  | Turkey | 3 (14, 66) | 0.2079 | [0.1123; 0.3203] | 0.0% |
|  | France | 3 (60, 1320) | 0.0753 | [0.0000; 0.2858] | 98.5% |
|  | Brazil | 11 (158, 1382) | 0.0798 | [0.0375; 0.1339] | 78.7% |
|  | Uganda | 1 (3, 14) | 0.2143 | [0.0325; 0.4734] | -- |
|  | Cameroon | 1 (1, 14) | 0.0714 | [0.0000; 0.2816] | -- |
|  | Algeria | 1 (11, 15) | 0.7333 | [0.4767; 0.9319] | -- |
|  | China | 14 (1534, 3044) | 0.6153 | [0.5169; 0.7094] | 98.4% |
|  | Ohangwena | 1 (0, 18) | 0.0000 | [0.0000; 0.0934] | -- |
|  | Sri Lanka | 2 (12, 63) | 0.1528 | [0.0177; 0.3654] | 67.7% |
|  | Thailand | 2 (6, 84) | 0.0980 | [0.0000; 0.4459] | 89.7% |
|  | Ethiopia | 13 (104, 545) | 0.1657 | [0.0979; 0.2456] | 78.2% |
|  | Egypt | 8 (117, 350) | 0.3128 | [0.1936; 0.4453] | 84.8% |
|  | Nepal | 1 (8, 24) | 0.3333 | [0.1563; 0.5364] | -- |
|  | Palestine | 1 (7, 24) | 0.2917 | [0.1242; 0.4917] | -- |
|  | South Korea | 6 (195, 800) | 0.2393 | [0.2045; 0.2759] | 21.9% |
|  | Tanzania | 2 (17, 97) | 0.1907 | [0.0620; 0.3633] | 67.4% |
|  | Lebanon | 2 (20, 107) | 0.1900 | [0.1031; 0.2943] | 30.2% |
|  | Vietnam | 1 (8, 33) | 0.2424 | [0.1091; 0.4051] | -- |
|  | USA | 9 (588, 2164) | 0.2065 | [0.1128; 0.3192] | 97.7% |
|  | Senegal | 1 (23, 43) | 0.5349 | [0.3839; 0.6828] | -- |
|  | South Africa | 3 (92, 331) | 0.4831 | [0.0000; 0.9974] | 99.0% |
|  | Australia | 3 (78, 1196) | 0.1056 | [0.0035; 0.3069] | 93.6% |
|  | Saudi Arabia | 3 (43, 288) | 0.1474 | [0.1079; 0.1915] | 0.0% |
|  | Italy | 5 (1490, 3998) | 0.2137 | [0.0979; 0.3583] | 97.2% |
|  | New Zealand | 1 (4, 52) | 0.0769 | [0.0171; 0.1680] | -- |
|  | Lisbon | 1 (4, 54) | 0.0741 | [0.0165; 0.1620] | -- |
|  | Taiwan | 2 (62, 156) | 0.3483 | [0.0878; 0.6700] | 93.6% |
|  | South Africa | 1 (1, 57) | 0.0175 | [0.0000; 0.0738] | -- |
|  | Malaysia | 1 (14, 60) | 0.2333 | [0.1339; 0.3496] | -- |
|  | Poland | 3 (322, 1415) | 0.1913 | [0.1212; 0.2727] | 79.7% |
|  | Trinidad | 2 (27, 138) | 0.1768 | [0.0153; 0.4462] | 91.9% |
|  | Greece | 2 (38, 238) | 0.1166 | [0.0089; 0.3102] | 91.3% |
|  | Mozambique | 1 (34, 68) | 0.5000 | [0.3810; 0.6190] | -- |
|  | Israel | 1 (4, 72) | 0.0556 | [0.0122; 0.1227] | -- |
|  | Japan | 3 (195, 692) | 0.2513 | [0.1020; 0.4387] | 94.5% |
|  | Ireland | 3 (88, 449) | 0.1955 | [0.1597; 0.2338] | 0.0% |
|  | Argentina | 2 (19, 377) | 0.0493 | [0.0277; 0.0761] | 7.2% |
|  | Denmark | 1 (20, 96) | 0.2083 | [0.1324; 0.2959] | -- |
|  | Ghana | 1 (1, 97) | 0.0103 | [0.0000; 0.0437] | -- |
|  | Mexico | 1 (6, 101) | 0.0594 | [0.0202; 0.1153] | -- |
|  | Canada | 7 (336, 1587) | 0.2128 | [0.1371; 0.2997] | 92.5% |
|  | Gabon | 1 (0, 109) | 0.0000 | [0.0000; 0.0157] | -- |
|  | Switzerland | 2 (91, 488) | 0.2177 | [0.0827; 0.3935] | 92.9% |
|  | Germany | 1 (30, 140) | 0.2143 | [0.1499; 0.2864] | -- |
|  | Lithuania | 1 (6, 148) | 0.0405 | [0.0137; 0.0794] | -- |
|  | Ukraine | 1 (45, 149) | 0.3020 | [0.2307; 0.3784] | -- |
|  | Romania | 1 (38, 152) | 0.2500 | [0.1841; 0.3222] | -- |
|  | Emirates | 1 (90, 158) | 0.5696 | [0.4915; 0.6460] | -- |
|  | Czech Republic | 1 (7, 172) | 0.0407 | [0.0154; 0.0762] | -- |
|  | Malasiya | 1 (8, 200) | 0.0400 | [0.0166; 0.0723] | -- |
|  | Belgium | 1 (44, 262) | 0.1679 | [0.1250; 0.2158] | -- |
|  | Netherland | 1 (7, 365) | 0.0192 | [0.0072; 0.0362] | -- |
|  | Spain | 2 (75, 1651) | 0.0552 | [0.0000; 0.3956] | 99.6% |
|  | Kuwait | 1 (8, 1166) | 0.0069 | [0.0028; 0.0125] | -- |
|  | Hungary | 1 (1239, 3554) | 0.3486 | [0.3330; 0.3644] | -- |
| Continent | Asia | 57 (2596, 8461) | 0.3151 | [0.2481; 0.3860] | 98.1% |
|  | Africa | 34 (366, 1678) | 0.2088 | [0.1311; 0.2977] | 93.9% |
|  | Europe | 29 (3371, 13705) | 0.1488 | [0.1059; 0.1973] | 99.1% |
|  | South America | 15 (204, 1897) | 0.0872 | [0.0483; 0.1349] | 83.1% |
|  | North America | 17 (930, 3852) | 0.1988 | [0.1382; 0.2671] | 96.6% |
|  | Oceania | 4 (82, 1248) | 0.0970 | [0.0157; 0.2300] | 90.5% |
| Time | 2011–2015 | 28 (2008, 6350) | 0.2746 | [0.1820; 0.3774] | 95.5% |
|  | 2016–2020 | 38 (2367, 7796) | 0.3024 | [0.2256; 0.3847] | 98.0% |
|  | 2006–2010 | 20 (373, 2382) | 0.1077 | [0.0700; 0.1517] | 89.1% |
|  | 2002–2005 | 11 (175, 2859) | 0.0785 | [0.0291; 0.1470] | 97.4% |
|  | 2021–2024 | 8 (174, 572) | 0.3115 | [0.2165; 0.4149] | 80.3% |
|  | Before 2001 | 14 (257, 3173) | 0.0937 | [0.0555; 0.1400] | 95.8% |
| SXT |  |  |  |  |  |
| Overall | ND | 19 (665, 6221) | 0.3931 | [0.2142; 0.5874] | 99.3% |
| Country | Iran | 2 (54, 63) | 0.9029 | [0.7308; 0.9985] | 34.3% |
|  | Uganda | 1 (12, 14) | 0.8571 | [0.6159; 0.9967] | -- |
|  | Ethiopia | 1 (9, 31) | 0.2903 | [0.1419; 0.4642] | -- |
|  | Jordan | 1 (9, 38) | 0.2368 | [0.1132; 0.3866] | -- |
|  | Brazil | 1 (7, 39) | 0.1795 | [0.0724; 0.3176] | -- |
|  | Australia | 1 (0, 50) | 0.0000 | [0.0000; 0.0341] | -- |
|  | Taiwan | 1 (31, 56) | 0.5536 | [0.4213; 0.6822] | -- |
|  | Africa | 1 (54, 57) | 0.9474 | [0.8709; 0.9932] | -- |
|  | Italy | 1 (3, 65) | 0.0462 | [0.0059; 0.1137] | -- |
|  | Trinidad | 2 (100, 138) | 0.7445 | [0.4187; 0.9662] | 93.7% |
|  | Tanzania | 1 (11, 69) | 0.1594 | [0.0813; 0.2563] | -- |
|  | Argentina | 1 (33, 84) | 0.3929 | [0.2907; 0.4999] | -- |
|  | South Africa | 1 (17, 154) | 0.1104 | [0.0652; 0.1652] | -- |
|  | Emirates | 1 (122, 158) | 0.7722 | [0.7032; 0.8345] | -- |
|  | Spain | 2 (203, 1651) | 0.1446 | [0.0636; 0.2512] | 95.4% |
|  | Hungary | 1 (0, 3554) | 0.0000 | [0.0000; 0.0005] | -- |
| Continent | Asia | 5 (216, 315) | 0.6941 | [0.4113; 0.9184] | 92.7% |
|  | Africa | 5 (103, 325) | 0.4705 | [0.1165; 0.8418] | 97.9% |
|  | South America | 4 (140, 261) | 0.5171 | [0.2166; 0.8111] | 95.7% |
|  | Oceania | 1 (0, 50) | 0.0000 | [0.0000; 0.0341] | -- |
|  | Europe | 4 (206, 5270) | 0.0621 | [0.0022; 0.1851] | 99.5% |
| Time | 2011–2015 | 5 (98, 4039) | 0.2011 | [0.0024; 0.5581] | 99.0% |
|  | 2016–2020 | 5 (190, 472) | 0.2932 | [0.0424; 0.6428] | 98.4% |
|  | 2002–2005 | 2 (160, 1332) | 0.2957 | [0.0018; 0.7810] | 98.3% |
|  | 2006–2010 | 2 (58, 125) | 0.4978 | [0.0000; 0.9999] | 98.5% |
|  | Before 2001 | 1 (58, 66) | 0.8788 | [0.7875; 0.9482] | -- |
| Linezolid |  |  |  |  |  |
| Overall | ND | 7 (29, 3747) | 0 | 0 | 0 |
| Country | Bosnia and Herzegovina | 1 (0, 7) | 0.0000 | [0.0000; 0.2319] | -- |
|  | China | 10 (0, 2366) | 0.0000 | [0.0000; 0.0000] | 0.0% |
|  | Ohangwena | 1 (0, 18) | 0.0000 | [0.0000; 0.0934] | -- |
|  | Egypt | 3 (0, 112) | 0.0000 | [0.0000; 0.0170] | 0.0% |
|  | Nepal | 1 (0, 24) | 0.0000 | [0.0000; 0.0704] | -- |
|  | Nigeria | 1 (0, 33) | 0.0000 | [0.0000; 0.0515] | -- |
|  | Iran | 1 (0, 41) | 0.0000 | [0.0000; 0.0415] | -- |
|  | Senegal | 1 (0, 43) | 0.0000 | [0.0000; 0.0396] | -- |
|  | Saudi Arabia | 1 (0, 60) | 0.0000 | [0.0000; 0.0285] | -- |
|  | Italy | 1 (0, 62) | 0.0000 | [0.0000; 0.0275] | -- |
|  | Ethiopia | 1 (0, 83) | 0.0000 | [0.0000; 0.0206] | -- |
|  | Argentina | 1 (0, 84) | 0.0000 | [0.0000; 0.0204] | -- |
|  | Gabon | 1 (0, 109) | 0.0000 | [0.0000; 0.0157] | -- |
|  | USA | 1 (0, 117) | 0.0000 | [0.0000; 0.0146] | -- |
|  | Switzerland | 1 (0, 124) | 0.0000 | [0.0000; 0.0138] | -- |
|  | Romania | 1 (3, 152) | 0.0197 | [0.0024; 0.0494] | -- |
|  | South Africa | 1 (0, 154) | 0.0000 | [0.0000; 0.0111] | -- |
|  | Emirates | 1 (4, 158) | 0.0253 | [0.0054; 0.0568] | -- |
| Continent | Europe | 4 (3, 345) | 0.0000 | [0.0000; 0.0105] | 18.1% |
|  | Asia | 17 (4, 2741) | 0.0000 | [0.0000; 0.0003] | 5.7% |
|  | Africa | 6 (0, 460) | 0.0000 | [0.0000; 0.0023] | 0.0% |
|  | South America | 1 (0, 84) | 0.0000 | [0.0000; 0.0204] | -- |
|  | North America | 1 (0, 117) | 0.0000 | [0.0000; 0.0146] | -- |
| Time | 2011–2015 | 5 (0, 367) | 0.0000 | [0.0000; 0.0003] | 0.0% |
|  | 2016–2020 | 11 (4, 1785) | 0.0000 | [0.0000; 0.0028] | 33.0% |
|  | Before 2001 | 1 (0, 117) | 0.0000 | [0.0000; 0.0146] | -- |
|  | 2021–2024 | 1 (3, 152) | 0.0197 | [0.0024; 0.0494] | -- |
| Azithromycin |  |  |  |  |  |
| Overall | ND | 13 (346, 1159) | 0.3127 | [0.1898; 0.4501] | 94.6% |
| Country | Ethiopia | 2 (22, 127) | 0.1669 | [0.0987; 0.2475] | 12.3% |
|  | Egypt | 2 (22, 89) | 0.2458 | [0.1602; 0.3423] | 0.0% |
|  | Nigeria | 1 (13, 38) | 0.3421 | [0.1983; 0.5015] | -- |
|  | Taiwan | 1 (20, 56) | 0.3571 | [0.2361; 0.4879] | -- |
|  | China | 2 (107, 147) | 0.7633 | [0.5018; 0.9491] | 90.7% |
|  | Trinidad | 1 (3, 72) | 0.0417 | [0.0053; 0.1029] | -- |
|  | Italy | 1 (13, 73) | 0.1781 | [0.0978; 0.2751] | -- |
|  | Japan | 1 (38, 76) | 0.5000 | [0.3874; 0.6126] | -- |
|  | USA | 1 (34, 106) | 0.3208 | [0.2349; 0.4130] | -- |
|  | Spain | 1 (74, 375) | 0.1973 | [0.1585; 0.2392] | -- |
| Continent | Africa | 4 (50, 218) | 0.2268 | [0.1422; 0.3236] | 56.2% |
|  | Asia | 5 (172, 315) | 0.5211 | [0.2827; 0.7547] | 93.5% |
|  | South America | 1 (3, 72) | 0.0417 | [0.0053; 0.1029] | -- |
|  | Europe | 2 (87, 448) | 0.1930 | [0.1574; 0.2313] | 0.0% |
|  | North America | 1 (34, 106) | 0.3208 | [0.2349; 0.4130] | -- |
| Time | 2021–2024 | 2 (16, 67) | 0.2148 | [0.0335; 0.4814] | 81.1% |
|  | 2016–2020 | 3 (29, 206) | 0.1309 | [0.0392; 0.2611] | 81.8% |
|  | 2002–2005 | 1 (20, 56) | 0.3571 | [0.2361; 0.4879] | -- |
|  | 2011–2015 | 3 (181, 522) | 0.5731 | [0.1740; 0.9228] | 98.6% |
|  | 2006–2010 | 1 (34, 106) | 0.3208 | [0.2349; 0.4130] | -- |
| Gentamycin |  |  |  |  |  |
| Overall | ND | 14 (304, 891) | 0.3336 | [0.0973; 0.6219] | 98.8% |
| Country | Nigeria | 1 (4, 6) | 0.6667 | [0.2364; 0.9870] | -- |
|  | Cameroon | 1 (5, 14) | 0.3571 | [0.1229; 0.6292] | -- |
|  | Ethiopia | 3 (22, 70) | 0.2695 | [0.0000; 0.9986] | 98.3% |
|  | Jordan | 1 (17, 30) | 0.5667 | [0.3846; 0.7404] | -- |
|  | Brazil | 1 (35, 46) | 0.7609 | [0.6257; 0.8746] | -- |
|  | South Africa | 2 (25, 177) | 0.1599 | [0.0000; 0.8408] | 98.8% |
|  | Africa | 1 (53, 57) | 0.9298 | [0.8462; 0.9845] | -- |
|  | Canada | 1 (0, 102) | 0.0000 | [0.0000; 0.0168] | -- |
|  | Gabon | 1 (0, 109) | 0.0000 | [0.0000; 0.0157] | -- |
|  | Switzerland | 1 (1, 124) | 0.0081 | [0.0000; 0.0343] | -- |
|  | USA | 1 (142,156) | 0.9103 | [0.8598; 0.9507] | -- |
| Continent | Africa | 4 (50, 218) | 0.2268 | [0.1422; 0.3236] | 56.2% |
|  | Asia | 5 (172, 315) | 0.5211 | [0.2827; 0.7547] | 93.5% |
|  | South America | 1 (3,72) | 0.0417 | [0.0053; 0.1029] | -- |
|  | Europe | 2 (87, 448) | 0.1930 | [0.1574; 0.2313] | 0.0% |
|  | North America | 1 (34, 106) | 0.3208 | [0.2349; 0.4130] | -- |
| Time | 2016–2020 | 4 (51, 99) | 0.5155 | [0.4115; 0.6188] | 0.0% |
|  | 2011–2015 | 5 (23, 395) | 0.1098 | [0.0000; 0.6078] | 97.6% |
|  | 2006–2010 | 1 (0, 29 0) | 0.0000 | [0.0000; 0.0585] | -- |
|  | 2002–2005 | 1 (35, 46) | 0.7609 | [0.6257; 0.8746] | -- |
| Cefazolin |  |  |  |  |  |
| Overall | ND | 15 (40, 1686) | 0.0134 | [0.0000; 0.0517] | 98.04% |
| Country | Iran | 3 (11, 90) | 0.0664 | [0.0000; 0.4133] | 92.7% |
|  | Brazil | 3 (0, 145) | 0.0000 | [0.0000; 0.0108] | 0.0% |
|  | Turkey | 1 (1, 33) | 0.0303 | [0.0000; 0.1255] | -- |
|  | Vietnam | 1 (10, 33) | 0.3030 | [0.1563; 0.4722] | -- |
|  | Thailand | 1 (0, 65) | 0.0000 | [0.0000; 0.0263] | -- |
|  | USA | 3 (4, 510) | 0.0040 | [0.0000; 0.0255] | 74.4% |
|  | South Korea | 2 (14, 548) | 0.0187 | [0.0001; 0.0606] | 83.8% |
|  | Belgium | 1 (0, 262) | 0.0000 | [0.0000; 0.0066] | -- |
| Continent | Asia | 8 (36, 769) | 0.0452 | [0.0000; 0.1527] | 89.1% |
|  | South America | 3 (0, 145) | 0.0000 | [0.0000; 0.0108] | 0.0% |
|  | North America | 3 (4, 510) | 0.0040 | [0.0000; 0.0255] | 74.4% |
|  | Europe | 1 (0, 262) | 0.0000 | [0.0000; 0.0066] | -- |
| Time | 2011–2015 | 1 (0, 4) | 0.0000 | [0.0000; 0.3885] | -- |
|  | 2006–2010 | 4 (24, 459) | 0.0525 | [0.0000; 0.2347] | 90.0% |
|  | Before 2001 | 3 (1, 389) | 0.0000 | [0.0000; 0.0044] | 45.6% |
|  | 2016–2020 | 2 (10, 118) | 0.0891 | [0.0000; 0.5573] | 96.4% |
| Ceftriaxone |  |  |  |  |  |
| Overall | ND | 40 (106, 6439) | 0.0230 | [0.0055; 0.0485] | 90.6% |
| Country | Nigeria | 4 (3, 95) | 0.0174 | [0.0000; 0.1179] | 60.2% |
|  | Iran | 3 (21, 99) | 0.2588 | [0.0552; 0.5340] | 82.6% |
|  | Ethiopia | 7 (61, 282) | 0.1652 | [0.0814; 0.2686] | 73.3% |
|  | Egypt | 4 (0, 159) | 0.0000 | [0.0000; 0.0125] | 0.0% |
|  | Nepal | 1 (0, 24) | 0.0000 | [0.0000; 0.0704] | -- |
|  | South Korea | 4 (0, 3688) | 0.0000 | [0.0000; 0.0000] | 0.0% |
|  | Lebanon | 2 (0, 107) | 0.0000 | [0.0000; 0.0160] | 0.0% |
|  | Australia | 1 (0, 50) | 0.0000 | [0.0000; 0.0341] | -- |
|  | Saudi Arabia | 1 (0, 50) | 0.0000 | [0.0000; 0.0341] | -- |
|  | China | 4 (0, 1025, 0) | 0.0000 | [0.0000; 0.0006] | 0.0% |
|  | Malaysia | 1 (0, 60) | 0.0000 | [0.0000; 0.0285] | -- |
|  | Mozambique | 1 (0, 68) | 0.0000 | [0.0000; 0.0251] | -- |
|  | Tanzania | 1 (11, 69) | 0.1594 | [0.0813; 0.2563] | -- |
|  | Trinidad | 1 (4, 72) | 0.0556 | [0.0122; 0.1227] | -- |
|  | Brazil | 2 (0, 173) | 0.0000 | [0.0000; 0.0112] | 0.0% |
|  | USA | 1 (0,106) | 0.0000 | [0.0000; 0.0162] | -- |
|  | South Africa | 1 ( 0, 154) | 0.0000 | [0.0000; 0.0111] | -- |
|  | Emirates | 1 (6, 158) | 0.0380 | [0.0128; 0.0744] | -- |
|  | Nigeria | 4 (3, 95) | 0.0174 | [0.0000; 0.1179] | 60.2% |
| Continent | Africa | 16 (75, 753) | 0.0634 | [0.0166; 0.1305] | 89.8% |
|  | Asia | 20 (27, 5303) | 0.0082 | [0.0000; 0.0315] | 84.2% |
|  | Oceania | 1 (0, 50) | 0.0000 | [0.0000; 0.0341] | -- |
|  | South America | 3 (4, 245) | 0.0084 | [0.0000; 0.0530] | 74.8% |
|  | North America | 1 (0, 106) | 0.0000 | [0.0000; 0.0162] | -- |
| Time | 2016–2020 | 18 (56, 4510) | 0.0234 | [0.0006; 0.0659] | 92.6% |
|  | 2011–2015 | 5 (3, 405) | 0.0023 | [0.0000; 0.0303] | 61.5% |
|  | 2006–2010 | 7 (21, 450) | 0.0292 | [0.0000; 0.0935] | 86.8% |
|  | 2021–2024 | 3 (17, 121) | 0.1016 | [0.0000; 0.3122] | 88.2% |
| Levofloxacin |  |  |  |  |  |
| Overall | ND | 15 (1181, 10907) | 0.1125 | [0.0696; 0.1632] | 97.4% |
| Country | Bosnia & Herzegovina | 1 (0, 7) | 0.0000 | [0.0000; 0.2319] | -- |
|  | Algeria | 1 (13, 15) | 0.8667 | [0.6388; 0.9970] | -- |
|  | China | 12 (797, 2964) | 0.3014 | [0.2052; 0.4069] | 97.0% |
|  | Yemen | 1 (0, 23) | 0.0000 | [0.0000; 0.0735] | -- |
|  | Egypt | 4 (46, 166) | 0.2122 | [0.0270; 0.4928] | 91.6% |
|  | Nepal | 1 (4, 24) | 0.1667 | [0.0396; 0.3464] | -- |
|  | Palestine | 1 (2, 24) | 0.0833 | [0.0016; 0.2350] | -- |
|  | South Korea | 3 (22, 338) | 0.0827 | [0.0051; 0.2230] | 91.5% |
| Continent | Europe | 7 (190, 4296) | 0.0256 | [0.0006; 0.0723] | 90.5% |
|  | Africa | 6 (52, 386) | 0.2092 | [0.0184; 0.5109] | 96.2% |
|  | Asia | 30 (895, 4989) | 0.1567 | [0.0971; 0.2266] | 97.7% |
|  | Oceania | 1 (2, 50) | 0.0400 | [0.0007; 0.1168] | -- |
|  | South America | 5 (42, 1069) | 0.0188 | [0.0002; 0.0568] | 85.4% |
|  | North America | 1 (0, 117) | 0.0000 | [0.0000; 0.0146] | -- |
| Time | 2011–2015 | 9 (138, 1168) | 0.0855 | [0.0131; 0.2012] | 97.2% |
|  | 2016–2020 | 19 (513, 5629) | 0.1606 | [0.0728; 0.2719] | 96.9% |
|  | 2006–2010 | 4 (7, 427) | 0.0082 | [0.0000; 0.0354] | 60.0% |
|  | 2021–2024 | 4 (36, 301) | 0.1176 | [0.0824; 0.1576] | 0.0% |
|  | Before 2001 | 1 (0, 117) | 0.0000 | [0.0000; 0.0146] | -- |
| Norfloxacin |  |  |  |  |  |
| Overall | ND | 11 (180, 740) | 0.1655 | [0.0495; 0.3252] | 94.1% |
| Country | Algeria | 1 (4, 15) | 0.2667 | [0.0681; 0.5233] | -- |
|  | Ethiopia | 3 (8, 79) | 0.0991 | [0.0380; 0.1798] | 0.0% |
|  | Jordan | 1 (26, 30) | 0.8667 | [0.7177; 0.9691] | -- |
|  | Brazil | 2 (0, 85) | 0.0000 | [0.0000; 0.0226] | 0.0% |
|  | Trinidad | 1 (7, 72) | 0.0972 | [0.0378; 0.1780] | -- |
|  | Italy | 1 (7, 73) | 0.0959 | [0.0373; 0.1757] | -- |
|  | Ukraine | 1 (38, 149) | 0.2550 | [0.1880; 0.3284] | -- |
|  | South Korea | 1 (90, 237) | 0.3797 | [0.3189; 0.4425] | -- |
| Continent | Africa | 4 (12, 94) | 0.1205 | [0.0574; 0.1992] | 0.0% |
|  | Asia | 2 (116, 267) | 0.6311 | [0.1527; 0.9857] | 96.5% |
|  | South America | 3 (7, 157) | 0.0185 | [0.0000; 0.1001] | 79.7% |
|  | Europe | 2 (45, 222) | 0.1718 | [0.0469; 0.3502] | 88.2% |
| Time | 2016–2020 | 3 (37, 117) | 0.3986 | [0.0174; 0.8770] | 96.8% |
|  | 2011–2015 | 2 (5, 50) | 0.0967 | [0.0243; 0.2005] | 0.0% |
|  | 2006–2010 | 2 (93, 266) | 0.2402 | [0.0344; 0.5427] | 90.5% |
|  | 2002–2005 | 1 (0, 46) | 0.0000 | [0.0000; 0.0370] | -- |
|  | 2021–2024 | 1 (38, 149) | 0.2550 | [0.1880; 0.3284] | -- |
| Moxifloxacin |  |  |  |  |  |
| Overall | ND | 6 (37, 605) | 0.0458 | [0.0007; 0.1372] | 91.7% |
| Country | Senegal | 1 (7, 43) | 0.1628 | [0.0652; 0.2901] | -- |
|  | Australia | 1 (0, 50) | 0.0000 | [0.0000; 0.0341] | -- |
|  | Italy | 1 (0, 60) | 0.0000 | [0.0000; 0.0285] | -- |
|  | China | 1 (23, 91) | 0.2527 | [0.1682; 0.3476] | -- |
|  | Switzerland | 1 (2, 124) | 0.0161 | [0.0003; 0.0480] | -- |
|  | South Korea | 1 (5, 237) | 0.0211 | [0.0059; 0.0441] | -- |
| Continent | Africa | 1 (7, 43) | 0.1628 | [0.0652; 0.2901] | -- |
|  | Oceania | 1 (0, 50) | 0.0000 | [0.0000; 0.0341] | -- |
|  | Europe | 2 (2, 184) | 0.0081 | [0.0000; 0.0295] | 0.0% |
|  | Asia | 2 (28, 328) | 0.1076 | [0.0000; 0.4230] | 97.3% |
| Time | 2016–2020 | 1 (0, 50) | 0.0000 | [0.0000; 0.0341] | -- |
|  | 2011–2015 | 3 (25, 275) | 0.0542 | [0.0000; 0.2510] | 95.3% |
|  | 2006–2010 | 1 (5, 237) | 0.0211 | [0.0059; 0.0441] | -- |
| Chloramphenicol |  |  |  |  |  |
| Overall | ND | 33 (264, 4932) | 0.0888 | [0.0385; 0.1544] | 94.7% |
| Country | Algeria | 1 (15, 15) | 1.0000 | [0.8884; 1.0000] | -- |
|  | Ohangwena | 1 (0, 18) | 0.0000 | [0.0000; 0.0934] | -- |
|  | Ethiopia | 9 (59, 387) | 0.1359 | [0.0387; 0.2727] | 87.1% |
|  | Nepal | 1 (8, 24) | 0.3333 | [0.1563; 0.5364] | -- |
|  | Turkey | 1 (0, 24) | 0.0000 | [0.0000; 0.0704] | -- |
|  | South Korea | 2 (4, 101) | 0.0370 | [0.0053; 0.0875] | 0.0% |
|  | Nigeria | 1 (8, 33) | 0.2424 | [0.1091; 0.4051] | -- |
|  | Senegal | 1 (1, 43) | 0.0233 | [0.0000; 0.0971] | -- |
|  | South Africa | 2 (41, 177) | 0.2719 | [0.0724; 0.5358] | 90.7% |
|  | Australia | 1 (0, 50) | 0.0000 | [0.0000; 0.0341] | -- |
|  | Italy | 1 (0, 52) | 0.0000 | [0.0000; 0.0328] | -- |
|  | Iran | 1 (1, 60) | 0.0167 | [0.0000; 0.0702] | -- |
|  | Malaysia | 1 (0, 60) | 0.0000 | [0.0000; 0.0285] | -- |
|  | Greece | 1 (0, 67) | 0.0000 | [0.0000; 0.0255] | -- |
|  | China | 3 (63, 1282) | 0.1254 | [0.0066; 0.3491] | 97.7% |
|  | Lebanon | 1 (3, 76) | 0.0395 | [0.0050; 0.0976] | -- |
|  | Ghana | 1 (12, 97) | 0.1237 | [0.0647; 0.1975] | -- |
|  | Brazil | 2 (35, 825) | 0.0173 | [0.0000; 0.0983] | 93.7% |
|  | Spain | 1 (14, 375) | 0.0373 | [0.0202; 0.0592] | -- |
|  | Kuwait | 1 (0, 1166) | 0.0000 | [0.0000; 0.0015] | -- |
| Continent | Africa | 15 (128, 737) | 0.1776 | [0.0606; 0.3336] | 91.4% |
|  | Asia | 12 (87, 2826) | 0.0609 | [0.0136; 0.1329] | 95.0% |
|  | Oceania | 1 (0, 50) | 0.0000 | [0.0000; 0.0341] | -- |
|  | Europe | 3 (14, 494) | 0.0100 | [0.0000; 0.0435] | 69.1% |
|  | South America | 2 (35, 825) | 0.0173 | [0.0000; 0.0983] | 93.7% |
| Time | 2016–2020 | 13 (148, 1725) | 0.2059 | [0.0699; 0.3851] | 96.0% |
|  | 2011–2015 | 6 (47, 796) | 0.0359 | [0.0007; 0.1042] | 90.2% |
|  | 2002–2005 | 2 (0, 76) | 0.0000 | [0.0000; 0.0233] | 0.0% |
|  | 2006–2010 | 3 (19, 131) | 0.1526 | [0.0000; 0.5045] | 93.6% |
|  | 2021–2024 | 4 (14, 179) | 0.0711 | [0.0139; 0.1591] | 68.8% |
|  | Before 2001 | 2 (0, 1233) | 0.0000 | [0.0000; 0.0000] | 0.0% |
| Cefotaxime |  |  |  |  |  |
| Overall | ND | 41 (177, 7465) | 0.0458 | [0.0075; 0.1065] | 95.6% |
| Country | Bosnia and Herzegovina | 1 (0, 7) | 0.0000 | [0.0000; 0.2319] | -- |
|  | Iran | 2 (30, 46) | 0.8042 | [0.1467; 1.0000] | 95.2% |
|  | Sri Lanka | 2 (0, 63) | 0.0000 | [0.0000; 0.0271] | 0.0% |
|  | Yemen | 1 (0, 23) | 0.0000 | [0.0000; 0.0735] | -- |
|  | Egypt | 7 (59, 303) | 0.1150 | [0.0000; 0.3805] | 96.7% |
|  | Palestine | 1 (11, 24) | 0.4583 | [0.2615; 0.6616] | -- |
|  | Lebanon | 1 (0, 31) | 0.0000 | [0.0000; 0.0547] | -- |
|  | Brazil | 2 (0, 168) | 0.0000 | [0.0000; 0.0080] | 0.0% |
|  | Nigeria | 2 (33, 71) | 0.3493 | [0.0000; 1.0000] | 98.8% |
|  | India | 1 (0, 40) | 0.0000 | [0.0000; 0.0425] | -- |
|  | Ethiopia | 2 (14, 124) | 0.1035 | [0.0000; 0.6162] | 97.3% |
|  | South Africa | 2 (22, 203) | 0.1495 | [0.0000; 0.7191] | 98.3% |
|  | New Zealand | 1 (0, 52) | 0.0000 | [0.0000; 0.0328] | -- |
|  | South Korea | 4 (0, 3738) | 0.0000 | [0.0000; 0.0000] | 0.0% |
|  | Japan | 2 (0, 215) | 0.0000 | [0.0000; 0.0085] | 0.0% |
|  | Taiwan | 1 (0, 100) | 0.0000 | [0.0000; 0.0171] | -- |
|  | Mexico | 1 (0, 101) | 0.0000 | [0.0000; 0.0170] | -- |
|  | USA | 1 (0, 106) | 0.0000 | [0.0000; 0.0162] | -- |
|  | Gabon | 1 (0, 109) | 0.0000 | [0.0000; 0.0157] | -- |
|  | Germany | 1 (0, 140) | 0.0000 | [0.0000; 0.0122] | -- |
|  | Emirates | 1 (6, 158) | 0.0380 | [0.0128; 0.0744] | -- |
|  | Ireland | 1 (0, 171) | 0.0000 | [0.0000; 0.0100] | -- |
|  | Czech Republic | 1 (0, 172) | 0.0000 | [0.0000; 0.0100] | -- |
|  | Belgium | 1 (0, 262) | 0.0000 | [0.0000; 0.0066] | -- |
|  | China | 1 (2, 1038) | 0.0019 | [0.0000; 0.0058] | -- |
| Continent | Europe | 5 (0, 752) | 0.0000 | [0.0000; 0.0000] | 0.0% |
|  | Asia | 22 (105, 5651) | 0.0637 | [0.0030; 0.1741] | 96.2% |
|  | South America | 2 (0, 168) | 0.0000 | [0.0000; 0.0080] | 0.0% |
|  | Africa | 9 (72, 635) | 0.1098 | [0.0011; 0.3270] | 97.0% |
|  | Oceania | 1 (0, 52) | 0.0000 | [0.0000; 0.0328] | -- |
|  | North America | 2 (0, 207) | 0.0000 | [0.0000; 0.0093] | 0.0% |
| Time | 2011–2015 | 6 (14, 507) | 0.0119 | [0.0000; 0.1052] | 90.3% |
|  | 2016–2020 | 17 (60, 5411) | 0.0317 | [0.0000; 0.1017] | 94.1% |
|  | 2006–2010 | 5 (17, 484) | 0.0447 | [0.0000; 0.2206] | 93.7% |
|  | 2021–2024 | 2 (70, 92) | 0.7792 | [0.5802; 0.9294] | 76.0% |
|  | Before 2001 | 2 (0, 153) | 0.0000 | [0.0000; 0.0118] | 0.0% |
| Ciprofloxacin |  |  |  |  |  |
| Overall | ND | 16 (95, 1015) | 0.0887 | [0.0508; 0.1341] | 77.2% |
| Country | Nigeria | 2 (6, 44) | 0.1512 | [0.0017; 0.4115] | 47.5% |
|  | Iran | 2 (8, 63) | 0.0934 | [0.0085; 0.2285] | 18.8% |
|  | Ethiopia | 5 (30, 206) | 0.1387 | [0.0924; 0.1918] | 17.3% |
|  | Brazil | 1 (0, 21) | 0.0000 | [0.0000; 0.0803] | -- |
|  | Tanzania | 1 (7, 69) | 0.1014 | [0.0395; 0.1854] | -- |
|  | Trinidad | 1 (6, 72) | 0.0833 | [0.0287; 0.1601] | -- |
|  | Argentina | 1 (2, 84) | 0.0238 | [0.0004; 0.0704] | -- |
|  | China | 1 (23, 91) | 0.2527 | [0.1682; 0.3476] | -- |
| Continent | South Africa | 1 (7, 128) | 0.0547 | [0.0209; 0.1019] | -- |
|  | South Korea | 1 (6, 237) | 0.0253 | [0.0085; 0.0499] | -- |
|  | Africa | 9 (50, 447) | 0.1083 | [0.0668; 0.1569] | 45.6% |
|  | Asia | 4 (37, 391) | 0.0912 | [0.0065; 0.2341] | 92.2% |
|  | South America | 3 (8, 177) | 0.0333 | [0.0019; 0.0890] | 48.3% |
| Time | 2016–2020 | 3 (22, 176) | 0.1069 | [0.0512; 0.1759] | 40.7% |
|  | 2011–2015 | 4 (34, 269) | 0.1096 | [0.0325; 0.2187] | 83.5% |
|  | 2006–2010 | 5 (25, 412) | 0.0670 | [0.0179; 0.1386] | 77.3% |
|  | 2021–2024 | 2 (12, 67) | 0.1792 | [0.0432; 0.3718] | 67.6% |
| Tetracycline |  |  |  |  |  |
| Overall | ND | 65 (4309, 7027) | 0.7780 | [0.7094; 0.8400] | 98.9% |
| Country | Iran | 3 (108, 99) | 0.9495 | [0.8455; 1.0000] | 51.9% |
|  | Turkey | 1 (9, 8) | 0.8889 | [0.5823; 1.0000] | -- |
|  | Uganda | 1 (14, 12) | 0.8571 | [0.6159; 0.9967] | -- |
|  | Algeria | 1 (15, 11) | 0.7333 | [0.4767; 0.9319] | -- |
|  | China | 9 (1059, 834) | 0.8051 | [0.7290; 0.8719] | 85.7% |
|  | Ohangwena | 1 (18, 18) | 1.0000 | [0.9066; 1.0000] | -- |
|  | Yemen | 1 (23, 11) | 0.4783 | [0.2752; 0.6849] | -- |
|  | Nepal | 1 (24, 24) | 1.0000 | [0.9296; 1.0000] | -- |
|  | Tanzania | 1 (28, 15) | 0.5357 | [0.3480; 0.7186] | -- |
|  | Ethiopia | 7 (365, 263) | 0.6806 | [0.5027; 0.8359] | 89.3% |
|  | Nigeria | 1 (33, 33) | 1.0000 | [0.9485; 1.0000] | -- |
|  | Egypt | 1 (36, 29) | 0.8056 | [0.6581; 0.9210] | -- |
|  | USA | 2 (145, 61) | 0.6222 | [0.0037; 1.0000] | 98.7% |
|  | Brazil | 5 (1037, 870) | 0.8137 | [0.7527; 0.8680] | 69.4% |
|  | Senegal | 1 (43, 43) | 1.0000 | [0.9604; 1.0000] | -- |
|  | South Africa | 3 (331, 308) | 0.9555 | [0.8565; 1.0000] | 87.4% |
|  | Australia | 1 (50, 43) | 0.8600 | [0.7481; 0.9445] | -- |
|  | Saudi Arabia | 1 (50, 46) | 0.9200 | [0.8256; 0.9822] | -- |
|  | Italy | 3 (189, 107) | 0.5674 | [0.1623; 0.9255] | 97.3% |
|  | Taiwan | 1 (56, 31) | 0.5536 | [0.4213; 0.6822] | -- |
|  | Africa | 1 (57, 56) | 0.9825 | [0.9262; 1.0000] | -- |
|  | Malaysia | 1 (60, 43) | 0.7167 | [0.5952; 0.8244] | -- |
|  | Trinidad | 2 (138, 132) | 0.9579 | [0.9157; 0.9872] | 0.0% |
|  | South Korea | 3 (226, 152) | 0.7113 | [0.3204; 0.9760] | 97.3% |
|  | Lebanon | 1 (76, 66) | 0.8684 | [0.7820; 0.9364] | -- |
|  | Poland | 1 (82, 3) | 0.0366 | [0.0046; 0.0907] | -- |
|  | Argentina | 1 (84, 18) | 0.2143 | [0.1324; 0.3091] | -- |
|  | Canada | 2 (203, 173) | 0.8543 | [0.7676; 0.9240] | 60.8% |
|  | Switzerland | 1 (124, 110) | 0.8871 | [0.8247; 0.9375] | -- |
|  | Ukraine | 1 (149, 125) | 0.8389 | [0.7752; 0.8940] | -- |
|  | Greece | 1 (171, 169) | 0.9883 | [0.9651; 0.9998] | -- |
|  | Czech Republic | 1 (172, 144) | 0.8372 | [0.7780; 0.8889] | -- |
|  | Ireland | 1 (201, 171) | 0.8507 | [0.7979; 0.8969] | -- |
|  | Spain | 2 (1651, 81) | 0.0711 | [0.0000; 0.3209] | 99.2% |
| Continent | Asia | 24 (1760, 1376) | 0.8240 | [0.7519; 0.8868] | 89.8% |
|  | Africa | 16 (871, 726) | 0.8317 | [0.7130; 0.9254] | 91.9% |
|  | North America | 4 (348, 234) | 0.7468 | [0.3893; 0.9785] | 98.0% |
|  | South America | 8 (1259, 1020) | 0.7878 | [0.6112; 0.9226] | 96.2% |
|  | Oceania | 1 (50, 43) | 0.8600 | [0.7481; 0.9445] | -- |
|  | Europe | 11 (2739, 910) | 0.5748 | [0.2996; 0.8273] | 99.6% |
| Time | 2011–2015 | 13 (1273, 777) | 0.7193 | [0.5573; 0.8573] | 98.1% |
|  | 2016–2020 | 18 (1153, 834) | 0.8048 | [0.6622; 0.9172] | 96.7% |
|  | 2021–2024 | 4 (292, 230) | 0.7355 | [0.4954; 0.9201] | 89.8% |
|  | 2006–2010 | 5 (470, 349) | 0.7310 | [0.4005; 0.9614] | 98.4% |
|  | 2002–2005 | 4 (1556, 228) | 0.5020 | [0.0928; 0.9095] | 99.7% |
|  | Before 2001 | 2 (167, 144) | 0.8802 | [0.7320; 0.9757] | 82.9% |
| Vancomycin |  |  |  |  |  |
| Overall | ND | 106 (153, 21905) | 0.0059 | [0.0008; 0.0141] | 84.7% |
| Country | Iran | 7 (26, 215) | 0.0505 | [0.0000; 0.2107] | 90.1% |
|  | Bosnia and Herzegovina | 1 (0, 7) | 0.0000 | [0.0000; 0.2319] | – |
|  | Turkey | 3 (0, 66) | 0.0000 | [0.0000; 0.0249] | 0.0% |
|  | China | 10 (0, 2597) | 0.0000 | [0.0000; 0.0000] | 0.0% |
|  | Ohangwena | 1 (0, 18) | 0.0000 | [0.0000; 0.0934] | – |
|  | Sri Lanka | 2 (0, 63) | 0.0000 | [0.0000; 0.0271] | 0.0% |
|  | Ethiopia | 11 (31, 501) | 0.0319 | [0.0054; 0.0729] | 74.7% |
|  | Brazil | 8 (0, 1309) | 0.0000 | [0.0000; 0.0000] | 0.0% |
|  | Yemen | 1 (0, 23) | 0.0000 | [0.0000; 0.0735] | – |
|  | Egypt | 8 (16, 350) | 0.0205 | [0.0000; 0.1020] | 86.7% |
|  | Nepal | 1 (6, 24) | 0.2500 | [0.0938; 0.4453] | – |
|  | Palestine | 1 (13, 24) | 0.5417 | [0.3384; 0.7385] | – |
|  | South Korea | 6 (0, 4237) | 0.0000 | [0.0000; 0.0000] | 0.0% |
|  | Lebanon | 1 (0, 31) | 0.0000 | [0.0000; 0.0547] | – |
|  | Nigeria | 2 (25, 71) | 0.2351 | [0.0000; 0.9652] | 98.1% |
|  | Vietnam | 1 (6, 33) | 0.1818 | [0.0658; 0.3343] | – |
|  | USA | 5 (12, 1075) | 0.0152 | [0.0000; 0.1126] | 91.8% |
|  | India | 1 (0, 40) | 0.0000 | [0.0000; 0.0425] | – |
|  | Senegal | 1 (0, 43) | 0.0000 | [0.0000; 0.0396] | – |
|  | South Africa | 4 (9, 532) | 0.0123 | [0.0000; 0.0531] | 80.2% |
|  | Australia | 3 (0, 1196) | 0.0000 | [0.0000; 0.0001] | 0.0% |
|  | Saudi Arabia | 3 (0, 288) | 0.0000 | [0.0000; 0.0055] | 0.0% |
|  | Italy | 3 (5, 3610) | 0.0087 | [0.0000; 0.0765] | 90.7% |
|  | New Zealand | 1 (0, 52) | 0.0000 | [0.0000; 0.0328] | – |
|  | Lisbon | 1 (0, 54) | 0.0000 | [0.0000; 0.0316] | – |
|  | Taiwan | 2 (0, 156) | 0.0000 | [0.0000; 0.0118] | 0.0% |
|  | Malaysia | 1 (0, 60) | 0.0000 | [0.0000; 0.0285] | – |
|  | Thailand | 1 (0, 65) | 0.0000 | [0.0000; 0.0263] | – |
|  | Greece | 1 (0, 67) | 0.0000 | [0.0000; 0.0255] | – |
|  | Tanzania | 1 (0, 69) | 0.0000 | [0.0000; 0.0248] | – |
|  | Trinidad | 1 (0, 72) | 0.0000 | [0.0000; 0.0237] | – |
|  | Japan | 1 (0, 76) | 0.0000 | [0.0000; 0.0225] | – |
|  | Ireland | 2 (0, 248) | 0.0000 | [0.0000; 0.0071] | 0.0% |
|  | Argentina | 1 (0, 84) | 0.0000 | [0.0000; 0.0204] | – |
|  | Canada | 3 (0, 404) | 0.0000 | [0.0000; 0.0047] | 0.0% |
|  | Gabon | 1 (0, 109) | 0.0000 | [0.0000; 0.0157] | – |
|  | Switzerland | 1 (0, 124) | 0.0000 | [0.0000; 0.0138] | – |
|  | Emirates | 1 (4, 158) | 0.0253 | [0.0054; 0.0568] | – |
|  | Malasiya | 1 (0, 200) | 0.0000 | [0.0000; 0.0086] | – |
|  | Hungary | 1 (0, 3554) | 0.0000 | [0.0000; 0.0005] | – |
| Continent | Asia | 49 (71, 8531) | 0.0072 | [0.0000; 0.0230] | 84.7% |
|  | Europe | 10 (5, 7664) | 0.0000 | [0.0000; 0.0033] | 64.5% |
|  | Africa | 24 (65, 1518) | 0.0215 | [0.0017; 0.0551] | 87.2% |
|  | South America | 10 (0, 1465) | 0.0000 | [0.0000; 0.0000] | 0.0% |
|  | North America | 8 (12, 1479) | 0.0061 | [0.0000; 0.0448] | 85.7% |
|  | Oceania | 4 (0, 1248) | 0.0000 | [0.0000; 0.0000] | 0.0% |
| Time | 2011–2015 | 18 (17, 5116) | 0.0000 | [0.0000; 0.0065] | 76.0% |
|  | 2016–2020 | 32 (67, 9681) | 0.0161 | [0.0009; 0.0426] | 89.6% |
|  | 2006–2010 | 14 (0, 1289) | 0.0000 | [0.0000; 0.0001] | 0.0% |
|  | 2002–2005 | 5 (0, 398) | 0.0000 | [0.0000; 0.0029] | 0.0% |
|  | 2021–2024 | 6 (49, 271) | 0.1407 | [0.0131; 0.3532] | 93.6% |
|  | Before 2001 | 8 (0, 1433) | 0.0000 | [0.0000; 0.0001] | 0.0% |
| Tigecycline |  |  |  |  |  |
| Overall | ND | 4 (1, 278) | 0.0012 | [0.0000; 0.0129] | 0 |
| Country | Nigeria | 1 (28, 38) | 0.7368 | [0.5838; 0.8664] | – |
|  | Brazil | 1 (0, 39) | 0.0000 | [0.0000; 0.0436] | – |
|  | South Africa | 1 (12, 49) | 0.2449 | [0.1333; 0.3762] | – |
|  | Trinidad | 1 (5, 72) | 0.0694 | [0.0201; 0.1417] | – |
|  | Japan | 1 (0, 76) | 0.0000 | [0.0000; 0.0225] | – |
|  | USA | 1 (0, 117) | 0.0000 | [0.0000; 0.0146] | – |
| Continent | Africa | 2 (40, 87) | 0.4873 | [0.0675; 0.9185] | 95.4% |
|  | South America | 2 (5, 111) | 0.0256 | [0.0000; 0.1304] | 75.2% |
|  | Asia | 1 (0, 76) | 0.0000 | [0.0000; 0.0225] | – |
|  | North America | 1 (0, 117) | 0.0000 | [0.0000; 0.0146] | – |
| Time | 2021–2024 | 1 (28, 38) | 0.7368 | [0.5838; 0.8664] | – |
|  | 2016–2020 | 2 (17, 121) | 0.1439 | [0.0169; 0.3524] | 85.9% |
|  | Before 2001 | 1 (0, 117) | 0.0000 | [0.0000; 0.0146] | – |
| Cefuroxime |  |  |  |  |  |
| Overall | ND | 8 (43, 3914) | 0.0575 | [0.0000; 0.2278] | 96.7% |
| Country | Nigeria | 2 (27, 44) | 0.3178 | [0.0000; 0.9986] | 93.0% |
|  | Jordan | 1 (0, 29) | 0.0000 | [0.0000; 0.0585] | – |
|  | South Africa | 1 (12, 49) | 0.2449 | [0.1333; 0.3762] | – |
|  | Malaysia | 1 (0, 60) | 0.0000 | [0.0000; 0.0285] | – |
|  | Trinidad | 1 (4, 72) | 0.0556 | [0.0122; 0.1227] | – |
|  | USA | 1 (0, 106) | 0.0000 | [0.0000; 0.0162] | – |
|  | Hungary | 1 (0, 3554) | 0.0000 | [0.0000; 0.0005] | – |
| Continent | Africa | 3 (39, 93) | 0.3016 | [0.0014; 0.7611] | 92.4% |
|  | Asia | 2 (0, 89) | 0.0000 | [0.0000; 0.0201] | 0.0% |
|  | South America | 1 (4, 72) | 0.0556 | [0.0122; 0.1227] | – |
|  | North America | 1 (0, 106) | 0.0000 | [0.0000; 0.0162] | – |
|  | Europe | 1 (0, 3554) | 0.0000 | [0.0000; 0.0005] | – |
| Time | 2016–2020 | 3 (16, 127) | 0.0927 | [0.0005; 0.2670] | 78.9% |
|  | 2021–2024 | 1 (27, 38) | 0.7105 | [0.5548; 0.8453] | – |
|  | 2006–2010 | 1 (0, 106) | 0.0000 | [0.0000; 0.0162] | – |
|  | 2011–2015 | 1 (0, 3554) | 0.0000 | [0.0000; 0.0005] | – |
| Ofloxacin |  |  |  |  |  |
| Overall | ND | 5 (10, 1077) | 0.0640 | [0.0000; 0.3542] | 91.7% |
| Country | Nigeria | 2 (5, 44) | 0.2741 | [0.0000; 1.0000] | 95.6% |
|  | Turkey | 1 (0, 24) | 0.0000 | [0.0000; 0.0704] | – |
|  | Taiwan | 1 (5, 56) | 0.0893 | [0.0261; 0.1805] | – |
| Continent | Poland | 1 (0, 953) | 0.0000 | [0.0000; 0.0018] | – |
|  | Africa | 2 (5, 44) | 0.2741 | [0.0000; 1.0000] | 95.6% |
|  | Asia | 3 (5, 1033) | 0.0107 | [0.0000; 0.0936] | 89.8% |
| Time | 2016–2020 | 1 (5, 6) | 0.8333 | [0.4140; 1.0000] | – |
|  | 2002–2005 | 2 (5, 80) | 0.0359 | [0.0000; 0.1647] | 68.3% |
|  | 2021–2024 | 1 (0, 38) | 0.0000 | [0.0000; 0.0448] | – |
| Rifampin |  |  |  |  |  |
| Overall | ND | 5 (4, 1730) | 0.0028 | [0.0000; 0.0195] | 74.5% |
| Country | Iran | 1 (3, 60) | 0.0500 | [0.0064; 0.1229] | – |
|  | Argentina | 1 (1, 84) | 0.0119 | [0.0000; 0.0504] | – |
|  | Gabon | 1 (0, 109) | 0.0000 | [0.0000; 0.0157] | – |
|  | Ireland | 1 (0, 201) | 0.0000 | [0.0000; 0.0085] | – |
|  | Spain | 1 (0, 1276) | 0.0000 | [0.0000; 0.0013] | – |
| Continent | Asia | 1 (3, 60) | 0.0500 | [0.0064; 0.1229] | – |
|  | South America | 1 (1, 84) | 0.0119 | [0.0000; 0.0504] | – |
|  | Africa | 1 (0, 109) | 0.0000 | [0.0000; 0.0157] | – |
|  | Europe | 2 (0, 1477) | 0.0000 | [0.0000; 0.0006] | 0.0% |
| Time | 2021–2024 | 1 (3, 60) | 0.0500 | [0.0064; 0.1229] | – |
|  | 2002–2005 | 1 (0, 1276) | 0.0000 | [0.0000; 0.0013] | – |
| Clarithromycin |  |  |  |  |  |
| Overall | ND | 4 (196, 740) | 0.3654 | [0.1043; 0.6781] | 96.8% |
| Country | China | 1 (44, 56) | 0.7857 | [0.6675; 0.8845] | – |
|  | Italy | 1 (8, 73) | 0.1096 | [0.0466; 0.1929] | – |
|  | Japan | 1 (32, 76) | 0.4211 | [0.3118; 0.5342] | – |
|  | UK | 1 (112, 535) | 0.2093 | [0.1759; 0.2449] | – |
| Continent | Asia | 2 (76, 132) | 0.6092 | [0.2474; 0.9142] | 94.6% |
|  | Europe | 2 (120, 608) | 0.1652 | [0.0821; 0.2694] | 77.2% |
| Time | 2011–2015 | 2 (156, 591) | 0.4910 | [0.0280; 0.9653] | 98.7% |
| Meropenem |  |  |  |  |  |
| Overall | ND | 12 (0, 5547) | 0 | 0 | 0 |
| Country | Turkey | 2 (0, 33) | 0.0000 | [0.0000; 0.0511] | 0.0% |
|  | Nigeria | 1 (0, 33) | 0.0000 | [0.0000; 0.0515] | – |
|  | Trinidad | 1 (0, 72) | 0.0000 | [0.0000; 0.0237] | – |
|  | South Korea | 3 (0, 3662) | 0.0000 | [0.0000; 0.0000] | 0.0% |
|  | Japan | 3 (0, 692) | 0.0000 | [0.0000; 0.0016] | 0.0% |
|  | China | 1 (0, 102) | 0.0000 | [0.0000; 0.0168] | – |
|  | Poland | 1 (0, 953) | 0.0000 | [0.0000; 0.0018] | – |
| Continent | Asia | 11 (0, 5475) | 0.0000 | [0.0000; 0.0000] | 0.0% |
|  | South America | 1 (0, 72) | 0.0000 | [0.0000; 0.0237] | – |
| Time | 2002–2005 | 1 (0, 24) | 0.0000 | [0.0000; 0.0704] | – |
|  | 2006–2010 | 1 (0, 139) | 0.0000 | [0.0000; 0.0123] | – |
|  | 2016-2020 | 5 (0, 3676) | 0 | 0 | – |
| Cefepime |  |  |  |  |  |
| Overall | ND | 13 (59, 5179) | 0.0254 | [0.0000; 0.1135] | 95.8% |
| Country | Bosnia and Herzegovina | 1 (0, 7) | 0.0000 | [0.0000; 0.2319] | – |
|  | Egypt | 3 (0, 112) | 0.0000 | [0.0000; 0.0170] | 0.0% |
|  | Ethiopia | 2 (56, 95) | 0.5896 | [0.4878; 0.6878] | 0.0% |
|  | Saudi Arabia | 1 (0, 50) | 0.0000 | [0.0000; 0.0341] | – |
|  | South Korea | 3 (0, 3662) | 0.0000 | [0.0000; 0.0000] | 0.0% |
|  | Lebanon | 1 (0, 76) | 0.0000 | [0.0000; 0.0225] | – |
|  | Japan | 1 (0, 139) | 0.0000 | [0.0000; 0.0123] | – |
|  | China | 1 (3, 1038) | 0.0029 | [0.0004; 0.0073] | – |
| Continent | Europe | 1 (0, 7) | 0.0000 | [0.0000; 0.2319] | – |
|  | Asia | 9 (3, 5024) | 0.0000 | [0.0000; 0.0002] | 26.6% |
|  | Africa | 3 (56, 148) | 0.3184 | [0.0001; 0.8243] | 97.8% |
|  | 2011–2015 | 1 (0, 7) | 0.0000 | [0.0000; 0.2319] | – |
|  | 2021–2024 | 1 (21, 36) | 0.5833 | [0.4174; 0.7405] | – |
|  | 2016–2020 | 6 (38, 4795) | 0.0297 | [0.0000; 0.1906] | 97.2% |
|  | 2006–2010 | 2 (0, 215) | 0.0000 | [0.0000; 0.0085] | 0.0% |
| Nitrofurantoin |  |  |  |  |  |
| Overall | ND | 7 (41, 465) | 0.0445 | [0.0000; 0.1606] | 93.7% |
| Country | Iran | 1 (0, 7) | 0.0000 | [0.0000;0.2319] | – |
|  | Jordan | 1 (2, 40) | 0.0500 | [0.0009;0.1448] | – |
|  | Saudi Arabia | 1 (0, 50) | 0.0000 | [0.0000;0.0341] | – |
|  | Italy | 1 (3, 63) | 0.0476 | [0.0061;0.1172] | – |
|  | Trinidad | 1 (33, 72) | 0.4583 | [0.3442;0.5747] | – |
|  | Argentina | 1 (0, 84) | 0.0000 | [0.0000;0.0204] | – |
|  | Ukraine | 1 (3, 149) | 0.0201 | [0.0025;0.0504] | – |
| Continent | Asia | 3 (2, 97) | 0.0041 | [0.0000;0.0620] | 30.5% |
|  | Europe | 2 (6, 212) | 0.0272 | [0.0060;0.0597] | 17.6% |
|  | South America | 2 (33, 156) | 0.1464 | [0.0000;0.7772] | 98.7% |
| Time | 2011–2015 | 1 (3, 63) | 0.0476 | [0.0061;0.1172] | – |
|  | 2016–2020 | 2 (35, 112) | 0.2238 | [0.0000;0.6981] | 96.1% |
|  | 2021–2024 | 1 (3, 149) | 0.0201 | [0.0025;0.0504] | – |
| Lincomycin |  | 1 (0, 7) |  |  |  |
| Overall | ND | 3 (92, 458) | 0.3124 | [0.0312; 0.6993] | 97.9% |
| Country | France | 1 (2, 10) | 0.2000 | [0.0054;0.5132] | – |
|  | Italy | 1 (49, 73) | 0.6712 | [0.5587;0.7749] | – |
|  | Spain | 1 (41, 375) | 0.1093 | [0.0796;0.1431] | – |
| Continent | Europe | 3 (92, 458) | 0.3124 | [0.0312;0.6993] | 97.9% |
| Time | 2016–2020 | 1 (2, 10) | 0.2000 | [0.0054;0.5132] | – |
|  | 2011–2015 | 1 (41, 375) | 0.1093 | [0.0796;0.1431] | – |
| Quinupristin-Dalfopristin |  |  |  |  |  |
| Overall | ND | 7 (18, 484) | 0.0098 | [0.0000; 0.0606] | 88.9% |
| Country | Nigeria | 1 (0, 33) | 0.0000 | [0.0000;0.0515] | – |
|  | Iran | 1 (0, 41) | 0.0000 | [0.0000;0.0415] | – |
|  | China | 2 (0, 136) | 0.0000 | [0.0000;0.0132] | 0.0% |
|  | Italy | 1 (18, 73) | 0.2466 | [0.1537;0.3526] | – |
|  | Argentina | 1 (0, 84) | 0.0000 | [0.0000;0.0204] | – |
|  | USA | 1 (0, 117) | 0.0000 | [0.0000;0.0146] | – |
| Continent | Asia | 4 (0, 210) | 0.0000 | [0.0000;0.0090] | 0.0% |
|  | Europe | 1 (18, 73) | 0.2466 | [0.1537;0.3526] | – |
|  | South America | 1 (0, 84) | 0.0000 | [0.0000;0.0204] | – |
|  | North America | 1 (0, 117) | 0.0000 | [0.0000;0.0146] | – |
| Time | 2016–2020 | 1 (0, 33) | 0.0000 | [0.0000;0.0515] | – |
|  | 2011–2015 | 1 (0, 91) | 0.0000 | [0.0000;0.0188] | – |
|  | Before 2001 | 1 (0, 117) | 0.0000 | [0.0000;0.0146] | – |
